# Supplementary material for: Increased Serological Response Against Human Herpesvirus 6A Is Associated With Risk for Multiple Sclerosis
Source: Front Immunol. 2019 Nov 26;10:2715. doi: 10.3389/fimmu.2019.02715 (PMC6988796; doi:10.3389/fimmu.2019.02715)
Supplement: Supplementary file 1 [file Presentation_1.pdf]

Supplementary Material

1 Supplementary Figures and Tables for

Increased serological response against human herpesvirus 6A is associated with increased risk for multiple sclerosis

Elin Engdahl[1,a], Rasmus Gustafsson[1,a], Jesse Huang[1,a], Martin Biström[2], Izaura Lima Bomfim[1], Pernilla Stridh[1], Mohsen Khademi[1], Nicole Brenner[3], Julia Butt[3], Angelika Michel[3], Daniel Jons[4], Maria Hortlund[5], Lucia Alonso-Magdalena[6], Anna Karin Hedström[1,9], Louis Flamand[7], Masaru Ihira[8], Tetsushi Yoshikawa[8], Oluf Andersen[4], Jan Hillert[1], Lars Alfredsson[9,10], Tim Waterboer[3], Peter Sundström[2,b], Tomas Olsson[1,b], Ingrid Kockum[1,b], Anna Fogdell-Hahn[1,b*]

[1] Department of Clinical Neuroscience, Karolinska Institutet, Stockholm, Sweden AND Center for Molecular Medicine, Stockholm, Sweden

[2] Department of Pharmacology and Clinical Neuroscience, Umeå Universitet, Umeå, Sweden

[3] Infections and Cancer Epidemiology, German Cancer Research Center (DKFZ), Heidelberg, Germany

[4] Department of Clinical Neuroscience, Institute of Neuroscience and Physiology, the Sahlgrenska Academy, University of Gothenburg, Sweden

[5] Department of Laboratory Medicine, Karolinska Institutet, Stockholm, Sweden

[6] Department of Neurology, Skåne University Hospital, Malmö, Sweden

[7] Department of microbiology, infectious disease and immunology, Laval University, Québec city, Québec, Canada

[8] Department of Pediatrics, Fujita Health University School of Medicine, Toyoake, Japan

[9] Institute of Environmental Medicine, Karolinska Institutet, Stockholm, Sweden

[10] Centre for Occupational and Environmental Medicine, Stockholm County Council, Stockholm, Sweden

[a] These authors contributed equally to this work

[b] These authors also contributed equally to this work

*** Correspondence:**
Anna Fogdell-Hahn, Associate Professor
Anna.Fogdell-Hahn@ki.se

Supplementary Figures and Tables contains:

Figure S1. Interaction of antibody response against different herpesviruses in association to MS

Figure S2. Moving OR analysis visualizing MS risk

Figure S3. Median MFI response against HHV-6B proteins IE1B and 101K at different ages.

Figure S4. Manhattan plots IgG serostatus

Figure S5. Manhattan plots of the association results when associated HLA alleles are added as covariates

Figure S6. Correlation between different antibody responses for HHV-6A and B antigens

Figure S7. Correlation between anti-HHV-6A/6B, CMV and EBV serology

Figure S8. Alignment of amino acid sequences used in the multiplex serological assay.

Table S1. Difference in IgG response against investigated HHV-6A/6B proteins between MS cases and controls in the pre-MS cohort

Table S2. Difference in IgG response against investigated HHV-6A/6B proteins between MS cases and controls in the established MS cohort

Table S3. Association between IgG levels and HLA haplotypes and IE1B

Table S4. Association between IgG levels and HLA haplotypes and 101K

Table S5. Anti-IE1A, IE1B, 101K and p100 IgG response in children with primary HHV-6B infection

Table S6. Alignment of amino acid sequences used in the multiplex serological assay.

Table S7. Antibody responses analyzed for association with non-HLA SNPs serolevels

Table S8. Antibody responses analyzed for association with non-HLA SNPs serostatus

Supplementary Figure S1

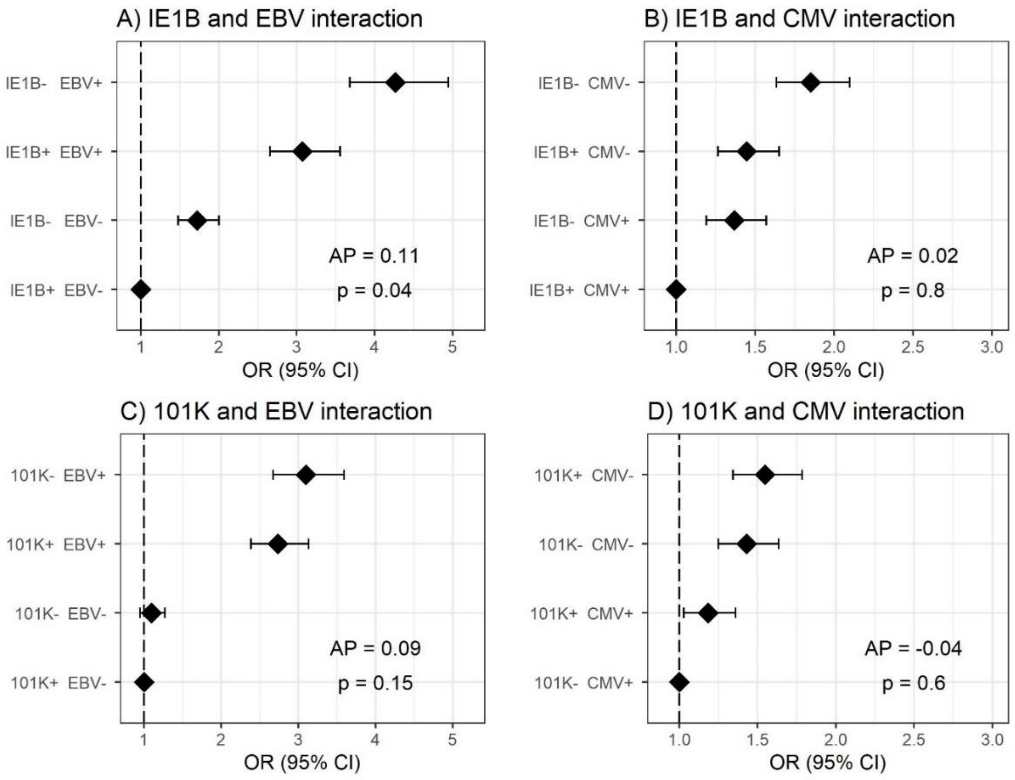

Supplementary Figure S1. Interaction of antibody response against different herpesviruses in association to MS. Odds ratios (OR) and confidence intervals (CI) for A) IE1B and EBV, B) IE1B and CMV, C) 101K and EBV, and D) 101K and CMV were obtained through logistic regression models adjusted for age, sex and cohort type analyzing the Established MS cohort (n=8742 persons with MS and n=7215 controls)**.** OR were calculated in relation to the group with the lowest MS risk. Plus (+) indicates being a strong responder while minus (-) indicates being a weak responder. Strong IE1B / 101K response is defined as having an MFI value being in the upper quartile of measured response, while a low response is having an antibody measurement being in the lower quartile of measured response. Strong EBV / CMV response is defined as having a higher EBV / CMV index than the median among controls, while a weak response is having a lower index compared to the median among controls.

Supplementary Figure S2

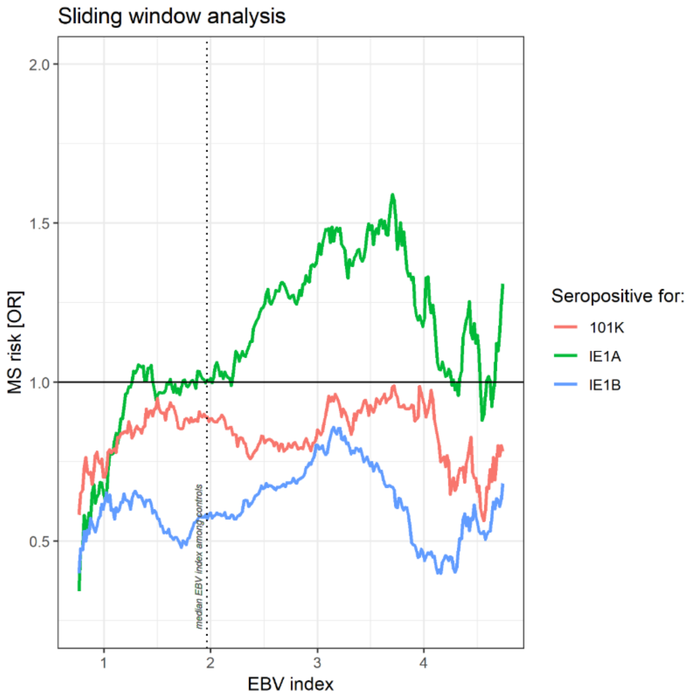

Supplementary Figure S2. **Moving OR analysis visualizing MS risk for high IE1A, IE1B and 101K responders, depending on EBV IgG response.** HHV-6A/-6B associated MS risk calculated using logistic regression analyses adjusted for age, sex and cohort type in a sliding window approach, i.e. stratified for EBV response in the established MS cohort (8742 persons with MS and 7215 controls). Strong responders are defined as those present in the highest quartile of each measured response, and in the regression models strong responders were compared to weak responders, here defined as those present in the lowest quartile of each measured response. Each analysis window contained a span of 0.75 units EBV index and overlapping analyses were performed as the window moved 0.015 units to its next analysis. Windows containing < 100 MS cases or < 100 controls were excluded which left 266 analysis windows, i.e. 266 plotted data points for each antigen response in the graph.

Supplementary Figure S3

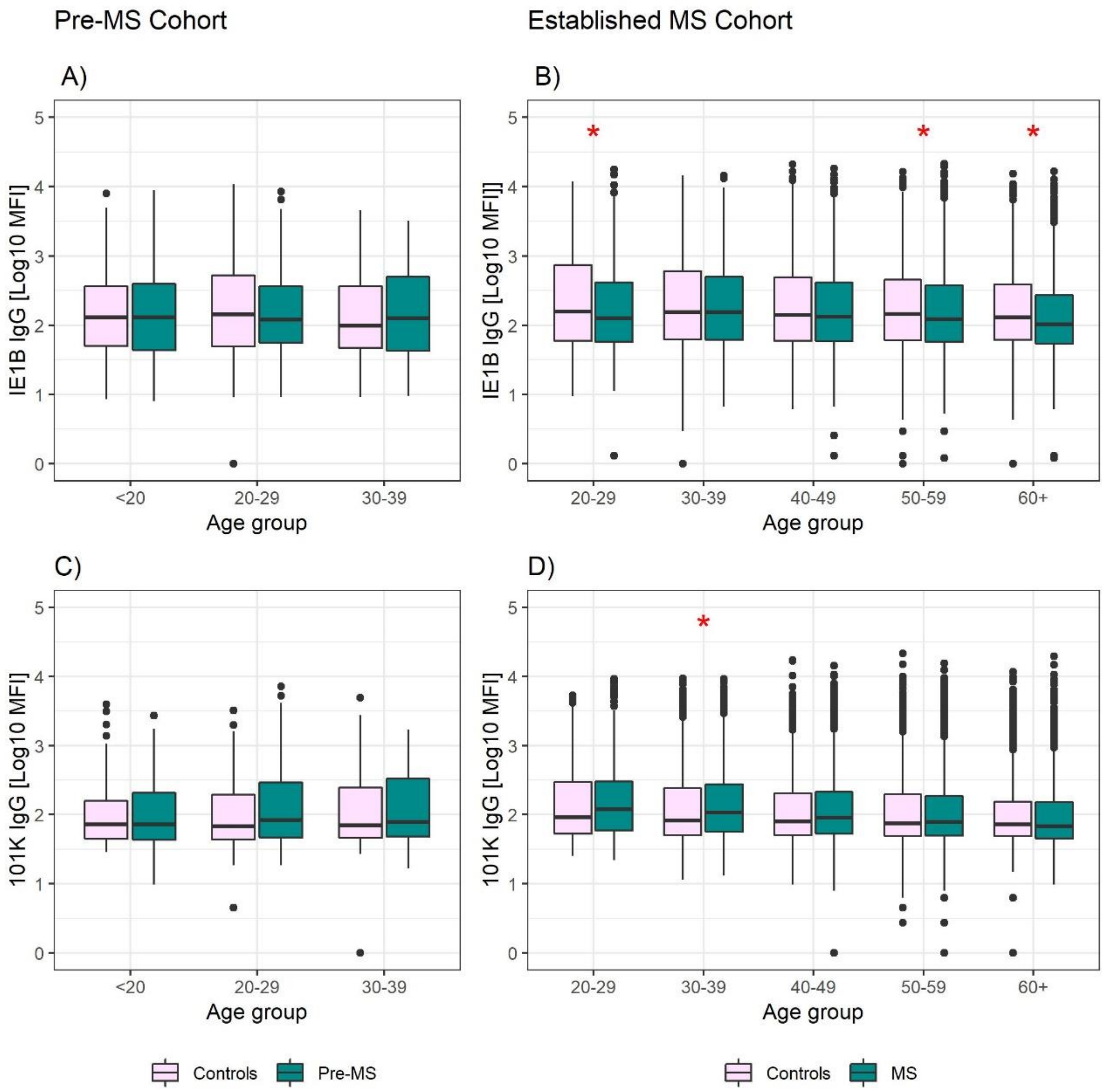

Supplementary Figure S3. Median MFI response against HHV-6B proteins IE1B and 101K in different age groups. Median of median fluorescence intensity (MFI) in different age groups for pre-MS cohort (n=478 persons) who later developed MS (green) and n=476 controls (pink); A, C) and established MS cohort (n=8394 persons with MS (green) and n=7214 controls (pink); B and D) for HHV-6B IE1B IgG (A and B); HHV-6B 101K IgG (C and D). Statistics were calculated with linear regression. Significant ($p<0.008$) differences in IgG levels between MS cases and controls within each age group are indicated with *.

Supplementary Figure S4

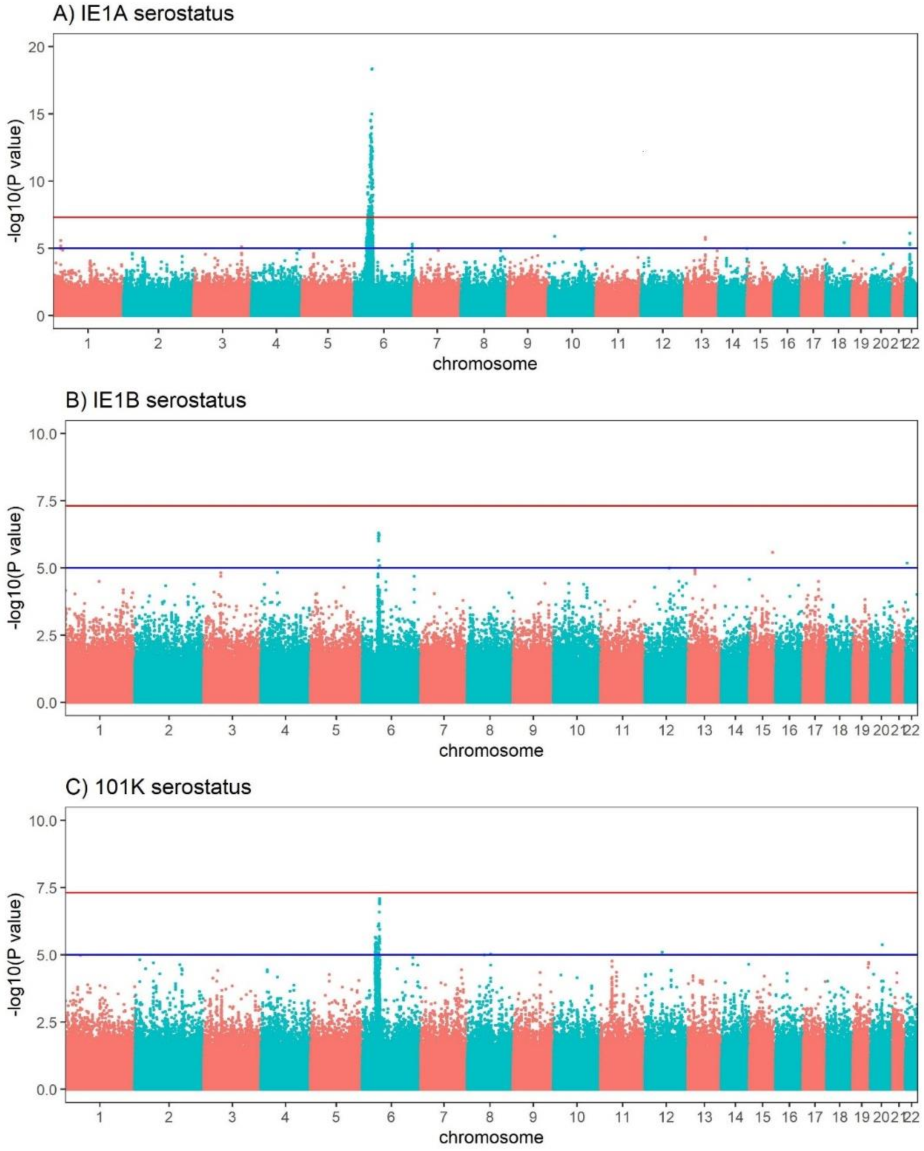

Supplementary Figure S4. Manhattan plots visualizing associations between SNPs and anti-HHV-6A/6B protein IgG serostatus. GWAS data showing associations between SNPs high/low IgG response, against (A) IE1A, (B) IE1B and (C) 101K. Red lines indicate GWAS significance level of 5x10^{-8} (-log10 = 7.3 on the y-axis) and blue lines indicate suggestive association (p=10^{-5}). Association analysis was carried out in established MS cohort with available genotypes (n=6,396 persons with MS and n=5,530 controls).

Supplementary Figure S5

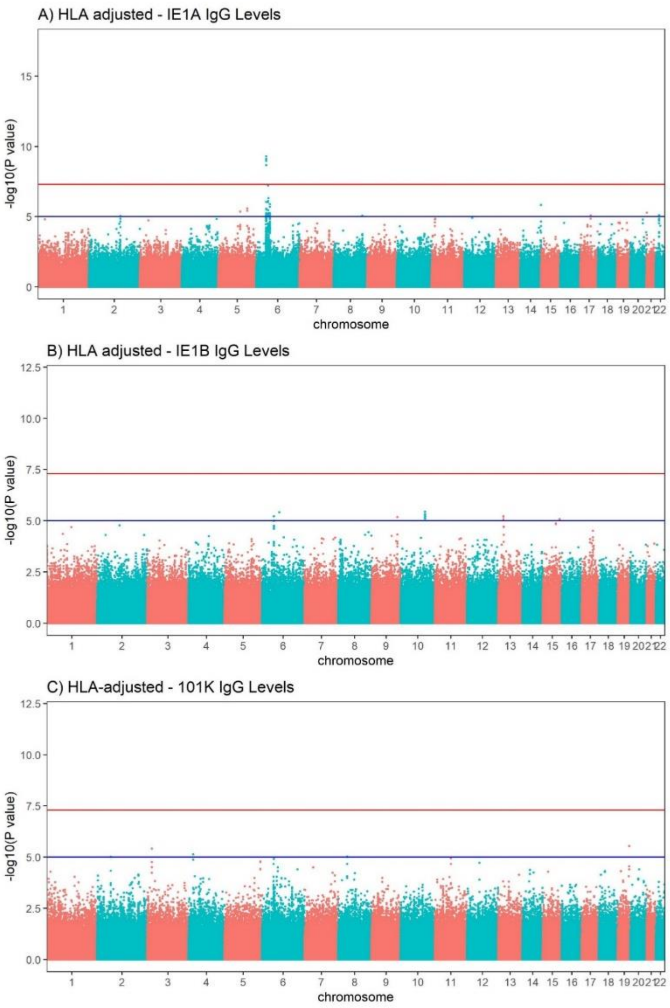

Supplementary Figure S5. Manhattan plots of the association results when associated HLA alleles are added as covariates. GWAS was performed between SNPs and A) IE1A, B) IE1B and C) 101K with linear regression models where HLA alleles associated to the responses were added to the model and adjusted for. Red lines indicate GWAS significance level of 5x10^-8 (-log10 = 7.3 on the y-axis) and blue lines indicate suggestive association ($p=10^{-5}$). In A) SNPs remaining significant after conditionen for HLA are rs1610678, rs1611149, rs173693 and rs915669. Association analysis was carried out in established MS cohort with available genotypes (n=6,396 persons with MS and n=5,530 controls).

Supplementary Figure S6

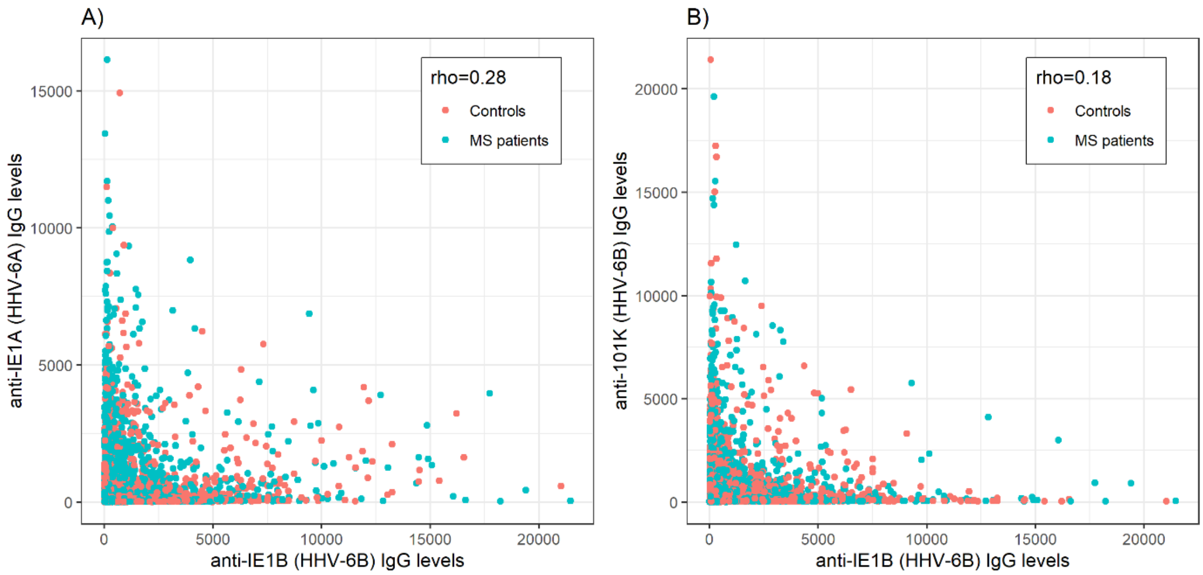

Supplementary Figure S6. Correlation between different antibody responses for HHV-6A and B antigens. Antibody levels measured as Median Fluorescence Intensity [MFI] against (A) IE1B and IE1A, (B) 101K and IE1B are plotted. Rho = Spearman's rank correlation coefficient. There is an inverse correlation between IgG levels against the different HHV-6 antigens, suggesting that different individuals respond with differently to the various antigens. Analysis was carried out in established MS cohort (n=8742 persons with MS and n=7215 controls).

Supplementary Figure S7

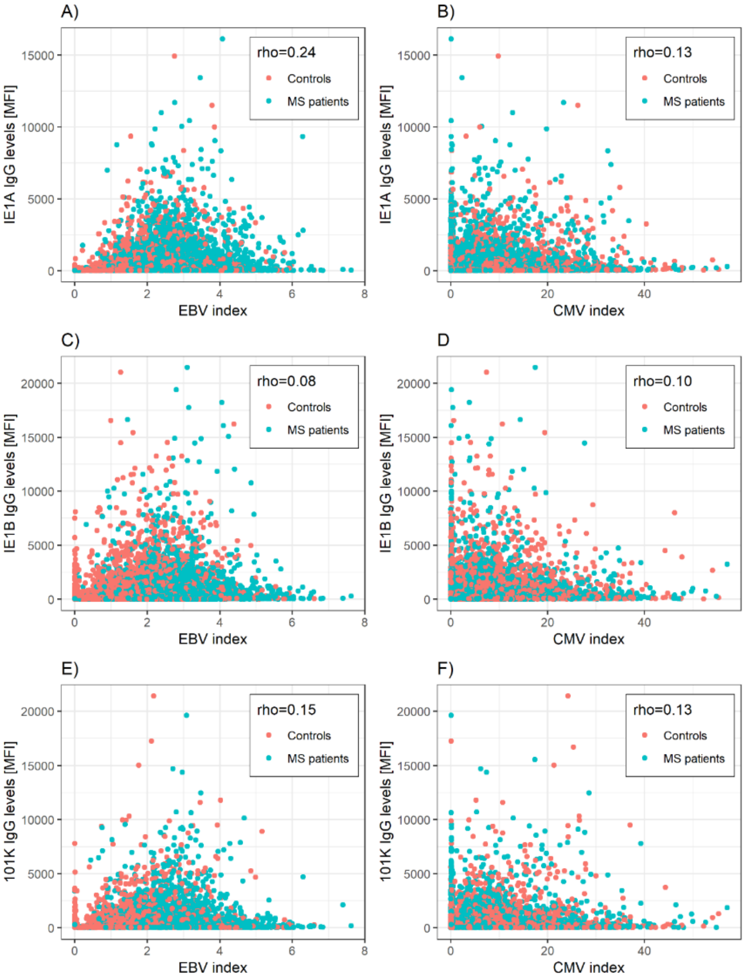

Supplementary Figure S7. Correlation between anti-HHV-6A/6B, CMV and EBV serology. Antibody levels [MFI] against IE1A, IE1B and 101K are plotted against EBV or CMV indexes. Rho = Spearman's rank correlation coefficient**.** There was a significant positive correlations between the different HHV-6 antibody responses and the EBV index (rho $\leq$ 0.23) as well as with the CMV index (rho $\leq$ 0.15). Being a strong IE1A responder was significantly associated with being a strong EBV responder, both in MS cases (OR = 3.41, p = $7x10^{-72}$) and in controls (OR = 2.68, p = $2x10^{-45}$). Analysis was carried out in established MS cohort (n=8742 persons with MS and n=7215 controls).

Supplementary Figure S8

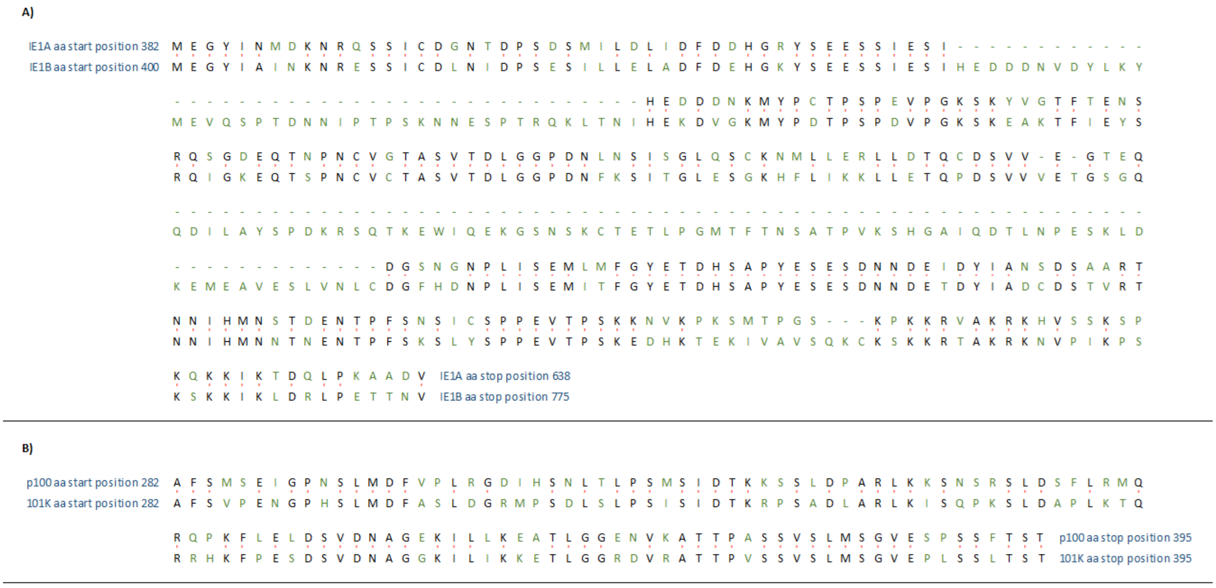

Supplementary Figure S8. Alignment of amino acid sequences used in the multiplex serological assay. Figure adapted from the NCBI BLAST. (A) Alignment of IE1A (Sequence ID = AGJ52065.1; length = 257 aa) with IE1B (NP_050266.1; length = 376 aa), (B) Alignment of p100 (NP_042902.1; length = 114 aa) with 101K (NP_050192.1; length = 114 aa). Identical amino acids from the two sequences are indicated with black font and a red connection between the amino acids. Green font indicates amino acid difference between the two sequences. aa = amino acid.

Table S1. Difference in IgG response against investigated HHV-6A/6B proteins between MS cases and controls in the pre-MS cohort

	Model	-	Mann Whitney	Linear regression									
	Included	All measurements											
	Adjustment	-		-		AGE, SEX		HLA		EBV & CMV		ALL	
		Median MFI	p	β	p	β	p	β	p	β	p	β	p
IE1A	All subjects	46	**2E-08**	0.18	**7E-10**	0.18	**6E-10**			0.14	**3E-06**	0.14	**2E-06**
	Men	46	4E-02	0.15	0.03	0.15	0.02	NA		0.11	0.09	0.12	0.07
	Women	46	**2E-07**	0.19	**8E-09**	0.19	**7E-09**			0.14	**2E-05**	0.14	**1E-05**
IE1B	All subjects	125	8E-01	-0.02	0.67	-0.02	0.67			-0.02	0.7	-0.02	0.6
	Men	130	8E-01	0.00	0.98	-0.01	0.95	NA		0.04	0.7	0.04	0.7
	Women	124	7E-01	-0.02	0.65	-0.02	0.65			-0.04	0.4	-0.04	0.4
101K	All subjects	75	3E-02	0.07	0.02	0.07	0.02			0.06	0.1	0.06	0.1
	Men	58	7E-01	0.08	0.28	0.08	0.28	NA		0.09	0.2	0.09	0.2
	Women	78	4E-02	0.07	0.04	0.07	0.04			0.05	0.2	0.05	0.2

Mann Whitney U tests were performed on un-transformed MFI values but as the antibody levels can be affected by co-variables, the IgG responses were also investigated using linear regression models on Log10-transformed MFI values, adjusted for potential confounders. Measurements of EBV and CMV serology were converted into EBV and CMV indexes, respectively, and added as confounders. Significant p-values are highlighted in bold.

Table S2. Difference in IgG response against investigated HHV-6A/6B proteins between MS cases and controls in the established MS cohort

	Model	-	Mann Whitney	Linear regression									
	Included	All measurements											
	Adjustment	-		-		AGE, SEX, COHORT		HLA *		EBV & CMV #		ALL	
		Median MFI	p	β	p	β	p	β	p	β	p	β	p
IE1A	All subjects	47	**6E-32**	0.11	**4E-33**	0.10	**9E-30**	0.11	**6E-26**	0.05	**7E-09**	0.06	**2E-10**
	Men	51	**8E-15**	0.14	**5E-15**	0.15	**5E-16**	0.14	**2E-11**	0.08	2E-05	0.09	**4E-06**
	Women	47	**3E-19**	0.10	**2E-20**	0.09	**2E-17**	0.10	**5E-16**	0.04	4E-05	0.06	**3E-06**
IE1B	All subjects	123	**1E-08**	- 0.06	**2E-10**	-0.07	**9E-14**	-0.07	**6E-10**	-0.09	**1E-17**	-0.09	**3E-15**
	Men	116	4E-02	- 0.04	3E-02	-0.05	4E-03	-0.05	2E-02	-0.07	3E-04	-0.07	4E-04
	Women	125	**1E-07**	- 0.07	**6E-09**	-0.08	**7E-12**	-0.07	**3E-08**	-0.09	**3E-14**	-0.09	**4E-12**
101K	All subjects	46	7E-04	0.02	2E-02	0.02	4E-02	0.01	4E-01	-0.002	8E-01	-0.003	7E-01
	Men	35	2E-03	0.03	9E-02	0.02	2E-01	3E-04	0.99	0.01	6E-01	-0.02	3E-01
	Women	51	6E-03	0.02	2E-02	0.02	7E-02	0.02	1E-01	-0.001	9E-01	0.002	8E-01

Mann Whitney U tests were performed on un-transformed MFI values, but as the antibody levels can be affected by co-variables the IgG responses were also investigated using linear regression models on Log10-transformed MFI values, adjusted for potential confounders. *Models adjusted for carriage of HLA-DRB1*15:01, HLA-A*02 and HLA alleles associated to the respective measured IgG responses (marked in Table 2). # Measurements of EBV and CMV serology were converted into EBV and CMV indexes, respectively, and added as confounders. Number of individuals included were 15608 (pre-MS excluded), of which 4159 were men and 11449 were women. Significant p-values are highlighted.

Table S3. Association between IgG levels and HLA haplotypes and IE1B

	MS cases				Controls				All subjects			
Adjustment[A]	Adjustment #1		Adjustment #2		Adjustment #1		Adjustment #2		Adjustment #1		Adjustment #2	
	**β**	**p**	**β**	**p**	**β**	**p**	**β**	**p**	**β**	**p**	**β**	**p**
DRB1*13:02-DRB3*03:01-DQB1*06:04												
DRB1*13:02	0.082	0.002	0.076	0.004	0.078	0.003	0.079	0.002	0.079	**2E-05**	0.077	**3E-05**
**DRB3*03:01**	0.082	0.001	0.076	0.003	0.079	0.002	0.08	0.002	0.08	**1E-05**	0.078	**2E-05**
DQB1*06:04	0.103	**4E-04**	0.097	0.001	0.071	0.009	0.074	0.007	0.084	**2E-05**	0.083	**3E-05**
B*44:03-C*16:01-DRB1*07:01-DRB4*01:01-DQA1*02:01-DQB1*02:02												
**B*44:03**	0.071	0.048	0.066	0.066	0.152	**5E-05**	0.153	**5E-05**	0.109	**2E-05**	0.107	**4E-05**
C*16:01	0.071	0.107	0.064	0.146	0.125	0.008	0.123	0.009	0.096	0.003	0.092	0.004
DRB1*07:01	0.004	0.853	0.003	0.89	0.098	**8E-06**	0.1	**5E-06**	0.053	**6E-04**	0.054	**6E-04**
DRB4*01:01	0.024	0.393	0.022	0.43	0.144	**7E-07**	0.146	**5E-07**	0.083	**3E-05**	0.083	**3E-05**
DQA1*02:01	0.016	0.467	0.015	0.497	0.098	**6E-06**	0.101	**4E-06**	0.06	**1E-04**	0.06	**1E-04**
DQB1*02:02	0.011	0.691	0.008	0.753	0.149	**6E-08**	0.15	**5E-08**	0.081	**2E-05**	0.080	**3E-05**
B*07:02-C*07:02												
**B*07:02**	-0.053	**2E-04**	-0.047	**1E-03**	-0.025	0.158	-0.017	0.327	-0.041	**2E-04**	-0.035	**2E-03**
C*07:02	-0.045	0.001	-0.04	0.005	-0.028	0.105	-0.02	0.245	-0.038	**5E-04**	-0.032	**4E-03**
C*15:02												
C*15:02	0.003	0.917	-0.005	0.857	0.148	**8E-05**	0.152	**5E-05**	0.062	0.008	0.059	0.012

The allele variants in a given HLA haplotype are presented. Underlined alleles were conferring the strongest effect, determined by stepwise conditional analyses. HLA genotype data was available for 7,063 MS cases and 6,098 controls. [A]Adjustment #1 = Age, sex, 5 PCA vectors, cohort type (incidence or prevalence). When adjusted for HLA, one associated HLA allele from each associated haplotype (underlined in the

table) were added to the model in order to test for independent association of the investigated HLA allele. Adjustment #2 = Adjustment #1 + HLA. When all subjects were analyzed together, MS affection status were added as a covariable. Haplotypes associated to IE1B indicated in bold if any allele in it has a p-value <0.001.

Table S4. Association between IgG levels and HLA haplotypes and 101K

	**MS cases**				**Controls**				**All subjects**			
Adjustment[A]	Adjustment #1		Adjustment #2		Adjustment #1		Adjustment #2		Adjustment #1		Adjustment #2	
	**β**	**p**	**β**	**p**	**β**	**p**	**β**	**p**	**β**	**p**	**β**	**p**
B*40:01-DRB1*09:01												
**B*40:01**	-0.07	**2E-04**	-0.06	0.001	-0.07	**1E-04**	-0.06	**8E-04**	-0.07	**6E-08**	-0.06	**3E-06**
DRB1*09:01	-0.06	0.158	-0.05	0.234	-0.11	0.003	-0.10	0.006	-0.09	0.001	-0.08	0.004
A*02:01-B*44:02-C*05:01												
**A*02:01**	-0.05	**2E-04**	-0.04	0.000	-0.03	0.012	-0.03	0.010	-0.04	**6E-06**	-0.04	**1E-05**
B*44:02	-0.04	0.025	-0.04	0.023	-0.04	0.019	-0.04	0.011	-0.04	**1E-03**	-0.04	**5E-04**
C*05:01	-0.04	0.031	-0.04	0.025	-0.05	0.003	-0.05	0.002	-0.05	**3E-04**	-0.05	**9E-05**
DRB1*04-DQA1*03:01-DQB1*03:02												
DRB1*04	0.06	**1E-05**	0.06	**3E-06**	0.02	0.245	0.02	0.074	0.04	**8E-05**	0.04	**3E-06**
DQA1*03:01	0.06	**2E-05**	0.06	**3E-06**	0.01	0.303	0.02	0.104	0.04	**2E-04**	0.04	**9E-06**
**DQB1*03:02**	0.07	**2E-06**	0.07	**4E-07**	0.03	0.059	0.04	0.014	0.05	**3E-06**	0.05	**9E-08**
DRB1*13:02-DRB3*03:01-DQA1*01:02-DQB1*06:04												

DRB1*13:02	-0.05	0.033	-0.01	0.631	-0.05	0.011	-0.01	0.623	-0.05	**8E-04**	-0.01	0.479
DRB3*03:01	-0.05	0.036	-0.01	0.648	-0.05	0.013	-0.01	0.661	-0.05	**1E-03**	-0.01	0.529
DQA1*01:02	-0.03	0.006	-0.01	0.293	-0.03	0.014	-0.01	0.352	-0.03	0.000	-0.01	0.168
**DQB1*06:04**	-0.07	0.004	-0.03	0.327	-0.06	0.004	-0.02	0.437	-0.07	**4E-05**	-0.02	0.199
DRB1*08:01-DQA1*04:01-DQB1*04:02												
DRB1*08:01	0.02	0.451	0.04	0.089	0.09	**1E-04**	0.10	**1E-05**	0.05	0.001	0.07	**2E-05**
DQA*04:01	0.01	0.561	0.03	0.136	0.09	**7E-05**	0.10	**8E-06**	0.05	0.002	0.06	**4E-05**
**DQB*04:02**	0.02	0.294	0.04	0.051	0.09	**5E-05**	0.10	**6E-06**	0.05	**5E-04**	0.07	**8E-06**
A*03:01												
A*03:01	0.03	0.023	0.02	0.122	0.04	0.011	0.03	0.064	0.03	**6E-04**	0.02	0.017
C*03:03												
C*03:03	0.07	**2E-04**	0.06	0.003	0.04	0.037	0.03	0.094	0.06	**5E-05**	0.05	1E-03

The allele variants in a given HLA haplotype are presented. Underlined alleles were conferring the strongest effect, determined by stepwise conditional analyses. HLA genotype data was available for 7,063 MS cases and 6,098 controls. [A]Adjustment #1 = Age, sex, 5 PCA vectors, cohort type (incidence or prevalence). When adjusted for HLA, one associated HLA allele from each associated haplotype (underlined in the table) were added to the model in order to test for independent association of the investigated HLA allele. Adjustment #2 = Adjustment #1 + HLA. When all subjects were analyzed together, MS affection status were added as a covariable. Haplotypes associated to 101K are shown if any allele in it has a p-value <0.001 indicated in bold.

Table S5. Anti-IE1A, IE1B, 101K and p100 IgG response in children with primary HHV-6B infection

									IE1A		IE1B		101K		p100	
no	Age	Sex	Time of sampling *	Sample type	Isolation of HHV-6 from blood	Detection of HHV-6 DNA in plasma/ serum ^	viral species	anti-HHV-6 IgG titer [IFA] ~	MFI	Post/Pre	MFI	Post/Pre	MFI	Post/Pre	MFI	Post/Pre
1	12 months	F	Acute	plasma	+	NT	B	4	97		26		1		3	
			+ 48 days	plasma	NT	NT		256	80	0.8	7	0.3	69	**69.4**	11	4.3
2	12 months	M	Acute	serum	+	NT	B	NT	210		50		15		5	
			+ 28 days	serum	NT	NT		NT	176	0.8	612	**12.2**	249	**16.2**	13	2.6
3	8 months	M	Acute	serum	+	NT	B	4	91		77		3		4	
			+ 16 days	serum	NT	NT		128	109	1.2	86	1.1	71	**22.8**	1	0.3
4	10 months	F	Acute	plasma	+	NT	B	4	100		74		82		10	
			+ 14 days	serum	NT	NT		128	106	1.1	26	0.4	186	2.3	11	1.1
5	14 months	F	Acute	plasma	+	+	B	8	114		42		10		1	
			+ 8 days	serum	NT	NT		NT	407	3.6	1466	**35.3**	101	**10.5**	10	9.5
6	14 months	F	Acute	plasma	NT	+	B	NT	88		16		2		14	
			+ 7 days	plasma	NT	NT		256	152	1.7	23	1.5	34	**16.4**	5	0.4
7	12 months	M	Acute	plasma	NT	+	B	16	324		105		7		29	
			+ 6 days	serum	NT	NT		256	190	0.6	71	0.7	35	4.9	1	0.04
8	15 months	M	Acute	serum	+	+	B	8	252		29		1		2	
			+ 6 days	serum	NT	NT		32	313	1.2	43	1.5	1	1.0	1	0.6
9	11 months	F	Acute	plasma	+	+	B	4	140		60		75		15	
			+ 6 days	plasma	NT	NT		16	104	0.7	29	0.5	56	0.7	15	1.0
10	12 months	M	Acute	plasma	+	+	B	NT	74		27		3		16	
			+ 5 days	plasma	NT	NT		NT	84	1.1	22	0.8	32	9.5	10	0.7

The first sample (Pre) is collected during Exanthema subitum (ES) acute phase, the second sample (Post) is collected in the convalescent phase and days between the two samples are given in the table. When ≤ 7 days between the samples, the samples are marked grey. NT = not tested. Isolation of viable HHV-6 from blood is described in (Asano Y, et al 1989 journal of pediatrics114:535-539). ^ Presence of DNA in cell-free plasma/serum was detected using the LAMP method (ref: Ihira 2004). If isolation of HHV-6 from blood sample was positive, the identification of HHV-6 species was performed by specific immunofluorescence staining with monoclonal antibodies to HHV-6 (Asano Y, et al 1989 journal of pediatrics114:535-539) and if detection of HHV-6 DNA from serum using LAMP was positive, the identification of HHV-6 species was followed RFLP method by LAMP products. (Ihira et al J virol Methods 2008 154(1-2):223-225). ~ IFA was used for detection of antibody against the representative strain of HHV-6B isolated (FG-1) from patients with ES (Suga et al 1989 pediatrics 83(6)1003-6.). A serum dilution of 1:100 was used in the assay and with that dilution MFI values <50 may be in the technical noise of the assay and should be interpreted with caution. The ability of this secondary antibody to detect IgM is limited, but we cannot be excluded

Table S6. Amino acid sequences used as antigens in the multiplex serological assay.

Virus species	Antigen used (Sequence ID)	Protein sequence length	Amino acid start and stop position	Sequence	Most similar protein from another virus species (amino acid identity and Sequence ID)
**HHV-6A**	IE1A (AGJ52065.1)	257	382-638	MEGYINMDKNRQSSICDGNTDPSDSMILDLIDFDDHGRYSEESSI<br>ESIHEDDDNKMYPCTPSPEVPGKSKYVGTFTENSRQSGDEQTNP<br>NCVGTASVTDLGGPDNLNSISGLQSCKNMLLERLLDTQCDSVVE<br>GTEQDGSNGNPLISEMLMFGYETDHSAPYESESDNNDEIDYIAN<br>SDSAARTNNIHMNSTDENTPFSNSICSPPEVTPSKKNVKPKSMTP<br>GSKPKKRVAKRKHVSSKSPKQKKIKTDQLPKAADV	**IE1B**<br>(98/180 [54%] AAP81093.1)<br>(97/176 [55%] NP_050266.1)
**HHV-6B**	IE1B (NP_050266.1)	376	400-775	MEGYIAINKNRESSICDLNIDPSESILLELADFDEHGKYSEESSIESI<br>HEDDDNVDYLKYMEVQSPTDNNIPTPSKNNESPTRQKLTNIHE<br>KDVGKMYPDTPSPDVPGKSKEAKTFIEYSRQIGKEQTSPNCVCT<br>ASVTDLGGPDNFKSITGLESGKHFLIKKLLETQPDSVVVETGSGQ<br>QDILAYSPDKRSQTKEWIQEKGSNSKCTETLPGMTFTNSATPVK<br>SHGAIQDTLNPESKLDKEMEAVESLVNLCDGFHDNPLISEMITF	**IE1A**<br>(94/173 [54%] CAA58338.1)<br>(93/173 [54%] AGJ52065.1)

				GYETDHSAPYESESDNNDETDYIADCDSTVRTNNIHMNNTNE<br>NTPFSKSLYSPPEVTPSKEDHKTEKIVAVSQKCKSKKRTAKRKN<br>VPIKPSKSKKIKLDRLPETTNV	
**HHV-6B**	101K<br>(NP_050192.1)	114	282-395	AFSVPENGPHSLMDFASLDGRMPSDLSLPSISIDTKRPSADLA<br>RLKISQPKSLDAPLKTQRRHKFPESDSVDNAGGKILIKKETLGG<br>RDVRATTPVSSVSLMSGVEPLSSLTST	**p100**<br>(81/114 [71%] AKZ18118.1)<br>(74/114 [65%] NP_042902.1)
**HHV-6A**	p100<br>(NP_042902.1)	114	282-395	AFSMSEIGPNSLMDFVPLRGDIHSNLTLPSMSIDTKKSSLDPA<br>RLKKSNSRSLDSFLRMQRQPKFLELDSVDNAGEKILLKEATLG<br>GENVKATTPASSVSLMSGVESPSSFTST	**101K**<br>(74/114 [65%] NP_050192.1)

Amino acid similarity to the top-scored protein from another virus species, obtained with the NCBI BLAST function. If more than one Sequence IDs are given, the second ID is of special interest. The % identity is given by the protein BLAST matching, please note that the matched amino acid length is not always the same as the length as the used protein sequence.

Table S7. Antibody responses analyzed for association with non-HLA SNPs serolevels

Antigen	Subject group	Chromosome	SNP marker	Basepair position	Minor allele	β	p
IE1A	MS cases	2	RS17450916	205 697 101	A	-0.06911	2.58E-06
IE1A	MS cases	3	RS17129314	197 031 608	G	-0.12	7.50E-06
IE1A	MS cases	5	RS6894855	111 149 480	C	0.05547	5.38E-06
IE1A	MS cases	6	RS2247056	31 265 490	A	-0.04979	3.50E-06
IE1A	MS cases	6	RS2596487	31 325 056	A	-0.0665	2.24E-06
IE1A	MS cases	6	RS2523573	31 328 988	C	-0.07431	2.92E-06
IE1A	MS cases	6	RS9266669	31 348 077	A	-0.06755	6.81E-06
IE1A	MS cases	6	RS3094013	31 434 366	A	-0.07616	1.21E-06
IE1A	MS cases	6	RS3131618	31 434 621	G	-0.0751	1.90E-06
IE1A	MS cases	6	RS3131643	31 442 782	A	-0.06924	2.03E-06
IE1A	MS cases	6	RS3099844	31 448 976	A	-0.08391	8.61E-08
IE1A	MS cases	6	RS3094011	31 451 836	G	-0.08265	1.61E-07
IE1A	MS cases	6	RS9267445	31 483 481	C	-0.07734	1.20E-06
IE1A	MS cases	6	RS2734583	31 505 480	G	-0.07186	3.96E-06
IE1A	MS cases	6	RS3117582	31 620 520	C	-0.07401	4.49E-06
IE1A	MS cases	6	RS9267531	31 636 742	G	-0.07392	4.59E-06
IE1A	MS cases	6	**RS17201046**	31 699 573	A	0.1805	**9.19E-09**
IE1A	MS cases	6	RS3101018	31 705 864	A	-0.0748	4.29E-06
IE1A	MS cases	6	RS3132445	31 712 196	A	-0.07315	5.91E-06
IE1A	MS cases	6	RS3130484	31 715 882	G	-0.07357	5.17E-06
IE1A	MS cases	6	RS3131379	31 721 033	A	-0.07301	6.32E-06
IE1A	MS cases	6	RS3117574	31 725 230	A	-0.07258	6.87E-06
IE1A	MS cases	6	RS3131378	31 725 285	G	-0.07354	5.25E-06
IE1A	MS cases	6	RS3117577	31 727 474	G	-0.07395	4.65E-06
IE1A	MS cases	6	RS3101017	31 733 466	G	-0.07217	7.77E-06
IE1A	MS cases	6	RS2075798	31 846 741	A	0.088	4.54E-06
IE1A	MS cases	6	RS555007	31 850 332	G	0.08493	8.65E-06
IE1A	MS cases	6	RS558702	31 870 326	A	-0.07658	2.10E-06
IE1A	MS cases	6	RS519417	31 878 433	A	-0.07899	1.07E-06
IE1A	MS cases	6	RS497309	31 892 484	C	-0.07807	1.28E-06
IE1A	MS cases	6	RS1270942	31 918 860	G	-0.07857	1.09E-06
IE1A	MS cases	6	RS17201466	31 926 600	A	0.1494	1.80E-07
IE1A	MS cases	6	**RS6455**	32 006 896	G	0.1939	**4.35E-09**
IE1A	MS cases	6	**RS11969759**	32 021 130	A	0.1332	**1.44E-08**
IE1A	MS cases	6	RS1150755	32 038 550	A	-0.06753	2.02E-06
IE1A	MS cases	6	**RS7774197**	32 046 275	C	0.136	**6.67E-09**
IE1A	MS cases	6	RS1150754	32 050 758	A	-0.06824	1.51E-06
IE1A	MS cases	6	RS1150753	32 059 867	G	-0.07879	1.10E-06

IE1A	MS cases	6	RS1150752	32 064 726	G	-0.0783	1.47E-06
IE1A	MS cases	6	RS1269852	32 080 191	C	-0.07622	2.60E-06
IE1A	MS cases	6	RS3130288	32 096 001	A	-0.07667	2.13E-06
IE1A	MS cases	6	RS3134942	32 168 771	A	-0.07209	3.73E-06
IE1A	MS cases	6	RS3131296	32 172 993	A	-0.07384	2.43E-06
IE1A	MS cases	6	RS206018	32 177 880	C	0.06652	1.35E-06
IE1A	MS cases	6	RS3132956	32 179 438	A	-0.07153	4.50E-06
IE1A	MS cases	6	RS3131290	32 183 175	A	-0.05254	2.57E-06
IE1A	MS cases	6	RS915894	32 190 390	C	-0.05629	8.88E-07
IE1A	MS cases	6	RS6901158	32 205 942	A	-0.07489	7.00E-07
IE1A	MS cases	6	RS28895018	32 224 378	G	0.1245	3.11E-07
IE1A	MS cases	6	RS3132971	32 230 256	C	-0.0781	1.48E-06
IE1A	MS cases	6	RS7775397	32 261 252	C	-0.07617	2.54E-06
IE1A	MS cases	6	RS6906662	32 266 506	A	0.1231	4.03E-07
IE1A	MS cases	6	RS9268219	32 284 108	C	-0.07356	9.65E-06
IE1A	MS cases	6	RS9268235	32 290 208	A	-0.07543	3.27E-06
IE1A	MS cases	6	RS2073045	32 339 548	A	-0.05299	3.27E-06
IE1A	MS cases	6	**RS7758128**	32 345 283	A	0.1643	**2.40E-08**
IE1A	MS cases	6	RS2894254	32 345 689	C	-0.07478	4.16E-06
IE1A	MS cases	6	**RS17423649**	32 357 133	A	0.0891	**9.21E-09**
IE1A	MS cases	6	**RS17202259**	32 357 489	C	0.08966	**8.19E-09**
IE1A	MS cases	6	**RS16870123**	32 359 460	A	0.08766	**1.47E-08**
IE1A	MS cases	6	RS3817969	32 361 388	A	0.08306	6.62E-08
IE1A	MS cases	6	**RS3763326**	32 413 557	G	0.1593	**3.18E-08**
IE1A	MS cases	6	RS9272219	32 602 269	A	-0.05687	2.64E-06
IE1A	MS cases	6	RS9273012	32 611 641	G	-0.05651	3.06E-06
IE1A	MS cases	6	RS17843604	32 620 283	A	-0.05423	1.04E-06
IE1A	MS cases	6	**RS9273349**	32 625 869	G	-0.06591	**2.68E-09**
IE1A	MS cases	6	RS9273363	32 626 272	A	-0.05966	3.54E-07
IE1A	MS cases	6	RS6906021	32 626 311	G	-0.05084	8.96E-06
IE1A	MS cases	6	**RS1063355**	32 627 714	C	-0.06631	**2.18E-09**
IE1A	MS cases	6	RS28986337	32 748 398	A	0.089	4.20E-06
IE1A	MS cases	6	RS28986338	32 748 810	A	0.09066	2.71E-06
IE1A	MS cases	6	RS28986359	32 751 349	C	0.08728	6.28E-06
IE1A	MS cases	6	RS28986364	32 751 691	C	0.09062	3.08E-06
IE1A	MS cases	6	RS28986373	32 752 150	A	0.08992	3.23E-06
IE1A	MS cases	6	RS13204139	32 752 685	A	0.08955	3.93E-06
IE1A	MS cases	6	RS28986396	32 754 430	A	0.08915	4.03E-06
IE1A	MS cases	6	RS9276722	32 762 391	G	0.0887	4.32E-06
IE1A	MS cases	6	RS11244	32 780 724	A	-0.05508	4.62E-06
IE1A	MS cases	6	RS2621321	32 789 480	G	0.06177	1.21E-06

IE1A	MS cases	6	RS13209654	32 792 659	G	0.08885	4.18E-06
IE1A	MS cases	6	RS2857101	32 794 676	G	0.06138	1.44E-06
IE1A	MS cases	6	RS241456	32 795 965	A	0.05971	2.61E-06
IE1A	MS cases	6	RS241454	32 796 144	G	0.0608	1.66E-06
IE1A	MS cases	6	RS241453	32 796 226	A	0.06025	2.07E-06
IE1A	MS cases	6	RS241448	32 796 685	G	0.0588	3.67E-06
IE1A	MS cases	6	RS241447	32 796 751	G	0.06057	1.82E-06
IE1A	MS cases	6	RS241446	32 796 967	A	0.06009	2.29E-06
IE1A	MS cases	6	RS241445	32 797 072	A	0.06043	1.95E-06
IE1A	MS cases	6	RS241441	32 797 297	G	0.06057	1.86E-06
IE1A	MS cases	6	RS241440	32 797 361	A	0.06005	2.34E-06
IE1A	MS cases	6	RS3819714	32 804 217	A	0.05551	5.45E-07
IE1A	MS cases	6	RS3819715	32 804 219	A	0.0549	7.18E-07
IE1A	MS cases	6	RS3819720	32 804 570	A	-0.06002	1.35E-07
IE1A	MS cases	6	RS241425	32 804 909	G	-0.0526	5.28E-07
IE1A	MS cases	6	RS241424	32 804 934	A	-0.05336	4.68E-07
IE1A	MS cases	6	RS2239701	32 805 049	G	-0.04773	8.04E-06
IE1A	MS cases	6	RS2071538	32 818 678	A	0.06102	3.64E-07
IE1A	MS cases	6	RS1383266	32 834 732	A	0.05743	9.65E-07
IE1A	MS cases	6	**RS423196**	33 003 562	G	-0.08783	**3.55E-09**
IE1A	MS cases	6	**RS406477**	33 005 644	G	-0.08903	**1.65E-09**
IE1A	MS cases	6	RS2395309	33 026 246	G	-0.07036	2.65E-06
IE1A	MS cases	6	RS17214519	33 032 188	A	-0.07162	1.91E-06
IE1A	MS cases	6	RS7905	33 032 975	G	-0.08074	9.06E-07
IE1A	MS cases	6	RS3077	33 033 022	G	-0.07039	2.66E-06
IE1A	MS cases	6	RS9469341	33 035 877	G	-0.07277	1.26E-06
IE1A	MS cases	6	RS1042190	33 036 999	G	-0.07034	2.72E-06
IE1A	MS cases	6	RS10214910	33 037 675	A	-0.07121	2.20E-06
IE1A	MS cases	6	RS2301224	33 038 369	A	-0.07074	2.49E-06
IE1A	MS cases	6	RS2301220	33 038 766	A	-0.07256	1.36E-06
IE1A	MS cases	6	RS6914849	33 040 715	A	-0.07198	1.61E-06
IE1A	MS cases	6	RS1431399	33 041 034	G	-0.07134	2.03E-06
IE1A	MS cases	6	RS1431400	33 041 176	A	-0.07156	1.86E-06
IE1A	MS cases	6	RS1431401	33 041 186	A	-0.07145	1.93E-06
IE1A	MS cases	6	**RS987870**	33 042 880	G	-0.09732	**2.24E-08**
IE1A	MS cases	6	RS2071351	33 043 930	G	-0.07137	2.02E-06
IE1A	MS cases	6	RS2071353	33 044 257	G	-0.08087	1.51E-07
IE1A	MS cases	6	**RS2071354**	33 044 388	G	-0.09685	**2.65E-08**
IE1A	MS cases	6	**RS1042151**	33 048 661	G	-0.08181	**2.65E-10**
IE1A	MS cases	6	**RS9277378**	33 050 279	G	-0.07791	**8.68E-12**
IE1A	MS cases	6	**RS9277386**	33 050 499	G	-0.08023	**2.20E-12**

IE1A	MS cases	6	**RS9277396**	33 051 139	A	-0.07419	**5.25E-11**
IE1A	MS cases	6	**RS9277426**	33 051 910	A	-0.07374	**6.61E-11**
IE1A	MS cases	6	**RS9277469**	33 053 468	A	-0.0746	**3.93E-11**
IE1A	MS cases	6	**RS9277471**	33 053 682	A	-0.07295	**1.07E-10**
IE1A	MS cases	6	**RS9277533**	33 054 721	A	-0.07373	**6.69E-11**
IE1A	MS cases	6	**RS9277546**	33 055 346	C	-0.07405	**5.54E-11**
IE1A	MS cases	6	**RS9277554**	33 055 538	A	-0.07386	**6.04E-11**
IE1A	MS cases	6	**RS9277555**	33 055 605	A	-0.07125	**4.87E-10**
IE1A	MS cases	6	**RS3117228**	33 056 435	A	-0.07389	**5.87E-11**
IE1A	MS cases	6	**RS3130188**	33 057 176	G	-0.07399	**5.72E-11**
IE1A	MS cases	6	**RS3091282**	33 057 198	C	-0.07446	**4.26E-11**
IE1A	MS cases	6	**RS3117226**	33 057 659	A	-0.07616	**4.67E-11**
IE1A	MS cases	6	**RS3117225**	33 057 711	A	-0.07456	**4.74E-11**
IE1A	MS cases	6	**RS3097652**	33 057 835	A	-0.07396	**6.00E-11**
IE1A	MS cases	6	**RS1367730**	33 058 114	A	-0.0721	**3.22E-10**
IE1A	MS cases	6	**RS3128972**	33 058 774	G	-0.07101	**5.77E-10**
IE1A	MS cases	6	**RS2179920**	33 058 874	A	-0.07765	**1.86E-10**
IE1A	MS cases	6	**RS2179919**	33 059 262	G	-0.0715	**4.33E-10**
IE1A	MS cases	6	**RS3128917**	33 059 996	C	-0.07178	**3.63E-10**
IE1A	MS cases	6	**RS3117222**	33 060 949	A	-0.07143	**4.56E-10**
IE1A	MS cases	6	**RS3130190**	33 061 690	G	-0.0708	**6.62E-10**
IE1A	MS cases	6	**RS3130191**	33 061 871	G	-0.07081	**6.87E-10**
IE1A	MS cases	6	RS6914616	33 063 592	A	-0.1296	5.52E-08
IE1A	MS cases	6	**RS3130198**	33 063 931	G	-0.07189	**3.30E-10**
IE1A	MS cases	6	**RS3117213**	33 064 605	A	-0.07187	**3.56E-10**
IE1A	MS cases	6	RS2179915	33 065 734	A	-0.13	5.22E-08
IE1A	MS cases	6	**RS2395319**	33 067 211	A	-0.07192	**3.44E-10**
IE1A	MS cases	6	**RS3117239**	33 071 777	G	-0.07716	**2.19E-10**
IE1A	MS cases	6	**RS2064479**	33 072 240	A	-0.06877	**2.49E-09**
IE1A	MS cases	6	**RS2064478**	33 072 266	A	-0.07746	**1.89E-10**
IE1A	MS cases	6	**RS2064476**	33 073 322	G	-0.07383	**6.05E-11**
IE1A	MS cases	6	**RS3117234**	33 073 984	G	-0.08027	**2.72E-11**
IE1A	MS cases	6	**RS3117231**	33 074 908	G	-0.07594	**4.85E-11**
IE1A	MS cases	6	**RS3117230**	33 075 635	G	-0.07713	**2.21E-10**
IE1A	MS cases	6	**RS3128930**	33 075 666	A	-0.07784	**7.12E-11**
IE1A	MS cases	6	RS6924545	33 077 607	A	-0.1282	7.98E-08
IE1A	MS cases	9	RS2578246	90 835 589	G	-0.06121	2.45E-06
IE1A	MS cases	9	RS893494	90 875 879	G	-0.05975	1.49E-06
IE1A	Controls	5	RS2304028	150 891 733	A	0.05312	1.63E-06
IE1A	Controls	5	RS12717853	150 894 920	A	0.05378	1.25E-06

IE1A	Controls	5	RS3822699	150 901 111	A	0.05304	1.77E-06
IE1A	Controls	5	RS2053028	150 901 613	A	0.05455	9.35E-07
IE1A	Controls	6	RS1633041	29 733 223	A	-0.05464	1.63E-06
IE1A	Controls	6	RS1737041	29 736 229	A	-0.05459	1.65E-06
IE1A	Controls	6	RS1737031	29 737 481	A	-0.0549	1.01E-06
IE1A	Controls	6	RS1615962	29 745 726	A	-0.05446	1.88E-06
IE1A	Controls	6	RS1633030	29 745 794	G	-0.05497	9.69E-07
IE1A	Controls	6	RS1633028	29 746 367	C	-0.05477	1.09E-06
IE1A	Controls	6	RS2735042	29 747 373	G	-0.05473	1.07E-06
IE1A	Controls	6	RS1002046	29 754 016	A	-0.05449	1.74E-06
IE1A	Controls	6	RS1610644	29 759 445	A	-0.05421	1.97E-06
IE1A	Controls	6	RS1611205	29 759 823	G	-0.04935	7.90E-06
IE1A	Controls	6	RS1610723	29 761 473	C	-0.04881	9.45E-06
IE1A	Controls	6	RS1633011	29 762 693	A	-0.05455	1.84E-06
IE1A	Controls	6	RS1630969	29 765 001	A	-0.05697	9.53E-07
IE1A	Controls	6	RS1610649	29 768 917	G	-0.05527	3.41E-07
IE1A	Controls	6	RS1610711	29 769 497	C	-0.05469	1.65E-06
IE1A	Controls	6	RS1632988	29 772 395	A	-0.05667	1.24E-06
IE1A	Controls	6	RS1632987	29 772 549	G	-0.05521	1.31E-06
IE1A	Controls	6	RS1632983	29 773 509	A	-0.0543	1.11E-06
IE1A	Controls	6	RS1736971	29 776 322	A	-0.05446	1.78E-06
IE1A	Controls	6	RS1736969	29 776 390	A	-0.05538	1.22E-06
IE1A	Controls	6	RS1736959	29 782 470	A	-0.05341	3.49E-06
IE1A	Controls	6	RS1736957	29 782 633	G	-0.05266	2.71E-06
IE1A	Controls	6	RS1077432	29 782 868	G	-0.05392	2.27E-06
IE1A	Controls	6	RS1620173	29 785 149	C	-0.05463	1.65E-06
IE1A	Controls	6	RS1619379	29 785 235	A	-0.04883	3.89E-06
IE1A	Controls	6	RS1610675	29 788 606	A	-0.05209	3.64E-06
IE1A	Controls	6	**RS1610678**	29 789 190	G	-0.05937	**6.06E-09**
IE1A	Controls	6	**RS1611149**	29 789 999	A	-0.05959	**5.15E-09**
IE1A	Controls	6	**RS1736936**	29 794 317	A	-0.06359	**5.38E-10**
IE1A	Controls	6	**RS915669**	29 798 425	A	-0.06098	**2.20E-09**
IE1A	Controls	6	RS1611133	29 809 382	A	-0.06603	1.76E-07
IE1A	Controls	6	RS1611750	29 814 778	C	-0.06792	7.33E-08
IE1A	Controls	6	RS2734985	29 818 662	G	-0.05673	8.79E-06
IE1A	Controls	6	RS2247504	29 819 077	A	-0.05006	1.71E-06
IE1A	Controls	6	RS2523756	29 820 717	G	-0.05078	1.11E-06
IE1A	Controls	6	RS2428510	29 823 027	A	-0.05055	1.23E-06
IE1A	Controls	6	RS2517854	29 823 296	G	-0.0515	8.21E-07
IE1A	Controls	6	RS2844826	29 823 319	G	-0.05076	1.10E-06
IE1A	Controls	6	RS6919513	29 823 994	A	-0.05086	1.07E-06

IE1A	Controls	6	RS1611684	29 825 846	A	-0.05076	1.10E-06
IE1A	Controls	6	RS1611717	29 829 577	G	-0.05063	1.19E-06
IE1A	Controls	6	RS1611732	29 831 008	A	-0.05099	9.90E-07
IE1A	Controls	6	RS765649	29 831 058	A	-0.05003	1.69E-06
IE1A	Controls	6	RS1611737	29 831 571	G	-0.05309	3.55E-07
IE1A	Controls	6	RS1362104	30 101 656	A	-0.0514	7.91E-07
IE1A	Controls	6	RS3130557	31 094 703	A	-0.06998	8.09E-06
IE1A	Controls	6	RS3132510	31 172 151	G	-0.07342	6.09E-06
IE1A	Controls	6	RS9263911	31 175 667	G	0.04637	4.69E-06
IE1A	Controls	6	RS2844603	31 250 854	A	-0.04853	3.88E-06
IE1A	Controls	6	RS2853933	31 254 088	A	-0.04808	4.66E-06
IE1A	Controls	6	RS2524040	31 257 625	A	-0.04897	3.13E-06
IE1A	Controls	6	RS2524163	31 259 579	G	-0.04794	5.02E-06
IE1A	Controls	6	RS2524156	31 260 397	A	-0.04751	6.22E-06
IE1A	Controls	6	RS2243868	31 261 276	A	-0.04812	4.73E-06
IE1A	Controls	6	RS2247056	31 265 490	A	-0.05518	9.07E-07
IE1A	Controls	6	RS2853922	31 266 190	A	-0.04879	3.52E-06
IE1A	Controls	6	RS2524089	31 266 522	C	-0.04907	3.04E-06
IE1A	Controls	6	RS2524066	31 269 154	A	-0.0488	3.35E-06
IE1A	Controls	6	RS2523589	31 327 334	C	-0.04523	5.55E-06
IE1A	Controls	6	RS2844575	31 334 945	G	0.05234	2.13E-07
IE1A	Controls	6	RS9266669	31 348 077	A	-0.06755	4.06E-06
IE1A	Controls	6	RS3131618	31 434 621	G	-0.07055	9.24E-06
IE1A	Controls	6	RS3099844	31 448 976	A	-0.07435	2.02E-06
IE1A	Controls	6	RS3094011	31 451 836	G	-0.07683	9.80E-07
IE1A	Controls	6	RS9267445	31 483 481	C	-0.0729	4.42E-06
IE1A	Controls	6	RS2734583	31 505 480	G	-0.0715	4.51E-06
IE1A	Controls	6	**RS805262**	31 628 733	A	-0.05813	**1.26E-08**
IE1A	Controls	6	RS511294	31 888 869	C	0.1009	4.14E-06
IE1A	Controls	6	RS498240	31 892 592	A	0.102	3.83E-06
IE1A	Controls	6	RS2257331	31 898 285	G	0.1011	4.35E-06
IE1A	Controls	6	RS9332735	31 901 773	A	0.1278	4.53E-06
IE1A	Controls	6	RS635348	31 902 289	C	0.09978	6.23E-06
IE1A	Controls	6	RS621701	31 903 121	A	0.09823	8.52E-06
IE1A	Controls	6	**RS1042663**	31 905 130	A	0.1002	**4.90E-08**
IE1A	Controls	6	**RS2507953**	31 905 328	A	0.1003	**4.70E-08**
IE1A	Controls	6	RS550605	31 907 147	G	0.09945	6.21E-08
IE1A	Controls	6	**RS653414**	31 907 168	A	0.1003	**4.70E-08**
IE1A	Controls	6	**RS497239**	31 908 761	G	0.1003	**4.70E-08**
IE1A	Controls	6	RS609061	31 910 162	A	0.09993	5.06E-08
IE1A	Controls	6	**RS2242572**	31 910 929	A	0.1004	**4.28E-08**

IE1A	Controls	6	**RS547154**	31 910 938	A	0.1003	**4.53E-08**
IE1A	Controls	6	**RS541862**	31 916 951	G	0.1009	**3.92E-08**
IE1A	Controls	6	RS522162	31 919 917	G	0.1	5.11E-08
IE1A	Controls	6	**RS760070**	31 919 956	G	0.1006	**4.31E-08**
IE1A	Controls	6	**RS550513**	31 920 687	A	0.1006	**4.35E-08**
IE1A	Controls	6	**RS403569**	31 924 880	A	0.1009	**4.00E-08**
IE1A	Controls	6	**RS438999**	31 928 306	G	0.1001	**4.61E-08**
IE1A	Controls	6	RS444921	31 932 177	A	0.07361	6.04E-06
IE1A	Controls	6	RS406936	31 933 161	A	0.07374	5.79E-06
IE1A	Controls	6	RS454212	31 934 372	A	0.07354	6.22E-06
IE1A	Controls	6	RS453821	31 935 311	A	0.07322	6.80E-06
IE1A	Controls	6	RS449643	31 936 679	A	0.07368	5.91E-06
IE1A	Controls	6	RS387608	31 941 557	A	0.07303	6.95E-06
IE1A	Controls	6	RS12333245	32 019 769	A	0.08628	9.04E-07
IE1A	Controls	6	RS2269429	32 029 183	A	0.08671	8.64E-07
IE1A	Controls	6	RS1150755	32 038 550	A	-0.06335	6.31E-06
IE1A	Controls	6	RS1150754	32 050 758	A	-0.06325	6.50E-06
IE1A	Controls	6	RS169496	32 052 983	A	0.08675	7.84E-07
IE1A	Controls	6	RS204900	32 056 580	C	0.08665	8.05E-07
IE1A	Controls	6	RS204899	32 057 627	A	0.08686	8.33E-07
IE1A	Controls	6	RS204896	32 064 098	A	0.08419	1.84E-06
IE1A	Controls	6	RS439844	32 072 940	A	0.09022	3.11E-07
IE1A	Controls	6	**RS411337**	32 077 380	A	0.1069	**8.59E-09**
IE1A	Controls	6	**RS9469084**	32 080 383	A	0.1041	**2.11E-08**
IE1A	Controls	6	**RS204888**	32 089 142	A	0.1039	**2.22E-08**
IE1A	Controls	6	**RS169494**	32 097 876	A	0.1057	**1.31E-08**
IE1A	Controls	6	RS2071287	32 170 433	A	-0.04708	3.46E-06
IE1A	Controls	6	RS2071277	32 171 683	G	-0.04538	6.59E-06
IE1A	Controls	6	RS206017	32 176 211	G	0.04839	2.13E-06
IE1A	Controls	6	RS206018	32 177 880	C	0.06599	2.34E-07
IE1A	Controls	6	RS3131290	32 183 175	A	-0.05024	1.33E-06
IE1A	Controls	6	RS3134799	32 184 221	A	-0.04906	1.56E-06
IE1A	Controls	6	RS436388	32 186 264	A	0.04518	9.96E-06
IE1A	Controls	6	RS379464	32 186 348	A	0.1063	3.17E-06
IE1A	Controls	6	RS394657	32 187 023	G	-0.04698	3.96E-06
IE1A	Controls	6	RS8192584	32 189 192	A	0.122	2.76E-06
IE1A	Controls	6	RS915894	32 190 390	C	-0.05219	8.86E-07
IE1A	Controls	6	RS3132971	32 230 256	C	-0.07087	9.26E-06
IE1A	Controls	6	**RS17843604**	32 620 283	G	0.0553	**4.80E-08**
IE1A	Controls	6	**RS9273349**	32 625 869	A	0.06037	**2.41E-09**
IE1A	Controls	6	**RS1063355**	32 627 714	A	0.06106	**1.74E-09**

IE1A	Controls	12	RS4765808	5 319 541	A	-0.05014	8.44E-06
IE1A	Controls	16	RS277903	25 175 122	G	-0.05188	9.86E-06
IE1A	All subjects	0	RS3997868	0	G	-0.04484	5.94E-07
IE1A	All subjects	0	**RS602875**	0	G	-0.05105	**1.71E-08**
IE1A	All subjects	1	RS4659411	26 516 845	G	-0.03239	8.87E-06
IE1A	All subjects	3	RS433317	28 060 456	A	0.03335	5.30E-06
IE1A	All subjects	5	RS6894855	111 149 480	C	0.04042	2.25E-06
IE1A	All subjects	5	RS7718446	145 749 535	G	0.03621	5.03E-06
IE1A	All subjects	6	RS444189	29 605 935	C	-0.03411	6.22E-06
IE1A	All subjects	6	RS396660	29 646 165	A	-0.03648	3.82E-06
IE1A	All subjects	6	RS445150	29 646 879	G	-0.03616	4.60E-06
IE1A	All subjects	6	RS2747430	29 648 506	A	-0.03657	3.83E-06
IE1A	All subjects	6	RS1610736	29 718 544	G	0.03287	6.52E-06
IE1A	All subjects	6	RS909728	29 719 561	A	0.03303	5.90E-06
IE1A	All subjects	6	RS1633041	29 733 223	A	-0.03909	1.17E-06
IE1A	All subjects	6	RS2735048	29 733 701	A	0.03855	6.02E-07
IE1A	All subjects	6	RS2735046	29 734 098	A	0.03629	1.60E-06
IE1A	All subjects	6	RS1737041	29 736 229	A	-0.0391	1.16E-06
IE1A	All subjects	6	RS1737031	29 737 481	A	-0.03933	7.41E-07
IE1A	All subjects	6	RS1362068	29 742 108	G	-0.03777	1.04E-06
IE1A	All subjects	6	RS1362070	29 742 299	G	-0.03826	7.47E-07
IE1A	All subjects	6	RS1615962	29 745 726	A	-0.03845	1.81E-06
IE1A	All subjects	6	RS1633030	29 745 794	G	-0.03939	7.07E-07
IE1A	All subjects	6	RS1633028	29 746 367	C	-0.03932	7.51E-07
IE1A	All subjects	6	RS2735042	29 747 373	G	-0.03923	7.80E-07
IE1A	All subjects	6	RS1002046	29 754 016	A	-0.0391	1.17E-06
IE1A	All subjects	6	RS1610641	29 759 066	G	-0.03801	9.97E-07
IE1A	All subjects	6	RS1610644	29 759 445	A	-0.03918	1.10E-06
IE1A	All subjects	6	RS1611205	29 759 823	G	-0.03903	5.49E-07
IE1A	All subjects	6	RS1610719	29 760 677	G	-0.03823	8.49E-07
IE1A	All subjects	6	RS1610723	29 761 473	C	-0.03918	4.53E-07
IE1A	All subjects	6	RS1610724	29 761 516	G	-0.0386	6.65E-07
IE1A	All subjects	6	RS1611220	29 761 835	G	-0.03776	1.04E-06
IE1A	All subjects	6	RS1633011	29 762 693	A	-0.03889	1.39E-06
IE1A	All subjects	6	RS1633005	29 764 472	A	-0.04716	1.40E-06
IE1A	All subjects	6	RS1633003	29 764 759	G	-0.03733	1.37E-06
IE1A	All subjects	6	RS1630969	29 765 001	A	-0.03905	1.90E-06
IE1A	All subjects	6	**RS1610649**	29 768 917	G	-0.04533	**4.04E-09**
IE1A	All subjects	6	RS1610711	29 769 497	C	-0.03927	1.06E-06
IE1A	All subjects	6	RS1632995	29 769 621	T	-0.03717	1.61E-06

IE1A	All subjects	6	RS1610657	29 771 066	G	-0.03771	1.07E-06
IE1A	All subjects	6	RS1611192	29 771 879	G	-0.04067	2.49E-06
IE1A	All subjects	6	RS1632988	29 772 395	A	-0.03931	1.86E-06
IE1A	All subjects	6	RS1632987	29 772 549	G	-0.03894	1.30E-06
IE1A	All subjects	6	RS1632983	29 773 509	A	-0.03864	1.02E-06
IE1A	All subjects	6	RS1736976	29 773 999	G	-0.03773	1.19E-06
IE1A	All subjects	6	RS2523409	29 775 662	G	0.0362	2.13E-06
IE1A	All subjects	6	RS1736971	29 776 322	A	-0.03919	1.11E-06
IE1A	All subjects	6	RS1736969	29 776 390	A	-0.04005	6.56E-07
IE1A	All subjects	6	RS1610663	29 778 626	A	-0.03785	1.38E-06
IE1A	All subjects	6	RS1610699	29 778 987	C	-0.03696	2.22E-06
IE1A	All subjects	6	RS1610669	29 780 093	A	-0.03777	1.04E-06
IE1A	All subjects	6	RS1736959	29 782 470	A	-0.03742	4.21E-06
IE1A	All subjects	6	RS1736957	29 782 633	G	-0.03586	6.40E-06
IE1A	All subjects	6	RS1077432	29 782 868	G	-0.03939	1.02E-06
IE1A	All subjects	6	RS1620173	29 785 149	C	-0.03902	1.22E-06
IE1A	All subjects	6	**RS1619379**	29 785 235	A	-0.04206	**1.70E-08**
IE1A	All subjects	6	RS2735028	29 785 538	A	-0.03604	3.17E-06
IE1A	All subjects	6	RS1610675	29 788 606	A	-0.03697	3.14E-06
IE1A	All subjects	6	**RS1610678**	29 789 190	G	-0.05089	**2.39E-12**
IE1A	All subjects	6	**RS1611149**	29 789 999	A	-0.05145	**1.32E-12**
IE1A	All subjects	6	**RS1736936**	29 794 317	A	-0.05145	**1.79E-12**
IE1A	All subjects	6	**RS915669**	29 798 425	A	-0.05191	**8.45E-13**
IE1A	All subjects	6	RS1611133	29 809 382	A	-0.0438	6.19E-07
IE1A	All subjects	6	RS1611750	29 814 778	C	-0.04473	3.60E-07
IE1A	All subjects	6	RS2734985	29 818 662	G	-0.04604	2.24E-07
IE1A	All subjects	6	**RS2247504**	29 819 077	A	-0.04203	**1.40E-08**
IE1A	All subjects	6	RS2523759	29 819 093	G	-0.0448	4.25E-07
IE1A	All subjects	6	RS3115627	29 820 278	G	0.03638	2.90E-06
IE1A	All subjects	6	RS5013093	29 820 586	A	-0.04509	3.54E-07
IE1A	All subjects	6	**RS2523756**	29 820 717	G	-0.04332	**4.51E-09**
IE1A	All subjects	6	RS2734981	29 821 692	A	-0.04449	5.39E-07
IE1A	All subjects	6	RS2734980	29 821 896	A	-0.04509	3.58E-07
IE1A	All subjects	6	RS2517861	29 821 982	A	-0.04515	3.35E-07
IE1A	All subjects	6	RS2517860	29 822 521	G	-0.04592	2.10E-07
IE1A	All subjects	6	**RS2428510**	29 823 027	A	-0.04286	**6.65E-09**
IE1A	All subjects	6	**RS2517854**	29 823 296	G	-0.04405	**2.54E-09**
IE1A	All subjects	6	**RS2844826**	29 823 319	G	-0.04306	**5.48E-09**
IE1A	All subjects	6	**RS6919513**	29 823 994	A	-0.04305	**5.67E-09**
IE1A	All subjects	6	RS1611674	29 825 434	G	-0.03556	3.28E-06
IE1A	All subjects	6	**RS1611684**	29 825 846	A	-0.04309	**5.31E-09**

IE1A	All subjects	6	RS1611704	29 828 467	A	0.03688	2.21E-06
IE1A	All subjects	6	**RS1611717**	29 829 577	G	-0.04279	**6.85E-09**
IE1A	All subjects	6	**RS1611732**	29 831 008	A	-0.04301	**5.74E-09**
IE1A	All subjects	6	**RS765649**	29 831 058	A	-0.04262	**8.58E-09**
IE1A	All subjects	6	**RS1611737**	29 831 571	G	-0.04428	**2.05E-09**
IE1A	All subjects	6	RS3132682	30 044 388	C	0.03468	1.74E-06
IE1A	All subjects	6	RS7382061	30 047 965	A	0.03517	1.24E-06
IE1A	All subjects	6	RS2057728	30 054 757	A	0.03569	9.30E-07
IE1A	All subjects	6	RS6904455	30 055 377	G	0.03548	1.04E-06
IE1A	All subjects	6	RS9261394	30 064 562	A	0.03766	2.14E-07
IE1A	All subjects	6	RS1116222	30 071 279	C	-0.03957	2.11E-06
IE1A	All subjects	6	RS1116221	30 071 330	A	-0.03906	2.96E-06
IE1A	All subjects	6	RS1264341	30 802 465	G	-0.05024	6.89E-06
IE1A	All subjects	6	RS2535340	30 838 497	G	-0.05072	5.64E-06
IE1A	All subjects	6	RS1264322	30 857 894	A	-0.0502	7.16E-06
IE1A	All subjects	6	RS886422	30 864 279	A	-0.05024	6.86E-06
IE1A	All subjects	6	RS1049633	30 867 527	A	-0.05014	7.32E-06
IE1A	All subjects	6	RS1264312	30 872 982	A	-0.0501	8.17E-06
IE1A	All subjects	6	RS1264310	30 873 605	A	-0.04992	8.44E-06
IE1A	All subjects	6	RS1264308	30 879 987	A	-0.05026	6.77E-06
IE1A	All subjects	6	RS1264304	30 882 415	A	-0.05049	6.15E-06
IE1A	All subjects	6	RS3131921	30 907 335	G	-0.04759	7.38E-06
IE1A	All subjects	6	RS3094086	30 919 391	A	-0.04967	5.34E-06
IE1A	All subjects	6	RS3131934	30 931 844	G	-0.05249	6.09E-07
IE1A	All subjects	6	RS3131783	30 932 068	A	-0.0532	5.18E-07
IE1A	All subjects	6	RS1634721	30 977 680	A	-0.05657	4.16E-07
IE1A	All subjects	6	**RS3130544**	31 058 340	A	-0.06234	**1.76E-08**
IE1A	All subjects	6	RS2233974	31 080 016	C	-0.0488	6.24E-07
IE1A	All subjects	6	RS3823402	31 081 743	T	0.04574	5.57E-06
IE1A	All subjects	6	**RS3130557**	31 094 703	A	-0.06454	**5.43E-09**
IE1A	All subjects	6	**RS7750641**	31 129 310	A	-0.06444	**5.46E-09**
IE1A	All subjects	6	RS6921948	31 171 257	C	0.03708	2.92E-07
IE1A	All subjects	6	**RS3132510**	31 172 151	G	-0.0692	**1.83E-09**
IE1A	All subjects	6	RS2894181	31 174 527	G	0.03708	2.89E-07
IE1A	All subjects	6	RS9263911	31 175 667	G	0.03965	5.12E-08
IE1A	All subjects	6	RS3132505	31 177 503	A	-0.03891	9.28E-06
IE1A	All subjects	6	**RS3869109**	31 184 196	A	-0.04091	**2.23E-08**
IE1A	All subjects	6	RS9263964	31 186 039	G	-0.03983	5.11E-08
IE1A	All subjects	6	RS2394895	31 206 979	G	-0.03918	7.98E-06
IE1A	All subjects	6	RS3868082	31 207 692	A	-0.03914	1.05E-07
IE1A	All subjects	6	RS4084262	31 218 889	A	0.04689	2.70E-06

IE1A	All subjects	6	RS2394944	31 220 450	A	-0.04118	3.92E-06
IE1A	All subjects	6	RS1793891	31 221 698	A	-0.04086	2.18E-06
IE1A	All subjects	6	RS1986997	31 228 410	A	-0.03911	1.02E-07
IE1A	All subjects	6	RS2245822	31 230 800	A	-0.04147	2.07E-06
IE1A	All subjects	6	**RS1049281**	31 236 567	A	-0.04098	**3.84E-08**
IE1A	All subjects	6	RS2844613	31 243 846	A	-0.05071	5.59E-07
IE1A	All subjects	6	**RS2524074**	31 244 021	G	-0.04413	**5.88E-09**
IE1A	All subjects	6	**RS2844603**	31 250 854	A	-0.04116	**2.32E-08**
IE1A	All subjects	6	**RS2853933**	31 254 088	A	-0.04086	**2.77E-08**
IE1A	All subjects	6	**RS2524040**	31 257 625	A	-0.0417	**1.47E-08**
IE1A	All subjects	6	**RS2524163**	31 259 579	G	-0.04091	**2.69E-08**
IE1A	All subjects	6	**RS2524156**	31 260 397	A	-0.04072	**3.26E-08**
IE1A	All subjects	6	**RS2243868**	31 261 276	A	-0.04094	**2.68E-08**
IE1A	All subjects	6	RS2246954	31 265 262	A	-0.03549	1.15E-06
IE1A	All subjects	6	**RS2247056**	31 265 490	A	-0.05209	**2.00E-11**
IE1A	All subjects	6	**RS2853922**	31 266 190	A	-0.04197	**1.23E-08**
IE1A	All subjects	6	**RS2524089**	31 266 522	C	-0.04204	**1.12E-08**
IE1A	All subjects	6	**RS2524066**	31 269 154	A	-0.04197	**1.14E-08**
IE1A	All subjects	6	RS9264904	31 272 553	A	0.04298	1.39E-06
IE1A	All subjects	6	RS3094691	31 274 693	A	0.03823	4.21E-07
IE1A	All subjects	6	**RS2596487**	31 325 056	A	-0.06346	**1.76E-10**
IE1A	All subjects	6	RS2523589	31 327 334	A	0.03841	9.16E-08
IE1A	All subjects	6	**RS2523573**	31 328 988	C	-0.06997	**5.93E-10**
IE1A	All subjects	6	RS2844575	31 334 945	G	0.03776	2.16E-07
IE1A	All subjects	6	**RS9266669**	31 348 077	A	-0.06718	**1.82E-10**
IE1A	All subjects	6	**RS3094013**	31 434 366	A	-0.07145	**1.67E-10**
IE1A	All subjects	6	**RS3131618**	31 434 621	G	-0.07275	**9.38E-11**
IE1A	All subjects	6	RS2518028	31 436 047	A	-0.04051	5.95E-08
IE1A	All subjects	6	RS1055569	31 440 082	A	0.03638	7.49E-06
IE1A	All subjects	6	RS3828886	31 440 552	C	0.03735	9.62E-06
IE1A	All subjects	6	**RS3131643**	31 442 782	A	-0.06235	**7.85E-10**
IE1A	All subjects	6	**RS3099844**	31 448 976	A	-0.07919	**1.03E-12**
IE1A	All subjects	6	RS2523706	31 451 567	G	-0.03435	9.73E-06
IE1A	All subjects	6	**RS3094011**	31 451 836	G	-0.07964	**9.86E-13**
IE1A	All subjects	6	RS9267280	31 457 633	A	0.06073	6.24E-06
IE1A	All subjects	6	RS2855812	31 472 720	A	-0.04357	1.17E-07
IE1A	All subjects	6	**RS9267445**	31 483 481	C	-0.07497	**3.07E-11**
IE1A	All subjects	6	RS9469021	31 490 970	A	0.08315	4.33E-06
IE1A	All subjects	6	RS3093988	31 492 453	A	-0.04483	5.05E-06
IE1A	All subjects	6	RS2516482	31 496 569	G	-0.04383	7.24E-06
IE1A	All subjects	6	**RS2734583**	31 505 480	G	-0.07134	**1.10E-10**

IE1A	All subjects	6	RS3093542	31 540 693	G	0.08865	1.95E-06
IE1A	All subjects	6	RS1800629	31 543 031	A	-0.04322	9.40E-06
IE1A	All subjects	6	**RS3117582**	31 620 520	C	-0.07007	**8.78E-10**
IE1A	All subjects	6	**RS805262**	31 628 733	A	-0.05099	**5.15E-12**
IE1A	All subjects	6	RS707920	31 629 096	G	0.04234	6.22E-07
IE1A	All subjects	6	RS7029	31 629 953	G	0.04212	7.51E-07
IE1A	All subjects	6	RS7992	31 630 241	A	0.04249	5.81E-07
IE1A	All subjects	6	**RS9267531**	31 636 742	G	-0.06994	**9.58E-10**
IE1A	All subjects	6	RS805290	31 648 403	A	0.04324	3.68E-07
IE1A	All subjects	6	RS805282	31 659 731	C	0.04356	2.96E-07
IE1A	All subjects	6	RS805281	31 661 489	G	0.0432	3.74E-07
IE1A	All subjects	6	RS805277	31 662 957	A	0.04353	2.93E-07
IE1A	All subjects	6	RS805274	31 665 194	G	0.04293	4.41E-07
IE1A	All subjects	6	RS805287	31 678 730	G	0.03896	3.35E-06
IE1A	All subjects	6	**RS17201046**	31 699 573	A	0.115	**2.30E-08**
IE1A	All subjects	6	**RS3101018**	31 705 864	A	-0.07111	**6.34E-10**
IE1A	All subjects	6	**RS3132445**	31 712 196	A	-0.06921	**1.43E-09**
IE1A	All subjects	6	**RS3130484**	31 715 882	G	-0.06951	**1.19E-09**
IE1A	All subjects	6	**RS3131379**	31 721 033	A	-0.06897	**1.71E-09**
IE1A	All subjects	6	**RS3117574**	31 725 230	A	-0.06895	**1.61E-09**
IE1A	All subjects	6	**RS3131378**	31 725 285	G	-0.06967	**1.11E-09**
IE1A	All subjects	6	**RS3117577**	31 727 474	G	-0.0698	**1.03E-09**
IE1A	All subjects	6	**RS3101017**	31 733 466	G	-0.06891	**1.69E-09**
IE1A	All subjects	6	RS707926	31 748 820	A	0.04336	3.78E-06
IE1A	All subjects	6	RS707924	31 751 727	A	0.04265	5.44E-06
IE1A	All subjects	6	RS480092	31 764 899	G	0.04337	3.72E-06
IE1A	All subjects	6	**RS2075798**	31 846 741	A	0.07719	**4.87E-09**
IE1A	All subjects	6	**RS555007**	31 850 332	G	0.07459	**1.42E-08**
IE1A	All subjects	6	RS652888	31 851 234	G	-0.04254	2.92E-06
IE1A	All subjects	6	**RS558702**	31 870 326	A	-0.07072	**6.01E-10**
IE1A	All subjects	6	**RS519417**	31 878 433	A	-0.07196	**3.21E-10**
IE1A	All subjects	6	**RS511294**	31 888 869	C	0.08785	**4.80E-08**
IE1A	All subjects	6	**RS497309**	31 892 484	C	-0.07158	**3.64E-10**
IE1A	All subjects	6	**RS498240**	31 892 592	A	0.08946	**3.36E-08**
IE1A	All subjects	6	**RS2257331**	31 898 285	G	0.08886	**3.92E-08**
IE1A	All subjects	6	**RS9332735**	31 901 773	A	0.1145	**4.64E-08**
IE1A	All subjects	6	RS635348	31 902 289	C	0.08785	6.21E-08
IE1A	All subjects	6	RS621701	31 903 121	A	0.08796	5.50E-08
IE1A	All subjects	6	**RS1042663**	31 905 130	A	0.089	**3.11E-11**
IE1A	All subjects	6	**RS2507953**	31 905 328	A	0.08916	**2.84E-11**
IE1A	All subjects	6	**RS550605**	31 907 147	G	0.08912	**2.93E-11**

IE1A	All subjects	6	**RS653414**	31 907 168	A	0.08911	**2.91E-11**
IE1A	All subjects	6	**RS497239**	31 908 761	G	0.08849	**3.86E-11**
IE1A	All subjects	6	**RS609061**	31 910 162	A	0.08895	**2.96E-11**
IE1A	All subjects	6	**RS2242572**	31 910 929	A	0.08909	**2.73E-11**
IE1A	All subjects	6	**RS547154**	31 910 938	A	0.08901	**2.87E-11**
IE1A	All subjects	6	**RS541862**	31 916 951	G	0.08937	**2.51E-11**
IE1A	All subjects	6	**RS1270942**	31 918 860	G	-0.07234	**2.39E-10**
IE1A	All subjects	6	**RS522162**	31 919 917	G	0.08855	**3.77E-11**
IE1A	All subjects	6	**RS760070**	31 919 956	G	0.08896	**3.06E-11**
IE1A	All subjects	6	**RS550513**	31 920 687	A	0.08955	**2.38E-11**
IE1A	All subjects	6	**RS403569**	31 924 880	A	0.08959	**2.30E-11**
IE1A	All subjects	6	RS17201466	31 926 600	A	0.09887	1.42E-07
IE1A	All subjects	6	RS440454	31 927 342	A	-0.03654	1.40E-06
IE1A	All subjects	6	**RS438999**	31 928 306	G	0.08924	**2.30E-11**
IE1A	All subjects	6	RS419788	31 928 799	A	-0.03646	1.50E-06
IE1A	All subjects	6	RS437179	31 929 014	A	-0.03632	1.63E-06
IE1A	All subjects	6	RS429608	31 930 462	A	0.05766	7.06E-08
IE1A	All subjects	6	RS444921	31 932 177	A	0.05985	3.24E-07
IE1A	All subjects	6	RS406936	31 933 161	A	0.05999	3.06E-07
IE1A	All subjects	6	RS492899	31 933 518	G	0.06368	7.34E-06
IE1A	All subjects	6	RS454212	31 934 372	A	0.05991	3.18E-07
IE1A	All subjects	6	RS453821	31 935 311	A	0.05972	3.45E-07
IE1A	All subjects	6	RS410851	31 936 668	A	-0.03693	1.08E-06
IE1A	All subjects	6	RS449643	31 936 679	A	0.05989	3.18E-07
IE1A	All subjects	6	RS387608	31 941 557	A	0.05923	4.19E-07
IE1A	All subjects	6	RS389883	31 947 460	C	-0.0363	1.64E-06
IE1A	All subjects	6	**RS6455**	32 006 896	G	0.1333	**1.32E-09**
IE1A	All subjects	6	RS12333245	32 019 769	A	0.06075	1.37E-06
IE1A	All subjects	6	**RS11969759**	32 021 130	A	0.08565	**3.66E-08**
IE1A	All subjects	6	RS2269429	32 029 183	A	0.06028	1.77E-06
IE1A	All subjects	6	**RS1150755**	32 038 550	A	-0.06558	**6.14E-11**
IE1A	All subjects	6	**RS7774197**	32 046 275	C	0.08616	**2.88E-08**
IE1A	All subjects	6	**RS1150754**	32 050 758	A	-0.06593	**4.64E-11**
IE1A	All subjects	6	RS169496	32 052 983	A	0.06026	1.65E-06
IE1A	All subjects	6	RS204900	32 056 580	C	0.06043	1.55E-06
IE1A	All subjects	6	RS204899	32 057 627	A	0.0614	1.22E-06
IE1A	All subjects	6	**RS1150753**	32 059 867	G	-0.0717	**3.71E-10**
IE1A	All subjects	6	RS204896	32 064 098	A	0.06018	1.97E-06
IE1A	All subjects	6	**RS1150752**	32 064 726	G	-0.07329	**1.89E-10**
IE1A	All subjects	6	RS439844	32 072 940	A	0.06215	8.39E-07
IE1A	All subjects	6	**RS411337**	32 077 380	A	0.07306	**3.30E-08**

IE1A	All subjects	6	**RS1269852**	32 080 191	C	-0.06916	**1.53E-09**
IE1A	All subjects	6	RS9469084	32 080 383	A	0.07175	5.80E-08
IE1A	All subjects	6	RS204888	32 089 142	A	0.0716	6.20E-08
IE1A	All subjects	6	**RS3130288**	32 096 001	A	-0.06985	**9.75E-10**
IE1A	All subjects	6	**RS169494**	32 097 876	A	0.0736	**2.70E-08**
IE1A	All subjects	6	RS3134608	32 117 971	C	-0.04683	9.10E-07
IE1A	All subjects	6	RS2269425	32 123 639	A	0.05567	7.96E-08
IE1A	All subjects	6	RS3096697	32 134 510	A	-0.04712	8.05E-07
IE1A	All subjects	6	RS3130347	32 134 656	G	-0.04712	7.83E-07
IE1A	All subjects	6	RS3130284	32 140 487	G	-0.04722	7.67E-07
IE1A	All subjects	6	RS408359	32 141 883	A	0.07315	1.23E-06
IE1A	All subjects	6	RS3134947	32 145 205	A	-0.04722	7.47E-07
IE1A	All subjects	6	RS3134945	32 146 492	A	-0.047	8.47E-07
IE1A	All subjects	6	**RS3130349**	32 147 696	A	-0.05448	**3.74E-08**
IE1A	All subjects	6	**RS1800625**	32 152 442	G	-0.05439	**3.81E-08**
IE1A	All subjects	6	RS204995	32 154 285	G	-0.04329	4.31E-06
IE1A	All subjects	6	RS204993	32 155 581	G	-0.04492	4.18E-07
IE1A	All subjects	6	RS204992	32 156 908	A	-0.04296	6.08E-06
IE1A	All subjects	6	RS176095	32 158 319	G	-0.04249	8.16E-06
IE1A	All subjects	6	RS204991	32 161 366	G	-0.04803	1.36E-06
IE1A	All subjects	6	RS204990	32 161 430	A	-0.04918	9.34E-07
IE1A	All subjects	6	**RS2071278**	32 165 444	G	-0.06063	**8.32E-09**
IE1A	All subjects	6	**RS3134942**	32 168 771	A	-0.06875	**3.04E-10**
IE1A	All subjects	6	**RS2071287**	32 170 433	A	-0.04163	**2.17E-08**
IE1A	All subjects	6	RS3132935	32 171 075	G	-0.04722	8.87E-07
IE1A	All subjects	6	**RS2071277**	32 171 683	G	-0.04267	**8.14E-09**
IE1A	All subjects	6	**RS3131296**	32 172 993	A	-0.06988	**1.89E-10**
IE1A	All subjects	6	**RS206017**	32 176 211	G	0.04042	**4.97E-08**
IE1A	All subjects	6	**RS206018**	32 177 880	C	0.06657	**1.62E-12**
IE1A	All subjects	6	**RS3132956**	32 179 438	A	-0.06847	**3.64E-10**
IE1A	All subjects	6	**RS3131290**	32 183 175	A	-0.05167	**1.52E-11**
IE1A	All subjects	6	**RS3134799**	32 184 221	A	-0.04677	**4.86E-10**
IE1A	All subjects	6	RS436388	32 186 264	A	0.03911	1.40E-07
IE1A	All subjects	6	**RS379464**	32 186 348	A	0.1051	**5.96E-10**
IE1A	All subjects	6	**RS394657**	32 187 023	G	-0.0447	**2.46E-09**
IE1A	All subjects	6	RS1109771	32 187 605	G	-0.03547	1.65E-06
IE1A	All subjects	6	**RS8192584**	32 189 192	A	0.1223	**4.69E-10**
IE1A	All subjects	6	**RS915894**	32 190 390	C	-0.05418	**4.83E-12**
IE1A	All subjects	6	RS3134930	32 191 620	A	-0.04748	2.08E-07
IE1A	All subjects	6	RS483574	32 194 956	A	0.04739	6.44E-06
IE1A	All subjects	6	RS482759	32 195 017	G	0.04748	6.20E-06

IE1A	All subjects	6	RS377763	32 199 144	A	-0.04214	4.78E-06
IE1A	All subjects	6	**RS6901158**	32 205 942	A	-0.06911	**5.93E-11**
IE1A	All subjects	6	RS424232	32 208 324	A	-0.04258	9.21E-07
IE1A	All subjects	6	**RS28895018**	32 224 378	G	0.08694	**2.64E-08**
IE1A	All subjects	6	**RS3132971**	32 230 256	C	-0.07448	**7.29E-11**
IE1A	All subjects	6	**RS7775397**	32 261 252	C	-0.07297	**1.62E-10**
IE1A	All subjects	6	RS17422797	32 265 528	A	0.06858	7.21E-07
IE1A	All subjects	6	**RS6906662**	32 266 506	A	0.08641	**3.17E-08**
IE1A	All subjects	6	**RS9268219**	32 284 108	C	-0.06752	**7.72E-09**
IE1A	All subjects	6	**RS9268235**	32 290 208	A	-0.0728	**1.88E-10**
IE1A	All subjects	6	**RS2073045**	32 339 548	A	-0.04301	**3.97E-08**
IE1A	All subjects	6	**RS7758128**	32 345 283	A	0.1299	**1.27E-11**
IE1A	All subjects	6	**RS2894254**	32 345 689	C	-0.07211	**3.15E-10**
IE1A	All subjects	6	**RS17423649**	32 357 133	A	0.07176	**4.71E-12**
IE1A	All subjects	6	**RS17202259**	32 357 489	C	0.07204	**4.39E-12**
IE1A	All subjects	6	RS3117099	32 358 270	A	-0.03995	4.83E-06
IE1A	All subjects	6	**RS16870123**	32 359 460	A	0.07033	**1.07E-11**
IE1A	All subjects	6	**RS3817969**	32 361 388	A	0.06703	**7.61E-11**
IE1A	All subjects	6	RS4248166	32 366 421	G	0.04613	6.15E-07
IE1A	All subjects	6	RS2294884	32 367 259	C	0.04616	5.54E-07
IE1A	All subjects	6	RS2294882	32 367 515	G	0.04329	2.33E-06
IE1A	All subjects	6	RS2294881	32 367 604	G	0.04283	2.96E-06
IE1A	All subjects	6	RS3763304	32 369 355	A	0.04604	5.88E-07
IE1A	All subjects	6	**RS3129843**	32 395 726	G	-0.07057	**7.62E-10**
IE1A	All subjects	6	**RS3763326**	32 413 557	G	0.1189	**1.63E-10**
IE1A	All subjects	6	RS9268877	32 431 147	A	0.03788	4.28E-07
IE1A	All subjects	6	RS9268979	32 435 044	A	0.0381	4.15E-07
IE1A	All subjects	6	**RS9270665**	32 566 232	A	-0.05394	**5.96E-09**
IE1A	All subjects	6	RS660895	32 577 380	G	-0.04462	6.62E-07
IE1A	All subjects	6	**RS642093**	32 582 075	A	-0.05064	**2.36E-08**
IE1A	All subjects	6	RS17533090	32 590 722	A	0.04805	8.44E-06
IE1A	All subjects	6	**RS3129763**	32 590 925	A	-0.05371	**6.96E-09**
IE1A	All subjects	6	**RS9272219**	32 602 269	A	-0.05302	**2.81E-10**
IE1A	All subjects	6	RS2187668	32 605 884	A	-0.05422	7.17E-07
IE1A	All subjects	6	**RS9273012**	32 611 641	G	-0.05296	**2.93E-10**
IE1A	All subjects	6	**RS17843604**	32 620 283	A	-0.05466	**4.08E-13**
IE1A	All subjects	6	RS9273327	32 623 223	C	-0.05349	1.31E-06
IE1A	All subjects	6	**RS9273349**	32 625 869	G	-0.06273	**7.47E-17**
IE1A	All subjects	6	RS6928482	32 626 249	G	-0.0375	1.49E-06
IE1A	All subjects	6	**RS9273363**	32 626 272	A	-0.05312	**4.22E-11**
IE1A	All subjects	6	**RS6906021**	32 626 311	G	-0.04782	**8.20E-10**

IE1A	All subjects	6	**RS1063355**	32 627 714	C	-0.06328	**4.43E-17**
IE1A	All subjects	6	**RS9273448**	32 627 747	A	0.04561	**5.10E-09**
IE1A	All subjects	6	RS2854275	32 628 428	A	-0.05527	4.28E-07
IE1A	All subjects	6	RS9274741	32 637 994	G	-0.03668	1.90E-06
IE1A	All subjects	6	RS9275141	32 651 117	C	-0.04201	5.90E-08
IE1A	All subjects	6	RS2856695	32 651 894	G	-0.04158	8.16E-08
IE1A	All subjects	6	RS3021058	32 652 359	C	-0.04209	5.71E-08
IE1A	All subjects	6	RS4947342	32 653 070	A	-0.04391	6.27E-07
IE1A	All subjects	6	RS2856692	32 653 385	C	-0.0442	5.48E-07
IE1A	All subjects	6	**RS4642516**	32 657 543	A	-0.04238	**4.56E-08**
IE1A	All subjects	6	RS5000634	32 663 564	G	-0.04323	5.47E-08
IE1A	All subjects	6	**RS6457622**	32 664 163	C	-0.04395	**3.22E-08**
IE1A	All subjects	6	RS9275312	32 665 728	G	-0.04889	3.61E-07
IE1A	All subjects	6	**RS1794282**	32 666 526	A	-0.06743	**4.49E-09**
IE1A	All subjects	6	RS9275328	32 666 822	A	-0.0482	5.31E-07
IE1A	All subjects	6	RS9275330	32 666 875	G	-0.04836	4.91E-07
IE1A	All subjects	6	RS9275333	32 666 968	G	-0.04881	3.81E-07
IE1A	All subjects	6	**RS3135006**	32 667 119	A	0.04601	**4.10E-09**
IE1A	All subjects	6	RS4947344	32 677 846	A	0.03882	2.77E-07
IE1A	All subjects	6	RS9275601	32 682 664	A	-0.03613	1.79E-06
IE1A	All subjects	6	RS3873444	32 682 724	A	0.06443	3.65E-07
IE1A	All subjects	6	RS1383265	32 739 888	G	0.04684	2.89E-07
IE1A	All subjects	6	RS2857212	32 740 411	G	0.03882	3.48E-07
IE1A	All subjects	6	**RS28986337**	32 748 398	A	0.07994	**2.51E-09**
IE1A	All subjects	6	**RS28986338**	32 748 810	A	0.0806	**1.83E-09**
IE1A	All subjects	6	**RS28986359**	32 751 349	C	0.07823	**5.88E-09**
IE1A	All subjects	6	**RS28986364**	32 751 691	C	0.08022	**2.76E-09**
IE1A	All subjects	6	**RS9276689**	32 751 962	A	-0.06756	**1.94E-08**
IE1A	All subjects	6	**RS28986373**	32 752 150	A	0.08036	**2.02E-09**
IE1A	All subjects	6	**RS13204139**	32 752 685	A	0.0803	**2.33E-09**
IE1A	All subjects	6	**RS28986396**	32 754 430	A	0.07998	**2.47E-09**
IE1A	All subjects	6	**RS7762279**	32 755 290	G	-0.06748	**2.00E-08**
IE1A	All subjects	6	**RS9276722**	32 762 391	G	0.08029	**1.98E-09**
IE1A	All subjects	6	RS9276726	32 763 888	G	-0.0379	1.10E-06
IE1A	All subjects	6	RS1383261	32 765 451	A	-0.03421	9.71E-06
IE1A	All subjects	6	RS6932969	32 765 671	G	-0.03485	6.41E-06
IE1A	All subjects	6	RS7383606	32 766 593	A	-0.03468	7.03E-06
IE1A	All subjects	6	RS9276734	32 766 854	A	-0.03473	6.80E-06
IE1A	All subjects	6	RS7382714	32 767 496	G	-0.03473	6.92E-06
IE1A	All subjects	6	RS4947350	32 767 620	G	-0.0465	1.07E-07
IE1A	All subjects	6	RS7381376	32 767 673	G	-0.03525	5.21E-06

IE1A	All subjects	6	RS2621367	32 768 721	A	0.03929	6.70E-07
IE1A	All subjects	6	RS2199874	32 769 926	G	0.04007	3.52E-07
IE1A	All subjects	6	RS6899857	32 770 482	G	-0.03529	4.73E-06
IE1A	All subjects	6	RS2621358	32 770 808	A	0.03781	1.91E-06
IE1A	All subjects	6	RS6912414	32 774 465	A	-0.03771	1.19E-06
IE1A	All subjects	6	RS7382619	32 775 809	A	-0.03434	8.59E-06
IE1A	All subjects	6	RS7382649	32 776 087	A	-0.03463	7.26E-06
IE1A	All subjects	6	RS2621338	32 776 583	A	0.03788	1.82E-06
IE1A	All subjects	6	RS2857118	32 777 900	A	0.03787	2.04E-06
IE1A	All subjects	6	RS2857115	32 778 281	A	0.03964	9.59E-07
IE1A	All subjects	6	**RS11244**	32 780 724	A	-0.04657	**2.30E-08**
IE1A	All subjects	6	RS2071474	32 782 582	A	0.04235	1.74E-07
IE1A	All subjects	6	RS2071472	32 784 620	A	0.04238	1.77E-07
IE1A	All subjects	6	RS2071469	32 784 783	A	0.03576	2.17E-06
IE1A	All subjects	6	RS1894406	32 787 056	A	0.03687	1.89E-06
IE1A	All subjects	6	RS2621321	32 789 480	G	0.04648	7.14E-08
IE1A	All subjects	6	**RS13209654**	32 792 659	G	0.08061	**1.85E-09**
IE1A	All subjects	6	RS2857101	32 794 676	G	0.04603	9.78E-08
IE1A	All subjects	6	RS241456	32 795 965	A	0.04672	6.01E-08
IE1A	All subjects	6	**RS241454**	32 796 144	G	0.04715	**4.45E-08**
IE1A	All subjects	6	RS241453	32 796 226	A	0.04659	6.37E-08
IE1A	All subjects	6	RS241448	32 796 685	G	0.04623	8.30E-08
IE1A	All subjects	6	**RS241447**	32 796 751	G	0.04699	**4.92E-08**
IE1A	All subjects	6	RS241446	32 796 967	A	0.04668	6.35E-08
IE1A	All subjects	6	**RS241445**	32 797 072	A	0.04709	**4.61E-08**
IE1A	All subjects	6	RS241441	32 797 297	G	0.04664	6.31E-08
IE1A	All subjects	6	RS241440	32 797 361	A	0.04614	8.94E-08
IE1A	All subjects	6	**RS3819714**	32 804 217	A	0.04267	**4.91E-08**
IE1A	All subjects	6	RS3819715	32 804 219	A	0.0423	6.32E-08
IE1A	All subjects	6	**RS3819720**	32 804 570	A	-0.05035	**1.27E-10**
IE1A	All subjects	6	**RS241425**	32 804 909	A	0.04212	**9.94E-09**
IE1A	All subjects	6	RS241424	32 804 934	A	-0.03762	3.47E-07
IE1A	All subjects	6	RS2239701	32 805 049	G	-0.03998	7.53E-08
IE1A	All subjects	6	RS1480380	32 913 246	A	-0.06895	1.60E-07
IE1A	All subjects	6	RS9276933	32 930 795	G	-0.0659	1.90E-07
IE1A	All subjects	6	**RS423196**	33 003 562	G	-0.06196	**2.76E-09**
IE1A	All subjects	6	**RS406477**	33 005 644	G	-0.06265	**1.47E-09**
IE1A	All subjects	6	RS376877	33 024 606	C	-0.04424	6.42E-06
IE1A	All subjects	6	RS2395309	33 026 246	G	-0.05448	2.29E-07
IE1A	All subjects	6	RS17214519	33 032 188	A	-0.05521	1.68E-07
IE1A	All subjects	6	RS7905	33 032 975	G	-0.05866	3.10E-07

IE1A	All subjects	6	RS3077	33 033 022	G	-0.05424	2.58E-07
IE1A	All subjects	6	RS9469341	33 035 877	G	-0.05555	1.36E-07
IE1A	All subjects	6	RS1042190	33 036 999	G	-0.05423	2.61E-07
IE1A	All subjects	6	RS10214910	33 037 675	A	-0.05442	2.49E-07
IE1A	All subjects	6	RS2301224	33 038 369	A	-0.05416	2.67E-07
IE1A	All subjects	6	RS2301220	33 038 766	A	-0.05542	1.39E-07
IE1A	All subjects	6	RS6914849	33 040 715	A	-0.0552	1.55E-07
IE1A	All subjects	6	RS1431399	33 041 034	G	-0.05453	2.18E-07
IE1A	All subjects	6	RS1431400	33 041 176	A	-0.05487	1.81E-07
IE1A	All subjects	6	RS1431401	33 041 186	A	-0.05484	1.85E-07
IE1A	All subjects	6	**RS987870**	33 042 880	G	-0.07253	**1.82E-09**
IE1A	All subjects	6	RS2071351	33 043 930	G	-0.05449	2.30E-07
IE1A	All subjects	6	**RS2071353**	33 044 257	G	-0.06394	**3.28E-09**
IE1A	All subjects	6	**RS2071354**	33 044 388	G	-0.07234	**2.02E-09**
IE1A	All subjects	6	**RS1042151**	33 048 661	G	-0.05617	**1.15E-09**
IE1A	All subjects	6	**RS9277378**	33 050 279	G	-0.05042	**4.77E-10**
IE1A	All subjects	6	**RS9277386**	33 050 499	G	-0.05196	**1.41E-10**
IE1A	All subjects	6	**RS9277396**	33 051 139	A	-0.04625	**8.54E-09**
IE1A	All subjects	6	**RS9277426**	33 051 910	A	-0.04599	**1.02E-08**
IE1A	All subjects	6	**RS9277469**	33 053 468	A	-0.04682	**5.53E-09**
IE1A	All subjects	6	**RS9277471**	33 053 682	A	-0.04583	**1.20E-08**
IE1A	All subjects	6	**RS9277533**	33 054 721	A	-0.04582	**1.15E-08**
IE1A	All subjects	6	**RS9277546**	33 055 346	C	-0.04616	**9.20E-09**
IE1A	All subjects	6	**RS9277554**	33 055 538	A	-0.04606	**9.61E-09**
IE1A	All subjects	6	RS9277555	33 055 605	A	-0.04389	8.14E-08
IE1A	All subjects	6	**RS3117228**	33 056 435	A	-0.04612	**9.17E-09**
IE1A	All subjects	6	**RS3130188**	33 057 176	G	-0.04611	**9.38E-09**
IE1A	All subjects	6	**RS3091282**	33 057 198	C	-0.04649	**7.13E-09**
IE1A	All subjects	6	**RS3117226**	33 057 659	A	-0.0487	**3.75E-09**
IE1A	All subjects	6	**RS3117225**	33 057 711	A	-0.0464	**8.40E-09**
IE1A	All subjects	6	**RS3097652**	33 057 835	A	-0.04595	**1.09E-08**
IE1A	All subjects	6	RS1367730	33 058 114	A	-0.04427	6.53E-08
IE1A	All subjects	6	RS3128972	33 058 774	G	-0.04352	1.05E-07
IE1A	All subjects	6	**RS2179920**	33 058 874	A	-0.0487	**2.02E-08**
IE1A	All subjects	6	RS2179919	33 059 262	G	-0.04412	6.92E-08
IE1A	All subjects	6	RS3128917	33 059 996	C	-0.04435	5.91E-08
IE1A	All subjects	6	RS3117222	33 060 949	A	-0.04411	7.10E-08
IE1A	All subjects	6	RS3130190	33 061 690	G	-0.04365	9.67E-08
IE1A	All subjects	6	RS3130191	33 061 871	G	-0.04352	1.06E-07
IE1A	All subjects	6	**RS6914616**	33 063 592	A	-0.1102	**1.68E-11**
IE1A	All subjects	6	RS3130198	33 063 931	G	-0.04453	5.03E-08

IE1A	All subjects	6	**RS3117213**	33 064 605	A	-0.04466	**4.75E-08**
IE1A	All subjects	6	**RS2179915**	33 065 734	A	-0.1104	**1.58E-11**
IE1A	All subjects	6	**RS2395319**	33 067 211	A	-0.04497	**3.88E-08**
IE1A	All subjects	6	**RS3117239**	33 071 777	G	-0.04914	**1.38E-08**
IE1A	All subjects	6	RS2064479	33 072 240	A	-0.04345	1.33E-07
IE1A	All subjects	6	**RS2064478**	33 072 266	A	-0.04933	**1.23E-08**
IE1A	All subjects	6	**RS2064476**	33 073 322	G	-0.04644	**7.16E-09**
IE1A	All subjects	6	**RS3117234**	33 073 984	G	-0.05084	**3.19E-09**
IE1A	All subjects	6	**RS3117231**	33 074 908	G	-0.0492	**2.37E-09**
IE1A	All subjects	6	**RS3117230**	33 075 635	G	-0.04914	**1.38E-08**
IE1A	All subjects	6	**RS3128930**	33 075 666	A	-0.05013	**2.99E-09**
IE1A	All subjects	6	**RS6924545**	33 077 607	A	-0.1072	**5.64E-11**
IE1A	All subjects	6	RS6457713	33 077 776	A	-0.06478	2.11E-07
IE1A	All subjects	6	RS12529876	167 461 501	A	0.03555	1.61E-06
IE1A	All subjects	6	RS6905911	167 521 624	A	0.03366	4.54E-06
IE1A	All subjects	6	RS6907666	167 523 395	A	0.03363	6.16E-06
IE1A	All subjects	7	RS6461830	24 969 081	A	0.05543	9.77E-06
IE1A	All subjects	14	RS2582516	105 314 093	C	0.04642	1.24E-06
IE1A	All subjects	14	RS7157970	105 463 936	A	0.03492	8.32E-06
IE1A	All subjects	21	RS460014	16 091 441	A	0.03771	4.10E-06
IE1A	All subjects	22	RS2530682	30 103 476	A	-0.0342	8.89E-06
IE1B	MS cases	6	RS3016014	31 351 153	G	-0.05517	7.38E-07
IE1B	MS cases	6	RS2256175	31 380 449	A	0.05208	1.19E-06
IE1B	MS cases	6	RS10948679	52 012 873	G	-0.04987	1.87E-06
IE1B	MS cases	7	RS2598404	33 611 480	C	0.0523	4.91E-06
IE1B	MS cases	8	RS1829402	134 574 971	A	0.08937	8.01E-06
IE1B	MS cases	12	RS7958938	76 287 239	A	-0.05894	8.92E-06
IE1B	MS cases	13	RS1606713	61 581 129	A	-0.06691	3.33E-06
IE1B	MS cases	13	RS2323081	61 585 068	A	-0.06698	3.64E-06
IE1B	Controls	2	RS1374109	137 312 484	A	0.1229	4.09E-06
IE1B	Controls	5	RS10079074	111 102 229	A	0.05946	8.68E-06
IE1B	Controls	6	RS479536	32 193 678	A	0.1664	5.27E-07
IE1B	Controls	6	RS374205	32 196 873	G	0.1261	5.90E-06
IE1B	Controls	6	RS511027	32 206 687	A	0.09395	4.38E-06
IE1B	Controls	6	RS28366191	32 364 190	G	0.1588	2.15E-06
IE1B	Controls	6	RS6911777	32 409 996	G	0.1727	1.57E-07
IE1B	Controls	6	RS13204672	32 582 796	G	0.09352	5.24E-06
IE1B	Controls	6	RS2858308	32 670 000	A	0.1191	3.50E-06
IE1B	Controls	6	RS16898262	32 677 397	G	0.1198	3.14E-06

IE1B	Controls	6	RS2647089	32 681 568	G	0.06964	7.52E-06
IE1B	Controls	8	RS12056386	26 050 530	G	-0.07173	7.70E-06
IE1B	Controls	11	RS10832934	18 435 700	A	0.07591	7.10E-06
IE1B	All subjects	2	RS11896138	231 199 033	A	0.05443	7.84E-06
IE1B	All subjects	4	RS7667582	36 154 555	A	0.03636	5.18E-06
IE1B	All subjects	6	RS2394888	31 202 246	A	-0.03805	7.05E-06
IE1B	All subjects	6	RS3016014	31 351 153	G	-0.04297	3.79E-07
IE1B	All subjects	6	RS2256028	31 379 198	A	0.05436	7.24E-06
IE1B	All subjects	6	RS2516463	31 416 536	G	0.09309	4.51E-06
IE1B	All subjects	6	RS2596460	31 417 510	G	0.09267	4.79E-06
IE1B	All subjects	6	RS2596458	31 417 647	A	0.09257	4.82E-06
IE1B	All subjects	6	RS2516454	31 420 863	T	0.09579	2.44E-06
IE1B	All subjects	6	RS2395028	31 424 757	A	0.09285	4.79E-06
IE1B	All subjects	6	RS2596480	31 425 985	A	0.09373	3.77E-06
IE1B	All subjects	6	RS2534678	31 463 963	A	0.07393	1.12E-07
IE1B	All subjects	6	RS2534667	31 468 099	A	0.07811	1.52E-07
IE1B	All subjects	6	RS2534657	31 472 459	A	0.07356	1.45E-07
IE1B	All subjects	6	RS2844496	31 480 398	A	0.08718	7.98E-08
IE1B	All subjects	6	**RS2246986**	31 482 203	G	0.08893	**4.40E-08**
IE1B	All subjects	6	RS2844509	31 510 924	G	0.05425	6.26E-07
IE1B	All subjects	6	RS2239705	31 513 402	A	0.05972	1.38E-06
IE1B	All subjects	6	RS693797	32 204 433	G	0.03651	9.46E-06
IE1B	All subjects	6	**RS511027**	32 206 687	A	0.08366	**1.13E-08**
IE1B	All subjects	6	RS6910071	32 282 854	G	-0.04378	7.88E-06
IE1B	All subjects	6	RS9268557	32 389 305	G	-0.03555	8.62E-06
IE1B	All subjects	7	RS2598404	33 611 480	C	0.03855	7.35E-06
IE1B	All subjects	10	RS1334461	99 868 267	G	-0.03609	7.29E-06
IE1B	All subjects	10	RS1334465	99 876 121	G	-0.03667	5.21E-06
IE1B	All subjects	10	RS7911699	99 890 470	G	-0.03655	5.63E-06
IE1B	All subjects	10	RS7088359	99 891 010	G	-0.03598	7.56E-06
IE1B	All subjects	10	RS10748715	99 914 261	G	-0.03614	8.17E-06
IE1B	All subjects	10	RS10450402	99 990 493	G	-0.0376	3.41E-06
IE1B	All subjects	10	RS3793692	100 008 436	A	-0.03668	5.94E-06
IE1B	All subjects	12	RS7958938	76 287 239	A	-0.04589	3.82E-06
IE1B	All subjects	13	RS9576177	37 679 729	A	-0.03461	9.33E-06
IE1B	All subjects	13	RS11147624	37 679 937	A	-0.03463	8.36E-06
IE1B	All subjects	13	RS11147625	37 680 081	A	-0.03462	8.38E-06
101K	MS cases	1	RS12091891	9 000 220	G	-0.08138	7.38E-06
101K	MS cases	3	RS12495466	13 282 611	G	0.1228	1.54E-06

101K	MS cases	6	RS9275224	32 659 878	G	0.04213	4.55E-06
101K	MS cases	6	RS9275245	32 660 943	G	0.04119	7.95E-06
101K	MS cases	6	RS6457617	32 663 851	A	0.04363	2.00E-06
101K	MS cases	6	RS9275371	32 668 296	G	0.04765	2.26E-06
101K	MS cases	6	RS9275374	32 668 526	A	0.04759	2.26E-06
101K	MS cases	6	RS9275388	32 669 084	G	0.04726	2.69E-06
101K	MS cases	6	RS9275390	32 669 156	G	0.04814	1.81E-06
101K	MS cases	6	RS9275393	32 669 439	A	0.0473	2.85E-06
101K	MS cases	6	RS9275406	32 669 955	A	0.04729	2.70E-06
101K	MS cases	6	RS9275407	32 670 037	A	0.04783	2.02E-06
101K	MS cases	6	RS9275418	32 670 244	G	0.0475	2.37E-06
101K	MS cases	6	RS9275424	32 670 576	G	0.04749	2.41E-06
101K	MS cases	6	RS9275428	32 670 978	G	0.04675	3.74E-06
101K	MS cases	6	RS9275439	32 671 521	G	0.04799	2.06E-06
101K	MS cases	6	RS9275523	32 674 994	A	0.04874	6.97E-06
101K	MS cases	6	RS9275582	32 680 070	A	0.0506	3.17E-06
101K	MS cases	6	RS9275595	32 681 355	G	0.04939	5.48E-06
101K	MS cases	6	RS17335164	44 662 629	G	0.08871	2.56E-06
101K	MS cases	6	RS7762246	51 099 256	G	0.05713	7.15E-06
101K	MS cases	6	RS12210804	104 100 800	A	0.139	7.47E-06
101K	MS cases	7	RS6947751	18 025 423	G	-0.0418	2.16E-06
101K	MS cases	7	RS6947763	18 025 440	G	-0.0422	1.73E-06
101K	MS cases	9	RS2821545	15 564 904	A	-0.03938	7.51E-06
101K	MS cases	9	RS192466	15 585 395	G	-0.04013	4.80E-06
101K	MS cases	9	RS1355171	15 681 694	A	-0.03907	8.99E-06
101K	MS cases	9	RS10733295	15 681 901	G	-0.03898	9.05E-06
101K	MS cases	16	RS7194313	50 059 500	G	0.07224	1.50E-06
101K	MS cases	16	RS17213264	50 060 203	G	0.06841	5.00E-06
101K	MS cases	16	RS6500279	50 073 908	G	0.07042	6.74E-06
101K	MS cases	22	RS4823817	46 857 705	G	0.04215	6.70E-06

101K	Controls	3	RS7616406	12 862 257	G	0.04442	3.92E-06
101K	Controls	3	RS6442333	12 864 386	A	0.04729	1.31E-06
101K	Controls	3	RS7629133	12 866 725	G	0.04571	5.96E-06
101K	Controls	6	RS2844571	31 335 647	G	-0.04392	8.05E-06
101K	Controls	6	RS2253908	31 336 891	A	-0.0442	7.12E-06
101K	Controls	6	RS2596530	31 387 373	G	-0.04317	3.38E-06
101K	Controls	6	RS2596531	31 387 557	G	-0.04356	2.75E-06
101K	Controls	6	RS2844511	31 389 784	A	-0.04345	2.89E-06
101K	Controls	6	RS2516448	31 390 410	A	-0.04346	2.89E-06
101K	Controls	6	RS2269475	31 583 931	A	0.05551	9.37E-06

101K	Controls	6	RS3763295	31 587 938	G	0.05879	3.06E-06
101K	Controls	6	RS14365	31 635 710	G	0.05665	2.09E-06
101K	Controls	6	RS28421666	32 592 737	G	0.09872	6.31E-06
101K	Controls	6	RS7774434	32 657 578	G	0.04619	1.27E-06
101K	Controls	6	RS6931062	32 835 615	A	-0.0437	7.19E-06
101K	Controls	6	RS1580345	32 837 696	C	-0.04417	5.64E-06
101K	Controls	6	RS6906376	32 838 584	A	-0.0441	5.83E-06
101K	Controls	6	RS6929078	32 838 664	G	-0.04416	5.68E-06
101K	Controls	6	RS7453903	32 838 949	G	-0.04345	7.98E-06
101K	Controls	6	RS9276845	32 840 139	G	-0.04472	4.54E-06
101K	Controls	6	RS9276864	32 843 238	A	-0.0447	4.74E-06
101K	Controls	6	RS7757767	32 845 873	G	-0.04427	5.31E-06
101K	Controls	6	RS2621426	32 846 570	A	-0.0434	8.40E-06
101K	Controls	6	RS9276899	32 848 168	A	-0.04387	6.76E-06
101K	Controls	6	RS2170185	32 849 639	G	-0.04381	6.68E-06
101K	Controls	6	RS2127675	32 850 850	G	-0.04365	7.40E-06
101K	Controls	6	RS4947259	32 851 507	A	-0.04391	6.37E-06
101K	Controls	9	RS9406736	17 793 903	A	0.04771	7.00E-06
101K	Controls	13	RS11148543	61 707 375	A	0.04839	5.90E-06
101K	Controls	13	RS9317162	61 737 576	A	0.04826	6.77E-06
101K	Controls	13	RS9539055	61 755 041	A	0.05113	2.17E-06
101K	Controls	13	RS9539057	61 769 280	A	0.05091	2.28E-06
101K	Controls	15	RS10775256	92 997 155	G	0.04579	6.05E-06
101K	Controls	20	RS775121	16 046 227	A	-0.05066	8.21E-06
101K	All subjects	2	RS1569193	51 720 700	A	-0.03719	5.29E-06
101K	All subjects	4	RS10604	17 488 132	G	0.03208	2.47E-06
101K	All subjects	4	RS2597775	17 503 382	A	0.03127	4.52E-06
101K	All subjects	6	RS2735048	29 733 701	A	-0.03252	1.47E-06
101K	All subjects	6	RS2735046	29 734 098	A	-0.03087	2.89E-06
101K	All subjects	6	RS2523409	29 775 662	G	-0.03267	9.81E-07
101K	All subjects	6	RS3115627	29 820 278	G	-0.03098	5.13E-06
101K	All subjects	6	RS1611704	29 828 467	A	-0.03167	3.31E-06
101K	All subjects	6	RS2523822	29 828 660	G	-0.03151	5.16E-06
101K	All subjects	6	RS2860580	29 906 691	A	0.0283	8.79E-06
101K	All subjects	6	RS4713270	29 934 697	A	-0.0306	8.97E-06
101K	All subjects	6	RS4713275	29 937 580	C	-0.03082	8.81E-06
101K	All subjects	6	RS2256543	29 937 833	A	0.02804	9.87E-06
101K	All subjects	6	RS6916422	29 938 110	A	-0.03066	8.61E-06
101K	All subjects	6	RS3823355	29 942 083	A	-0.03073	8.45E-06
101K	All subjects	6	RS3823358	29 942 205	A	-0.03082	8.62E-06

101K	All subjects	6	RS6904029	29 943 067	A	-0.0308	8.08E-06
101K	All subjects	6	RS4713281	29 978 352	A	-0.03129	6.52E-06
101K	All subjects	6	RS9357092	29 984 252	A	-0.03168	5.00E-06
101K	All subjects	6	RS4711206	29 987 184	A	-0.03083	8.83E-06
101K	All subjects	6	RS3910312	30 008 746	C	-0.03159	5.27E-06
101K	All subjects	6	RS9380150	30 010 492	G	-0.03114	6.79E-06
101K	All subjects	6	RS9295829	30 028 800	G	-0.03129	6.34E-06
101K	All subjects	6	RS3807031	30 033 884	A	-0.03362	7.12E-06
101K	All subjects	6	RS9393989	30 040 084	A	-0.03135	6.22E-06
101K	All subjects	6	RS4711209	30 047 403	A	-0.03116	7.08E-06
101K	All subjects	6	RS4959041	30 077 967	G	-0.03281	1.90E-06
101K	All subjects	6	RS9261438	30 089 280	A	-0.03135	4.76E-06
101K	All subjects	6	RS9261639	30 237 859	A	-0.0318	7.16E-06
101K	All subjects	6	RS4713305	30 252 836	A	-0.03165	7.35E-06
101K	All subjects	6	RS2844571	31 335 647	G	-0.03392	7.19E-07
101K	All subjects	6	RS2253908	31 336 891	A	-0.03409	6.39E-07
101K	All subjects	6	RS9295986	31 338 528	A	0.03241	8.86E-06
101K	All subjects	6	RS9266629	31 346 822	G	-0.04199	8.10E-08
101K	All subjects	6	RS7741091	31 352 631	G	0.03793	1.03E-07
101K	All subjects	6	RS2523484	31 353 639	A	0.0336	8.90E-07
101K	All subjects	6	RS4711268	31 354 504	A	0.03823	1.14E-06
101K	All subjects	6	RS13437082	31 354 560	A	0.03898	6.92E-07
101K	All subjects	6	RS4711269	31 354 819	A	0.03892	7.15E-07
101K	All subjects	6	RS13437088	31 355 119	A	0.03885	7.51E-07
101K	All subjects	6	RS7751505	31 360 255	C	0.03748	1.99E-06
101K	All subjects	6	RS7751725	31 360 433	G	0.03826	1.24E-06
101K	All subjects	6	**RS2596530**	31387373	A	0.03513	**2.77E-08**
101K	All subjects	6	**RS2596531**	31387557	A	0.03552	**1.87E-08**
101K	All subjects	6	**RS2844511**	31389784	G	0.03542	**2.05E-08**
101K	All subjects	6	**RS2516448**	31390410	G	0.03558	**1.77E-08**
101K	All subjects	6	RS2523651	31 448 154	A	-0.03294	3.86E-07
101K	All subjects	6	RS2523650	31 449 022	A	-0.02988	8.51E-06
101K	All subjects	6	RS2269475	31 583 931	A	0.04192	2.02E-06
101K	All subjects	6	RS3763295	31 587 938	G	0.04406	6.74E-07
101K	All subjects	6	RS2242657	31 602 489	G	0.0406	3.82E-06
101K	All subjects	6	RS2295665	31 632 686	A	0.04194	1.78E-06
101K	All subjects	6	RS2295664	31 633 165	A	0.04167	2.06E-06
101K	All subjects	6	RS14365	31 635 710	G	0.04052	1.04E-06
101K	All subjects	6	RS2280800	31 646 398	A	0.04121	2.68E-06
101K	All subjects	6	RS2242653	31 675 765	A	0.04068	2.03E-06
101K	All subjects	6	RS2293861	31 711 124	A	0.04535	3.87E-07

101K	All subjects	6	RS2075788	31 712 181	C	0.04517	4.32E-07
101K	All subjects	6	RS3749953	31 713 124	G	0.04033	9.73E-06
101K	All subjects	6	RS3828922	31 713 454	A	0.04522	4.21E-07
101K	All subjects	6	RS6905572	31 731 881	A	0.0435	2.50E-06
101K	All subjects	6	RS35294112	31 794 443	G	-0.0863	1.57E-06
101K	All subjects	6	RS17201248	31 803 130	A	-0.0865	1.40E-06
101K	All subjects	6	RS397081	32 192 617	G	-0.07245	1.21E-06
101K	All subjects	6	RS2294878	32 367 795	A	0.03214	8.22E-07
101K	All subjects	6	RS6926737	32 375 745	A	0.03213	6.59E-07
101K	All subjects	6	RS3763317	32 376 788	A	0.03332	3.26E-07
101K	All subjects	6	RS9268503	32 377 061	A	0.03161	1.02E-06
101K	All subjects	6	RS5007265	32 378 866	C	0.03267	4.29E-07
101K	All subjects	6	RS5007263	32 378 982	G	0.0322	6.35E-07
101K	All subjects	6	RS5007259	32 379 101	G	0.03208	6.94E-07
101K	All subjects	6	RS6932810	32 380 190	A	0.03152	1.10E-06
101K	All subjects	6	RS6932542	32 380 262	G	0.03225	6.07E-07
101K	All subjects	6	RS6937545	32 418 031	A	-0.02979	4.45E-06
101K	All subjects	6	RS7763262	32 424 882	A	-0.03026	3.31E-06
101K	All subjects	6	RS660895	32 577 380	G	0.04164	1.10E-07
101K	All subjects	6	RS4947342	32 653 070	A	0.03708	1.46E-06
101K	All subjects	6	RS2856692	32 653 385	C	0.03743	1.19E-06
101K	All subjects	6	**RS7774434**	32657578	G	0.03808	**1.48E-08**
101K	All subjects	6	**RS9275224**	32659878	G	0.03766	**8.42E-09**
101K	All subjects	6	RS2858324	32 660 375	G	0.0343	1.47E-07
101K	All subjects	6	**RS9275245**	32660943	G	0.03877	**3.47E-09**
101K	All subjects	6	RS5000634	32 663 564	G	0.03433	7.96E-07
101K	All subjects	6	**RS6457617**	32663851	A	0.03921	**1.93E-09**
101K	All subjects	6	RS6457622	32 664 163	C	0.03445	7.11E-07
101K	All subjects	6	RS2647012	32 664 458	G	0.03403	1.79E-07
101K	All subjects	6	RS2647003	32 664 880	C	0.03434	1.40E-07
101K	All subjects	6	**RS9275312**	32665728	G	0.04905	**4.92E-09**
101K	All subjects	6	RS2856725	32 666 738	A	0.03393	1.95E-07
101K	All subjects	6	**RS9275328**	32666822	A	0.04896	**5.57E-09**
101K	All subjects	6	**RS9275330**	32666875	G	0.04862	**7.10E-09**
101K	All subjects	6	**RS9275333**	32666968	G	0.04852	**7.54E-09**
101K	All subjects	6	**RS9275371**	32668296	G	0.04466	**3.64E-10**
101K	All subjects	6	**RS9275374**	32668526	A	0.04495	**2.70E-10**
101K	All subjects	6	RS1612904	32 669 018	C	-0.03378	2.09E-07
101K	All subjects	6	**RS9275388**	32669084	G	0.04475	**3.26E-10**
101K	All subjects	6	**RS9275390**	32669156	G	0.04508	**2.52E-10**
101K	All subjects	6	**RS9275393**	32669439	A	0.04478	**3.53E-10**

101K	All subjects	6	**RS9275406**	32669955	A	0.04486	**3.02E-10**
101K	All subjects	6	**RS9275407**	32670037	A	0.0451	**2.38E-10**
101K	All subjects	6	**RS9275418**	32670244	G	0.04484	**2.97E-10**
101K	All subjects	6	RS2856717	32 670 308	G	0.03422	1.50E-07
101K	All subjects	6	RS2858305	32 670 464	A	0.03464	1.06E-07
101K	All subjects	6	**RS9275424**	32670576	G	0.0448	**3.15E-10**
101K	All subjects	6	**RS9275428**	32670978	G	0.04493	**3.09E-10**
101K	All subjects	6	**RS9275439**	32671521	G	0.04487	**3.21E-10**
101K	All subjects	6	RS9275523	32 674 994	A	0.03628	1.62E-06
101K	All subjects	6	RS9275582	32 680 070	A	0.03789	6.16E-07
101K	All subjects	6	RS9275595	32 681 355	G	0.03688	1.22E-06
101K	All subjects	6	RS9275596	32 681 631	G	-0.03509	7.08E-08
101K	All subjects	6	RS6931062	32 835 615	A	-0.03068	5.12E-06
101K	All subjects	6	RS1580345	32 837 696	C	-0.03091	4.26E-06
101K	All subjects	6	RS6906376	32 838 584	A	-0.03078	4.68E-06
101K	All subjects	6	RS6929078	32 838 664	G	-0.03106	3.82E-06
101K	All subjects	6	RS7453903	32 838 949	G	-0.03059	5.37E-06
101K	All subjects	6	RS9276845	32 840 139	G	-0.03121	3.65E-06
101K	All subjects	6	RS9276864	32 843 238	A	-0.03066	5.57E-06
101K	All subjects	6	RS7757767	32 845 873	G	-0.03112	3.63E-06
101K	All subjects	6	RS2621426	32 846 570	A	-0.03045	6.13E-06
101K	All subjects	6	RS9276899	32 848 168	A	-0.03079	4.81E-06
101K	All subjects	6	RS9276900	32 848 566	C	-0.02999	9.61E-06
101K	All subjects	6	RS2170185	32 849 639	G	-0.03081	4.60E-06
101K	All subjects	6	RS2127675	32 850 850	G	-0.03075	4.89E-06
101K	All subjects	6	RS4947259	32 851 507	A	-0.03088	4.34E-06
101K	All subjects	19	RS7254645	54 775 389	G	0.03379	2.57E-07
101K	All subjects	19	RS8100863	54 789 266	C	0.02862	8.04E-06
101K	All subjects	19	RS6509860	54 792 358	A	0.03142	2.23E-06

Table S8. Antibody responses analyzed for association with non-HLA SNPs serostatus

Antigen	Subject group	Chromo-some	SNP marker	Basepair position	Minor allele	OR (L95-U95)*	p
IE1A	MS cases	1	RS12566754	166 444 011	A	0.7968 (0.7209-0.8808)	8,83E-06
IE1A	MS cases	3	RS17129314	197 031 608	G	0.5514 (0.4282-0.7101)	3,99E-06
IE1A	MS cases	4	RS17277682	161 479 466	A	0.7201 (0.6291-0.8243)	1,91E-06
IE1A	MS cases	4	RS11936687	163 288 053	A	1.31 (1.175-1.46)	1,05E-06
IE1A	MS cases	6	RS3130544	31 058 340	A	0.7114 (0.6139-0.8244)	5,97E-06
IE1A	MS cases	6	RS3130557	31 094 703	A	0.705 (0.6082-0.8172)	3,48E-06

IE1A	MS cases	6	RS7750641	31 129 310	A	0.7017 (0.6056-0.8131)	2,46E-06
IE1A	MS cases	6	RS3132510	31 172 151	G	0.6693 (0.5736-0.7811)	3,49E-07
IE1A	MS cases	6	RS2596487	31 325 056	A	0.6903 (0.6037-0.7894)	6,13E-08
IE1A	MS cases	6	**RS2523573**	31 328 988	C	0.6381 (0.5495-0.7409)	**3,82E-09**
IE1A	MS cases	6	RS9266669	31 348 077	A	0.6781 (0.5882-0.7817)	8,61E-08
IE1A	MS cases	6	**RS3094013**	31 434 366	A	0.6379 (0.5493-0.7408)	**3,76E-09**
IE1A	MS cases	6	**RS3131618**	31 434 621	G	0.6413 (0.5517-0.7454)	**7,14E-09**
IE1A	MS cases	6	**RS3131643**	31 442 782	A	0.6774 (0.5898-0.7781)	**3,63E-08**
IE1A	MS cases	6	**RS3099844**	31 448 976	A	0.6261 (0.5393-0.7269)	**7,81E-10**
IE1A	MS cases	6	**RS3094011**	31 451 836	G	0.6299 (0.5422-0.7317)	**1,49E-09**
IE1A	MS cases	6	**RS9267445**	31 483 481	C	0.6372 (0.5476-0.7414)	**5,50E-09**
IE1A	MS cases	6	RS3093988	31 492 453	A	0.6958 (0.608-0.7964)	1,38E-07
IE1A	MS cases	6	RS2516482	31 496 569	G	0.7055 (0.6166-0.8071)	3,75E-07
IE1A	MS cases	6	RS2734583	31 505 480	G	0.6671 (0.5745-0.7747)	1,12E-07
IE1A	MS cases	6	RS1800629	31 543 031	A	0.7129 (0.6236-0.815)	7,23E-07
IE1A	MS cases	6	RS3117582	31 620 520	C	0.6607 (0.5668-0.7702)	1,17E-07
IE1A	MS cases	6	RS9267531	31 636 742	G	0.6607 (0.5668-0.7702)	1,17E-07
IE1A	MS cases	6	RS3101018	31 705 864	A	0.6578 (0.5635-0.7679)	1,14E-07
IE1A	MS cases	6	RS3132445	31 712 196	A	0.6635 (0.5691-0.7737)	1,64E-07
IE1A	MS cases	6	RS3130484	31 715 882	G	0.6619 (0.5677-0.7716)	1,36E-07
IE1A	MS cases	6	RS3131379	31 721 033	A	0.6643 (0.5697-0.7746)	1,81E-07
IE1A	MS cases	6	RS3117574	31 725 230	A	0.6649 (0.5703-0.7752)	1,88E-07
IE1A	MS cases	6	RS3131378	31 725 285	G	0.6612 (0.5671-0.7709)	1,27E-07
IE1A	MS cases	6	RS3117577	31 727 474	G	0.6611 (0.5671-0.7708)	1,26E-07
IE1A	MS cases	6	RS3101017	31 733 466	G	0.6653 (0.5707-0.7755)	1,88E-07
IE1A	MS cases	6	RS652888	31 851 234	G	0.755 (0.6673-0.8541)	7,94E-06
IE1A	MS cases	6	RS558702	31 870 326	A	0.6539 (0.5606-0.7628)	6,46E-08
IE1A	MS cases	6	**RS519417**	31 878 433	A	0.6458 (0.5533-0.7538)	**2,98E-08**
IE1A	MS cases	6	**RS497309**	31 892 484	C	0.6496 (0.5571-0.7575)	**3,72E-08**
IE1A	MS cases	6	**RS1270942**	31 918 860	G	0.65 (0.5574-0.7578)	**3,82E-08**
IE1A	MS cases	6	RS1150755	32 038 550	A	0.7106 (0.6203-0.8141)	8,42E-07
IE1A	MS cases	6	RS7774197	32 046 275	C	1.728 (1.358-2.198)	8,42E-06
IE1A	MS cases	6	RS1150754	32 050 758	A	0.7087 (0.6186-0.8119)	6,92E-07
IE1A	MS cases	6	RS1150753	32 059 867	G	0.6557 (0.5621-0.7648)	7,72E-08
IE1A	MS cases	6	RS1150752	32 064 726	G	0.6557 (0.5617-0.7655)	9,06E-08
IE1A	MS cases	6	RS1269852	32 080 191	C	0.659 (0.5648-0.769)	1,17E-07
IE1A	MS cases	6	RS3130288	32 096 001	A	0.6606 (0.5663-0.7706)	1,33E-07
IE1A	MS cases	6	RS3134608	32 117 971	C	0.7446 (0.6537-0.8481)	8,94E-06
IE1A	MS cases	6	RS3096697	32 134 510	A	0.7413 (0.6507-0.8445)	6,74E-06
IE1A	MS cases	6	RS3130347	32 134 656	G	0.7438 (0.653-0.8472)	8,37E-06
IE1A	MS cases	6	RS3130284	32 140 487	G	0.7441 (0.6532-0.8477)	8,77E-06

IE1A	MS cases	6	RS3134947	32 145 205	A	0.744 (0.6531-0.8475)	8,59E-06
IE1A	MS cases	6	RS3134945	32 146 492	A	0.7439 (0.6529-0.8475)	8,72E-06
IE1A	MS cases	6	RS3130349	32 147 696	A	0.7167 (0.6263-0.82)	1,26E-06
IE1A	MS cases	6	RS1800625	32 152 442	G	0.7172 (0.6269-0.8206)	1,32E-06
IE1A	MS cases	6	RS204993	32 155 581	G	0.7449 (0.6591-0.8419)	2,42E-06
IE1A	MS cases	6	RS204991	32 161 366	G	0.7257 (0.6342-0.8304)	3,14E-06
IE1A	MS cases	6	RS204990	32 161 430	A	0.7195 (0.6279-0.8244)	2,13E-06
IE1A	MS cases	6	RS2071278	32 165 444	G	0.686 (0.5945-0.7916)	2,45E-07
IE1A	MS cases	6	**RS3134942**	32 168 771	A	0.657 (0.5666-0.7619)	**2,68E-08**
IE1A	MS cases	6	RS2071277	32 171 683	G	0.7884 (0.7112-0.8741)	6,24E-06
IE1A	MS cases	6	**RS3131296**	32 172 993	A	0.6535 (0.563-0.7585)	**2,20E-08**
IE1A	MS cases	6	**RS3132956**	32 179 438	A	0.6583 (0.5677-0.7632)	**3,05E-08**
IE1A	MS cases	6	RS3131290	32 183 175	A	0.7554 (0.6789-0.8405)	2,60E-07
IE1A	MS cases	6	RS3134799	32 184 221	A	0.7765 (0.6995-0.862)	2,05E-06
IE1A	MS cases	6	RS436388	32 186 264	G	0.7947 (0.7176-0.88)	9,98E-06
IE1A	MS cases	6	RS394657	32 187 023	G	0.7797 (0.7025-0.8654)	2,93E-06
IE1A	MS cases	6	RS1109771	32 187 605	G	0.7939 (0.7169-0.8792)	9,36E-06
IE1A	MS cases	6	**RS915894**	32 190 390	C	0.7146 (0.6406-0.797)	**1,62E-09**
IE1A	MS cases	6	RS3134930	32 191 620	A	0.7267 (0.641-0.8239)	6,21E-07
IE1A	MS cases	6	**RS6901158**	32 205 942	A	0.6498 (0.563-0.7499)	**3,75E-09**
IE1A	MS cases	6	RS424232	32 208 324	A	0.7284 (0.6476-0.8192)	1,26E-07
IE1A	MS cases	6	**RS3132971**	32 230 256	C	0.6315 (0.5409-0.7372)	**5,94E-09**
IE1A	MS cases	6	**RS7775397**	32 261 252	C	0.6366 (0.5454-0.743)	**1,02E-08**
IE1A	MS cases	6	RS9268219	32 284 108	C	0.6475 (0.5525-0.7588)	7,82E-08
IE1A	MS cases	6	**RS9268235**	32 290 208	A	0.6396 (0.5478-0.7468)	**1,57E-08**
IE1A	MS cases	6	RS1265760	32 321 872	G	0.797 (0.7207-0.8814)	9,99E-06
IE1A	MS cases	6	RS3129911	32 328 403	A	0.797 (0.7207-0.8814)	9,99E-06
IE1A	MS cases	6	RS3129922	32 333 098	A	0.7933 (0.7172-0.8775)	6,77E-06
IE1A	MS cases	6	RS2273017	32 337 630	A	0.7792 (0.7039-0.8624)	1,46E-06
IE1A	MS cases	6	**RS2073045**	32 339 548	A	0.7202 (0.6463-0.8027)	**2,92E-09**
IE1A	MS cases	6	**RS2894254**	32 345 689	C	0.6423 (0.5498-0.7504)	**2,42E-08**
IE1A	MS cases	6	RS3129843	32 395 726	G	0.6511 (0.5574-0.7606)	6,28E-08
IE1A	MS cases	6	RS3763326	32 413 557	G	2.179 (1.577-3.012)	2,36E-06
IE1A	MS cases	6	RS9270665	32 566 232	A	0.7426 (0.6527-0.8449)	6,17E-06
IE1A	MS cases	6	RS642093	32 582 075	A	0.7431 (0.6544-0.8438)	4,69E-06
IE1A	MS cases	6	RS3129763	32 590 925	A	0.7427 (0.6529-0.8449)	6,14E-06
IE1A	MS cases	6	RS9272219	32 602 269	A	0.7528 (0.6705-0.8451)	1,50E-06
IE1A	MS cases	6	RS2187668	32 605 884	A	0.7031 (0.607-0.8143)	2,60E-06
IE1A	MS cases	6	RS9273012	32 611 641	G	0.7504 (0.6685-0.8423)	1,11E-06
IE1A	MS cases	6	**RS17843604**	32 620 283	A	0.7155 (0.6429-0.7963)	**8,66E-10**
IE1A	MS cases	6	RS9273327	32 623 223	C	0.7037 (0.6066-0.8163)	3,52E-06

IE1A	MS cases	6	**RS9273349**	32 625 869	G	0.6862 (0.6161-0.7643)	**7,37E-12**
IE1A	MS cases	6	**RS9273363**	32 626 272	A	0.7099 (0.6336-0.7955)	**3,60E-09**
IE1A	MS cases	6	**RS6906021**	32 626 311	G	0.7379 (0.6617-0.8229)	**4,59E-08**
IE1A	MS cases	6	**RS1063355**	32 627 714	C	0.6865 (0.6164-0.7647)	**7,91E-12**
IE1A	MS cases	6	RS9273448	32 627 747	A	1.335 (1.202-1.483)	6,32E-08
IE1A	MS cases	6	RS2854275	32 628 428	A	0.6999 (0.6043-0.8105)	1,89E-06
IE1A	MS cases	6	RS1794282	32 666 526	A	0.6545 (0.5601-0.7648)	9,61E-08
IE1A	MS cases	6	RS3135006	32 667 119	A	1.335 (1.202-1.484)	7,37E-08
IE1A	MS cases	6	RS4947344	32 677 846	G	0.7733 (0.6983-0.8564)	8,04E-07
IE1A	MS cases	6	RS9276689	32 751 962	A	0.6455 (0.5489-0.7591)	1,22E-07
IE1A	MS cases	6	RS7762279	32 755 290	G	0.6421 (0.5464-0.7545)	7,31E-08
IE1A	MS cases	6	RS4947350	32 767 620	G	0.742 (0.6573-0.8377)	1,42E-06
IE1A	MS cases	6	RS11244	32 780 724	A	0.7655 (0.6825-0.8586)	5,02E-06
IE1A	MS cases	6	**RS3819720**	32 804 570	A	0.7304 (0.6548-0.8147)	**1,77E-08**
IE1A	MS cases	6	**RS241425**	32 804 909	G	0.7515 (0.6797-0.8309)	**2,48E-08**
IE1A	MS cases	6	RS241424	32 804 934	A	0.7698 (0.6958-0.8516)	3,84E-07
IE1A	MS cases	6	RS2239701	32 805 049	G	0.7565 (0.683-0.8379)	8,84E-08
IE1A	MS cases	6	RS2071465	32 805 470	C	0.7743 (0.6987-0.8581)	1,08E-06
IE1A	MS cases	6	RS3763366	32 807 446	C	0.789 (0.7131-0.8729)	4,32E-06
IE1A	MS cases	6	RS3763349	32 808 232	G	0.7837 (0.7085-0.8669)	2,20E-06
IE1A	MS cases	6	RS9276810	32 810 443	A	0.7892 (0.7132-0.8734)	4,70E-06
IE1A	MS cases	6	RS406477	33 005 644	G	0.718 (0.6229-0.8275)	4,81E-06
IE1A	MS cases	6	RS987870	33 042 880	G	0.6726 (0.5686-0.7955)	3,66E-06
IE1A	MS cases	6	RS2071354	33 044 388	G	0.6733 (0.5692-0.7963)	3,86E-06
IE1A	MS cases	6	RS1042151	33 048 661	G	0.732 (0.6474-0.8276)	6,39E-07
IE1A	MS cases	6	RS9277378	33 050 279	G	0.7644 (0.6856-0.8523)	1,31E-06
IE1A	MS cases	6	RS9277386	33 050 499	G	0.7561 (0.678-0.8432)	5,00E-07
IE1A	MS cases	6	RS9277396	33 051 139	A	0.7774 (0.698-0.8658)	4,65E-06
IE1A	MS cases	6	RS9277426	33 051 910	A	0.7768 (0.6976-0.8651)	4,23E-06
IE1A	MS cases	6	RS9277469	33 053 468	A	0.7754 (0.6963-0.8636)	3,66E-06
IE1A	MS cases	6	RS9277471	33 053 682	A	0.7777 (0.6983-0.8661)	4,70E-06
IE1A	MS cases	6	RS9277533	33 054 721	A	0.7788 (0.6993-0.8674)	5,36E-06
IE1A	MS cases	6	RS9277546	33 055 346	C	0.7764 (0.6971-0.8647)	4,11E-06
IE1A	MS cases	6	RS9277554	33 055 538	A	0.7768 (0.6976-0.8651)	4,23E-06
IE1A	MS cases	6	RS3117228	33 056 435	A	0.7764 (0.6972-0.8647)	4,05E-06
IE1A	MS cases	6	RS3130188	33 057 176	G	0.7756 (0.6964-0.8637)	3,68E-06
IE1A	MS cases	6	RS3091282	33 057 198	C	0.7757 (0.6965-0.8639)	3,80E-06
IE1A	MS cases	6	RS3117226	33 057 659	A	0.7683 (0.6884-0.8576)	2,58E-06
IE1A	MS cases	6	RS3117225	33 057 711	A	0.7752 (0.6959-0.8635)	3,71E-06
IE1A	MS cases	6	RS3097652	33 057 835	A	0.7772 (0.6977-0.8658)	4,70E-06
IE1A	MS cases	6	RS2179920	33 058 874	A	0.7517 (0.6699-0.8435)	1,20E-06

IE1A	MS cases	6	RS6914616	33 063 592	A	0.5906 (0.4706-0.7412)	5,50E-06
IE1A	MS cases	6	RS2179915	33 065 734	A	0.591 (0.4709-0.7417)	5,66E-06
IE1A	MS cases	6	RS3117239	33 071 777	G	0.7571 (0.6748-0.8495)	2,17E-06
IE1A	MS cases	6	RS2064478	33 072 266	A	0.7572 (0.6748-0.8496)	2,19E-06
IE1A	MS cases	6	RS2064476	33 073 322	G	0.7769 (0.6977-0.8651)	4,20E-06
IE1A	MS cases	6	RS3117234	33 073 984	G	0.7479 (0.6674-0.838)	5,67E-07
IE1A	MS cases	6	RS3117231	33 074 908	G	0.7734 (0.6929-0.8632)	4,57E-06
IE1A	MS cases	6	RS3117230	33 075 635	G	0.7572 (0.6748-0.8496)	2,19E-06
IE1A	MS cases	6	RS3128930	33 075 666	A	0.746 (0.6663-0.8353)	3,75E-07
IE1A	MS cases	10	RS12255701	90 532 552	A	1.568 (1.292-1.902)	5,22E-06
IE1A	MS cases	12	RS706536	26 130 945	G	0.7792 (0.7026-0.8642)	2,34E-06
IE1A	MS cases	22	RS1061660	30 819 628	G	0.7739 (0.6931-0.8641)	5,16E-06
IE1A	Controls	3	RS7651713	111399209	A	1.382 (1.21-1.578)	1,89E-06
IE1A	Controls	3	RS1355767	111416310	A	1.342 (1.185-1.521)	3,86E-06
IE1A	Controls	6	RS1737031	29737481	A	0.7539 (0.6678-0.851)	4,95E-06
IE1A	Controls	6	RS1633030	29745794	G	0.7534 (0.6673-0.8505)	4,68E-06
IE1A	Controls	6	RS1633028	29746367	C	0.7555 (0.6692-0.853)	5,94E-06
IE1A	Controls	6	RS2735042	29747373	G	0.7547 (0.6686-0.852)	5,36E-06
IE1A	Controls	6	RS1610649	29768917	G	0.7582 (0.6743-0.8524)	3,65E-06
IE1A	Controls	6	RS1632983	29773509	A	0.7595 (0.6735-0.8564)	7,17E-06
IE1A	Controls	6	RS1610678	29789190	G	0.7423 (0.6642-0.8295)	1,48E-07
IE1A	Controls	6	RS1611149	29789999	A	0.7429 (0.6648-0.8301)	1,53E-07
IE1A	Controls	6	**RS1736936**	29794317	A	0.7155 (0.6395-0.8005)	**5,09E-09**
IE1A	Controls	6	RS915669	29798425	A	0.7384 (0.6607-0.8253)	9,04E-08
IE1A	Controls	6	RS1611133	29809382	A	0.7241 (0.6329-0.8286)	2,67E-06
IE1A	Controls	6	RS1611750	29814778	C	0.7164 (0.6258-0.8201)	1,34E-06
IE1A	Controls	6	RS2247504	29819077	A	0.7688 (0.6867-0.8608)	5,09E-06
IE1A	Controls	6	RS2517854	29823296	G	0.7751 (0.6923-0.8678)	9,85E-06
IE1A	Controls	6	RS1611737	29831571	G	0.7713 (0.689-0.8634)	6,41E-06
IE1A	Controls	6	RS2844806	29933439	A	0.7791 (0.6978-0.8699)	9,01E-06
IE1A	Controls	6	RS1362104	30101656	A	0.7449 (0.6655-0.8337)	3,02E-07
IE1A	Controls	6	RS3130544	31058340	A	0.6651 (0.5605-0.7891)	2,94E-06
IE1A	Controls	6	RS3130557	31094703	A	0.6558 (0.5524-0.7785)	1,44E-06
IE1A	Controls	6	RS7750641	31129310	A	0.6638 (0.5594-0.7877)	2,68E-06
IE1A	Controls	6	RS3132510	31172151	G	0.6528 (0.5461-0.7805)	2,87E-06
IE1A	Controls	6	RS2523573	31328988	C	0.6521 (0.5482-0.7757)	1,38E-06
IE1A	Controls	6	RS9266669	31348077	A	0.6609 (0.5635-0.7751)	3,53E-07
IE1A	Controls	6	RS3094013	31434366	A	0.6559 (0.5517-0.7798)	1,77E-06
IE1A	Controls	6	RS3131618	31434621	G	0.647 (0.5436-0.7702)	9,69E-07
IE1A	Controls	6	RS3131643	31442782	A	0.707 (0.6076-0.8227)	7,34E-06

IE1A	Controls	6	RS3099844	31448976	A	0.6423 (0.5407-0.7631)	4,75E-07
IE1A	Controls	6	RS3094011	31451836	G	0.6333 (0.5327-0.7528)	2,24E-07
IE1A	Controls	6	RS9267445	31483481	C	0.6473 (0.5432-0.7714)	1,16E-06
IE1A	Controls	6	RS2734583	31505480	G	0.6514 (0.548-0.7744)	1,18E-06
IE1A	Controls	6	RS3117582	31620520	C	0.658 (0.5522-0.784)	2,84E-06
IE1A	Controls	6	**RS805262**	31628733	A	0.7375 (0.662-0.8215)	**3,22E-08**
IE1A	Controls	6	RS9267531	31636742	G	0.6593 (0.5532-0.7859)	3,34E-06
IE1A	Controls	6	RS3101018	31705864	A	0.6566 (0.5504-0.7832)	2,94E-06
IE1A	Controls	6	RS3132445	31712196	A	0.6594 (0.5534-0.7857)	3,21E-06
IE1A	Controls	6	RS3130484	31715882	G	0.6585 (0.5526-0.7846)	2,98E-06
IE1A	Controls	6	RS3131379	31721033	A	0.6618 (0.5552-0.7889)	4,13E-06
IE1A	Controls	6	RS3117574	31725230	A	0.6585 (0.5526-0.7847)	2,98E-06
IE1A	Controls	6	RS3131378	31725285	G	0.6573 (0.5517-0.7833)	2,71E-06
IE1A	Controls	6	RS3117577	31727474	G	0.6581 (0.5523-0.7842)	2,91E-06
IE1A	Controls	6	RS3101017	31733466	G	0.6568 (0.5512-0.7827)	2,62E-06
IE1A	Controls	6	RS558702	31870326	A	0.6619 (0.5555-0.7886)	3,86E-06
IE1A	Controls	6	RS519417	31878433	A	0.6633 (0.5568-0.7902)	4,30E-06
IE1A	Controls	6	RS511294	31888869	C	1.728 (1.358-2.2)	8,68E-06
IE1A	Controls	6	RS497309	31892484	C	0.6607 (0.5546-0.7871)	3,45E-06
IE1A	Controls	6	RS1270942	31918860	G	0.6584 (0.5525-0.7845)	2,96E-06
IE1A	Controls	6	RS1150755	32038550	A	0.6912 (0.5938-0.8045)	1,86E-06
IE1A	Controls	6	RS1150754	32050758	A	0.6911 (0.5937-0.8044)	1,85E-06
IE1A	Controls	6	RS1150753	32059867	G	0.6595 (0.5538-0.7854)	3,02E-06
IE1A	Controls	6	RS1150752	32064726	G	0.6407 (0.5371-0.7644)	7,70E-07
IE1A	Controls	6	RS411337	32077380	A	1.597 (1.309-1.947)	3,74E-06
IE1A	Controls	6	RS1269852	32080191	C	0.6737 (0.5662-0.8016)	8,44E-06
IE1A	Controls	6	RS9469084	32080383	A	1.576 (1.293-1.922)	6,83E-06
IE1A	Controls	6	RS204888	32089142	A	1.576 (1.293-1.921)	6,86E-06
IE1A	Controls	6	RS3130288	32096001	A	0.6672 (0.5606-0.7941)	5,19E-06
IE1A	Controls	6	RS169494	32097876	A	1.589 (1.303-1.938)	4,81E-06
IE1A	Controls	6	RS3130349	32147696	A	0.7151 (0.6173-0.8283)	7,75E-06
IE1A	Controls	6	RS1800625	32152442	G	0.7157 (0.618-0.8289)	8,03E-06
IE1A	Controls	6	RS2071287	32170433	A	0.7567 (0.6795-0.8425)	3,70E-07
IE1A	Controls	6	RS2071277	32171683	G	0.7634 (0.6861-0.8493)	6,97E-07
IE1A	Controls	6	RS206017	32176211	G	1.335 (1.198-1.488)	1,89E-07
IE1A	Controls	6	RS206018	32177880	C	1.359 (1.186-1.557)	9,79E-06
IE1A	Controls	6	**RS3131290**	32183175	A	0.7269 (0.6503-0.8126)	**2,03E-08**
IE1A	Controls	6	**RS3134799**	32184221	A	0.7257 (0.6509-0.8091)	**7,57E-09**
IE1A	Controls	6	RS436388	32186264	A	1.343 (1.205-1.497)	1,03E-07
IE1A	Controls	6	RS379464	32186348	A	1.79 (1.39-2.306)	6,57E-06
IE1A	Controls	6	**RS394657**	32187023	G	0.7305 (0.6554-0.8143)	**1,45E-08**

IE1A	Controls	6	RS1109771	32187605	A	1.303 (1.169-1.452)	1,71E-06
IE1A	Controls	6	RS8192584	32189192	A	2.057 (1.532-2.76)	1,56E-06
IE1A	Controls	6	RS915894	32190390	C	0.7427 (0.6617-0.8335)	4,31E-07
IE1A	Controls	6	RS3132971	32230256	C	0.638 (0.5351-0.7606)	5,44E-07
IE1A	Controls	6	RS7775397	32261252	C	0.6413 (0.5382-0.7642)	6,81E-07
IE1A	Controls	6	RS9268235	32290208	A	0.6407 (0.5377-0.7635)	6,46E-07
IE1A	Controls	6	RS2894254	32345689	C	0.6375 (0.5343-0.7608)	5,97E-07
IE1A	Controls	6	RS3129843	32395726	G	0.6385 (0.5351-0.7618)	6,45E-07
IE1A	Controls	6	RS9270665	32566232	A	0.7231 (0.6293-0.8309)	4,82E-06
IE1A	Controls	6	RS3129763	32590925	A	0.7249 (0.6311-0.8327)	5,39E-06
IE1A	Controls	6	RS9272219	32602269	A	0.7368 (0.6493-0.8362)	2,21E-06
IE1A	Controls	6	RS9273012	32611641	G	0.7348 (0.6476-0.8338)	1,76E-06
IE1A	Controls	6	RS17843604	32620283	G	1.354 (1.211-1.513)	9,34E-08
IE1A	Controls	6	**RS9273349**	32625869	A	1.402 (1.254-1.567)	**2,52E-09**
IE1A	Controls	6	RS9273363	32626272	A	0.7303 (0.6489-0.8219)	1,84E-07
IE1A	Controls	6	**RS1063355**	32627714	A	1.404 (1.256-1.569)	**2,25E-09**
IE1A	Controls	6	RS9273448	32627747	A	1.325 (1.176-1.493)	3,91E-06
IE1A	Controls	6	RS1794282	32666526	A	0.6358 (0.5326-0.7589)	5,30E-07
IE1A	Controls	6	RS3135006	32667119	A	1.333 (1.182-1.503)	2,67E-06
IE1A	Controls	6	RS9276689	32751962	A	0.6379 (0.5282-0.7703)	3,00E-06
IE1A	Controls	6	RS7762279	32755290	G	0.6387 (0.5289-0.7711)	3,13E-06
IE1A	Controls	6	RS3819720	32804570	A	0.7592 (0.6766-0.8518)	2,72E-06
IE1A	Controls	6	RS1480380	32913246	A	0.626 (0.5105-0.7675)	6,67E-06

IE1A	All subjects	1	RS595980	20 827 216	A	1.234 (1.126-1.353)	6,76E-06
IE1A	All subjects	1	RS476622	20 853 195	A	1.246 (1.137-1.366)	2,58E-06
IE1A	All subjects	3	RS7641571	162 749 836	G	1.389 (1.203-1.604)	7,61E-06
IE1A	All subjects	6	RS7767008	28 630 793	C	0.8246 (0.7584-0.8966)	6,27E-06
IE1A	All subjects	6	RS1233579	28 712 663	G	0.7476 (0.6608-0.8459)	3,88E-06
IE1A	All subjects	6	RS1233599	28 731 188	C	0.7506 (0.6634-0.8492)	5,26E-06
IE1A	All subjects	6	RS1233619	28 745 452	A	0.754 (0.6662-0.8535)	7,91E-06
IE1A	All subjects	6	RS1311918	28 753 646	A	0.7552 (0.6671-0.8549)	9,14E-06
IE1A	All subjects	6	RS7767099	28 768 698	A	0.7534 (0.6656-0.8527)	7,41E-06
IE1A	All subjects	6	RS2765220	28 769 671	G	0.778 (0.702-0.8622)	1,70E-06
IE1A	All subjects	6	RS3131343	28 775 564	A	0.7508 (0.6634-0.8498)	5,71E-06
IE1A	All subjects	6	RS4324798	28 776 117	A	0.7536 (0.6658-0.8531)	7,71E-06
IE1A	All subjects	6	RS3132389	28 831 021	C	0.7491 (0.6619-0.8478)	4,78E-06
IE1A	All subjects	6	RS3118370	28 833 101	C	0.7546 (0.6661-0.8548)	9,58E-06
IE1A	All subjects	6	RS3131093	28 837 437	A	0.7514 (0.664-0.8504)	5,97E-06
IE1A	All subjects	6	RS3132392	28 838 629	G	0.7513 (0.6639-0.8503)	5,91E-06
IE1A	All subjects	6	RS3135309	28 855 805	C	0.7491 (0.6624-0.8473)	4,26E-06

IE1A	All subjects	6	RS3135316	28 864 849	G	0.7489 (0.6622-0.847)	4,16E-06
IE1A	All subjects	6	RS2230683	28 891 176	G	0.75 (0.6631-0.8483)	4,69E-06
IE1A	All subjects	6	RS3118361	28 898 287	A	0.74 (0.6535-0.8379)	2,04E-06
IE1A	All subjects	6	RS3130895	28 905 791	A	0.7465 (0.66-0.8444)	3,34E-06
IE1A	All subjects	6	RS3131073	28 920 972	A	0.7428 (0.6565-0.8405)	2,41E-06
IE1A	All subjects	6	RS3130845	28 923 367	G	0.7479 (0.6613-0.8459)	3,79E-06
IE1A	All subjects	6	RS3130837	28 948 092	A	0.7403 (0.6545-0.8375)	1,75E-06
IE1A	All subjects	6	RS3129791	28 954 293	A	0.7471 (0.6605-0.8451)	3,56E-06
IE1A	All subjects	6	RS3130891	28 977 217	C	0.7455 (0.659-0.8435)	3,11E-06
IE1A	All subjects	6	RS3130893	28 980 707	G	0.7466 (0.66-0.8446)	3,38E-06
IE1A	All subjects	6	RS3117143	29 031 142	A	0.7403 (0.6544-0.8375)	1,78E-06
IE1A	All subjects	6	RS3129173	29 159 629	C	0.7381 (0.6527-0.8347)	1,31E-06
IE1A	All subjects	6	RS3116830	29 167 575	A	0.7394 (0.6536-0.8365)	1,61E-06
IE1A	All subjects	6	RS3117326	29 240 378	A	0.7392 (0.6534-0.8362)	1,58E-06
IE1A	All subjects	6	RS3130834	29 248 149	G	0.7403 (0.6543-0.8375)	1,77E-06
IE1A	All subjects	6	RS3117439	29 266 483	A	0.7573 (0.6709-0.8548)	6,82E-06
IE1A	All subjects	6	RS3117433	29 303 364	G	0.7861 (0.7092-0.8714)	4,60E-06
IE1A	All subjects	6	RS3749971	29 342 775	A	0.7496 (0.6639-0.8463)	3,26E-06
IE1A	All subjects	6	RS9257809	29 356 331	G	0.7575 (0.6718-0.8541)	5,78E-06
IE1A	All subjects	6	RS442694	29 356 687	A	0.7487 (0.6636-0.8448)	2,63E-06
IE1A	All subjects	6	RS429479	29 372 323	G	0.7598 (0.6742-0.8562)	6,61E-06
IE1A	All subjects	6	RS1535039	29 411 432	G	0.7442 (0.6596-0.8395)	1,56E-06
IE1A	All subjects	6	RS2746149	29 435 355	G	0.746 (0.6615-0.8413)	1,79E-06
IE1A	All subjects	6	RS2746150	29 442 701	A	0.7441 (0.6595-0.8394)	1,54E-06
IE1A	All subjects	6	RS404240	29 523 957	G	0.7435 (0.6592-0.8385)	1,37E-06
IE1A	All subjects	6	RS1235162	29 537 224	G	0.7433 (0.6608-0.8362)	7,91E-07
IE1A	All subjects	6	RS444189	29 605 935	C	0.832 (0.7711-0.8977)	2,09E-06
IE1A	All subjects	6	RS396660	29 646 165	A	0.8102 (0.7486-0.8768)	1,79E-07
IE1A	All subjects	6	RS445150	29 646 879	G	0.809 (0.7476-0.8756)	1,49E-07
IE1A	All subjects	6	RS2747430	29 648 506	A	0.81 (0.7483-0.8768)	1,85E-07
IE1A	All subjects	6	RS3117301	29 654 700	A	0.8207 (0.753-0.8946)	6,94E-06
IE1A	All subjects	6	RS2747457	29 656 417	C	0.8201 (0.7525-0.8938)	6,32E-06
IE1A	All subjects	6	RS2747460	29 657 127	A	0.8209 (0.7532-0.8948)	7,10E-06
IE1A	All subjects	6	RS3129039	29 658 864	A	0.8185 (0.7494-0.8939)	8,36E-06
IE1A	All subjects	6	RS3094736	29 662 374	T	0.8409 (0.7813-0.9051)	3,94E-06
IE1A	All subjects	6	RS3129066	29 668 537	G	0.8285 (0.7625-0.9001)	8,83E-06
IE1A	All subjects	6	RS2107203	29 669 830	A	0.8277 (0.7617-0.8993)	8,05E-06
IE1A	All subjects	6	RS3129055	29 670 261	G	0.8272 (0.7613-0.8988)	7,55E-06
IE1A	All subjects	6	RS9258122	29 671 740	A	0.827 (0.7611-0.8987)	7,40E-06
IE1A	All subjects	6	RS1633041	29 733 223	A	0.8265 (0.7625-0.8958)	3,57E-06
IE1A	All subjects	6	RS1737041	29 736 229	A	0.8264 (0.7624-0.8957)	3,50E-06

IE1A	All subjects	6	RS1737031	29 737 481	A	0.8228 (0.7599-0.8908)	1,50E-06
IE1A	All subjects	6	RS1362068	29 742 108	G	0.8364 (0.7739-0.9038)	6,36E-06
IE1A	All subjects	6	RS1362070	29 742 299	G	0.836 (0.7736-0.9034)	6,01E-06
IE1A	All subjects	6	RS1615962	29 745 726	A	0.8303 (0.7659-0.9)	6,19E-06
IE1A	All subjects	6	RS1633030	29 745 794	G	0.8224 (0.7595-0.8904)	1,42E-06
IE1A	All subjects	6	RS1633028	29 746 367	C	0.823 (0.7601-0.8911)	1,57E-06
IE1A	All subjects	6	RS2735042	29 747 373	G	0.8231 (0.7602-0.8912)	1,58E-06
IE1A	All subjects	6	RS1002046	29 754 016	A	0.8273 (0.7632-0.8968)	4,05E-06
IE1A	All subjects	6	RS1610641	29 759 066	G	0.834 (0.7714-0.9016)	5,00E-06
IE1A	All subjects	6	RS1610644	29 759 445	A	0.8277 (0.7636-0.8971)	4,24E-06
IE1A	All subjects	6	RS1611205	29 759 823	G	0.8308 (0.7683-0.8984)	3,39E-06
IE1A	All subjects	6	RS1610719	29 760 677	G	0.8336 (0.7711-0.9012)	4,72E-06
IE1A	All subjects	6	RS1610723	29 761 473	C	0.8308 (0.7685-0.8982)	3,17E-06
IE1A	All subjects	6	RS1610724	29 761 516	G	0.8316 (0.7692-0.899)	3,54E-06
IE1A	All subjects	6	RS1611220	29 761 835	G	0.8376 (0.7751-0.9051)	7,54E-06
IE1A	All subjects	6	RS1633011	29 762 693	A	0.828 (0.7638-0.8977)	4,70E-06
IE1A	All subjects	6	RS1633005	29 764 472	A	0.7729 (0.7013-0.8517)	2,02E-07
IE1A	All subjects	6	RS1633003	29 764 759	G	0.8395 (0.7768-0.9073)	9,99E-06
IE1A	All subjects	6	RS1630969	29 765 001	A	0.8291 (0.7636-0.9002)	8,08E-06
IE1A	All subjects	6	**RS1610649**	29 768 917	G	0.8037 (0.7437-0.8686)	**3,43E-08**
IE1A	All subjects	6	RS1610711	29 769 497	C	0.8266 (0.7626-0.8961)	3,73E-06
IE1A	All subjects	6	RS1610657	29 771 066	G	0.838 (0.7754-0.9056)	7,96E-06
IE1A	All subjects	6	RS1611192	29 771 879	G	0.8167 (0.7491-0.8904)	4,32E-06
IE1A	All subjects	6	RS1632988	29 772 395	A	0.8291 (0.7633-0.9005)	8,78E-06
IE1A	All subjects	6	RS1632987	29 772 549	G	0.8274 (0.7633-0.8969)	4,15E-06
IE1A	All subjects	6	RS1632983	29 773 509	A	0.8288 (0.7658-0.897)	3,25E-06
IE1A	All subjects	6	RS1736976	29 773 999	G	0.8316 (0.7693-0.8989)	3,46E-06
IE1A	All subjects	6	RS1736971	29 776 322	A	0.8282 (0.7641-0.8978)	4,60E-06
IE1A	All subjects	6	RS1736969	29 776 390	A	0.8236 (0.7597-0.8928)	2,47E-06
IE1A	All subjects	6	RS1610669	29 780 093	A	0.8376 (0.7751-0.9052)	7,62E-06
IE1A	All subjects	6	RS1736957	29 782 633	G	0.833 (0.7692-0.9022)	7,13E-06
IE1A	All subjects	6	RS1077432	29 782 868	G	0.8267 (0.7625-0.8962)	3,81E-06
IE1A	All subjects	6	RS1620173	29 785 149	C	0.8281 (0.7639-0.8976)	4,51E-06
IE1A	All subjects	6	RS1619379	29 785 235	A	0.8165 (0.7574-0.8802)	1,24E-07
IE1A	All subjects	6	**RS1610678**	29 789 190	G	0.7948 (0.7385-0.8555)	**9,49E-10**
IE1A	All subjects	6	**RS1611149**	29 789 999	A	0.7943 (0.7381-0.8549)	**7,93E-10**
IE1A	All subjects	6	**RS1736936**	29 794 317	A	0.7882 (0.7321-0.8485)	**2,54E-10**
IE1A	All subjects	6	**RS915669**	29 798 425	A	0.7938 (0.7376-0.8543)	**7,27E-10**
IE1A	All subjects	6	RS1611133	29 809 382	A	0.7953 (0.7286-0.8681)	2,95E-07
IE1A	All subjects	6	RS1611750	29 814 778	C	0.7921 (0.7256-0.8647)	1,89E-07
IE1A	All subjects	6	**RS2734985**	29 818 662	G	0.7811 (0.7147-0.8536)	**4,91E-08**

IE1A	All subjects	6	RS2247504	29 819 077	A	0.8177 (0.7589-0.8809)	1,19E-07
IE1A	All subjects	6	RS2523759	29 819 093	G	0.7853 (0.7187-0.8579)	8,60E-08
IE1A	All subjects	6	RS5013093	29 820 586	A	0.7849 (0.7184-0.8575)	8,00E-08
IE1A	All subjects	6	RS2523756	29 820 717	G	0.8152 (0.7568-0.8781)	7,15E-08
IE1A	All subjects	6	RS2734981	29 821 692	A	0.7877 (0.7209-0.8607)	1,31E-07
IE1A	All subjects	6	RS2734980	29 821 896	A	0.785 (0.7186-0.8577)	8,25E-08
IE1A	All subjects	6	RS2517862	29 821 937	A	0.7847 (0.7097-0.8676)	2,22E-06
IE1A	All subjects	6	RS2517861	29 821 982	A	0.785 (0.7185-0.8575)	8,00E-08
IE1A	All subjects	6	RS2517860	29 822 521	G	0.7841 (0.7178-0.8565)	6,72E-08
IE1A	All subjects	6	RS2428510	29 823 027	A	0.8154 (0.757-0.8783)	7,39E-08
IE1A	All subjects	6	RS2517855	29 823 164	A	0.7865 (0.7114-0.8695)	2,73E-06
IE1A	All subjects	6	**RS2517854**	29 823 296	G	0.8102 (0.752-0.8729)	**3,11E-08**
IE1A	All subjects	6	RS2844826	29 823 319	G	0.8156 (0.7572-0.8784)	7,47E-08
IE1A	All subjects	6	RS6919513	29 823 994	A	0.8166 (0.7581-0.8797)	9,49E-08
IE1A	All subjects	6	RS1611684	29 825 846	A	0.8156 (0.7572-0.8784)	7,47E-08
IE1A	All subjects	6	RS1611717	29 829 577	G	0.8158 (0.7574-0.8786)	7,69E-08
IE1A	All subjects	6	RS1611732	29 831 008	A	0.8149 (0.7566-0.8778)	6,68E-08
IE1A	All subjects	6	RS765649	29 831 058	A	0.8175 (0.7588-0.8807)	1,13E-07
IE1A	All subjects	6	**RS1611737**	29 831 571	G	0.8124 (0.7541-0.8752)	**4,54E-08**
IE1A	All subjects	6	RS1611738	29 831 706	A	0.7853 (0.7102-0.8684)	2,49E-06
IE1A	All subjects	6	RS1655900	29 916 618	A	0.7815 (0.7058-0.8653)	2,12E-06
IE1A	All subjects	6	RS2844806	29 933 439	A	0.8409 (0.7817-0.9046)	3,33E-06
IE1A	All subjects	6	RS3893464	29 935 250	A	0.8308 (0.772-0.8942)	7,60E-07
IE1A	All subjects	6	RS3132685	29 945 949	A	0.7704 (0.6893-0.861)	4,31E-06
IE1A	All subjects	6	RS8321	30 032 522	C	0.7291 (0.6478-0.8206)	1,61E-07
IE1A	All subjects	6	RS9261290	30 038 647	G	0.7287 (0.6474-0.8201)	1,53E-07
IE1A	All subjects	6	RS3132682	30 044 388	C	1.217 (1.131-1.31)	1,61E-07
IE1A	All subjects	6	RS7382061	30 047 965	A	1.219 (1.133-1.312)	1,32E-07
IE1A	All subjects	6	RS2057728	30 054 757	A	1.222 (1.135-1.316)	9,89E-08
IE1A	All subjects	6	RS6904455	30 055 377	G	1.22 (1.133-1.313)	1,35E-07
IE1A	All subjects	6	RS1264706	30 063 652	G	0.7309 (0.6495-0.8225)	1,97E-07
IE1A	All subjects	6	**RS9261394**	30 064 562	A	1.229 (1.142-1.323)	**4,05E-08**
IE1A	All subjects	6	RS1116222	30 071 279	C	0.8265 (0.7603-0.8985)	7,75E-06
IE1A	All subjects	6	RS2523989	30 078 275	A	0.7725 (0.701-0.8513)	1,92E-07
IE1A	All subjects	6	RS2239529	30 078 330	A	0.7791 (0.7069-0.8586)	4,77E-07
IE1A	All subjects	6	RS2523988	30 079 129	G	0.7697 (0.6963-0.8509)	3,09E-07
IE1A	All subjects	6	RS2249099	30 079 307	A	0.7703 (0.6993-0.8484)	1,21E-07
IE1A	All subjects	6	RS2523987	30 079 993	C	0.7705 (0.697-0.8518)	3,48E-07
IE1A	All subjects	6	RS2517598	30 080 274	A	0.7715 (0.7004-0.8498)	1,44E-07
IE1A	All subjects	6	RS2844793	30 080 496	A	0.7785 (0.7065-0.8578)	4,25E-07
IE1A	All subjects	6	RS2517597	30 081 189	A	0.7713 (0.6977-0.8526)	3,83E-07

IE1A	All subjects	6	RS2523986	30 081 246	A	0.7716 (0.698-0.853)	4,03E-07
IE1A	All subjects	6	RS2523985	30 081 334	G	0.7713 (0.7002-0.8495)	1,39E-07
IE1A	All subjects	6	RS2523984	30 082 003	A	0.7713 (0.7002-0.8495)	1,39E-07
IE1A	All subjects	6	RS2245420	30 082 688	C	0.7657 (0.695-0.8436)	6,68E-08
IE1A	All subjects	6	RS2523981	30 083 182	A	0.7732 (0.6996-0.8547)	4,81E-07
IE1A	All subjects	6	RS2523979	30 083 515	A	0.7899 (0.7157-0.8717)	2,75E-06
IE1A	All subjects	6	RS1362104	30 101 656	A	0.8261 (0.7666-0.8903)	5,67E-07
IE1A	All subjects	6	**RS2517645**	30 122 623	G	0.7547 (0.6821-0.835)	**4,93E-08**
IE1A	All subjects	6	RS929157	30 137 209	A	0.7474 (0.6714-0.832)	1,02E-07
IE1A	All subjects	6	RS2106072	30 153 363	A	0.7212 (0.6408-0.8117)	5,99E-08
IE1A	All subjects	6	RS2517617	30 161 536	A	0.7211 (0.6406-0.8116)	5,94E-08
IE1A	All subjects	6	RS2517614	30 163 955	A	0.7822 (0.7082-0.8641)	1,31E-06
IE1A	All subjects	6	RS2523722	30 165 273	A	0.7824 (0.7083-0.8643)	1,35E-06
IE1A	All subjects	6	RS2517613	30 165 662	A	0.7496 (0.6734-0.8346)	1,42E-07
IE1A	All subjects	6	RS2523721	30 166 266	A	0.7802 (0.7066-0.8615)	9,13E-07
IE1A	All subjects	6	RS2523719	30 168 319	A	0.7444 (0.6691-0.8281)	5,79E-08
IE1A	All subjects	6	RS2517612	30 169 092	G	0.7475 (0.6715-0.8321)	1,04E-07
IE1A	All subjects	6	RS2517611	30 169 327	G	0.7807 (0.7068-0.8624)	1,07E-06
IE1A	All subjects	6	RS2517610	30 170 280	G	0.7764 (0.7032-0.8572)	5,39E-07
IE1A	All subjects	6	RS1117490	30 170 510	G	0.777 (0.7038-0.8578)	5,80E-07
IE1A	All subjects	6	RS2844776	30 171 827	G	0.7761 (0.703-0.8569)	5,16E-07
IE1A	All subjects	6	RS971570	30 172 513	C	0.7733 (0.7003-0.8539)	3,75E-07
IE1A	All subjects	6	RS2021722	30 174 131	A	0.7754 (0.7023-0.856)	4,68E-07
IE1A	All subjects	6	RS885912	30 174 633	A	0.7768 (0.7036-0.8575)	5,58E-07
IE1A	All subjects	6	RS2188100	30 181 883	A	0.7483 (0.6722-0.833)	1,17E-07
IE1A	All subjects	6	RS885916	30 202 571	A	0.7469 (0.671-0.8314)	9,48E-08
IE1A	All subjects	6	RS3094078	30 224 970	T	0.7225 (0.6418-0.8133)	7,41E-08
IE1A	All subjects	6	**RS3094073**	30 231 224	A	0.7442 (0.6718-0.8243)	**1,48E-08**
IE1A	All subjects	6	**RS3130401**	30 231 273	G	0.7448 (0.6723-0.825)	**1,65E-08**
IE1A	All subjects	6	**RS3132659**	30 231 330	G	0.7456 (0.673-0.8259)	**1,86E-08**
IE1A	All subjects	6	**RS3132658**	30 231 666	G	0.7434 (0.6711-0.8236)	**1,36E-08**
IE1A	All subjects	6	**RS3094071**	30 231 768	A	0.7443 (0.6719-0.8245)	**1,54E-08**
IE1A	All subjects	6	**RS3130403**	30 232 009	C	0.7433 (0.671-0.8235)	**1,37E-08**
IE1A	All subjects	6	**RS3094630**	30 232 436	A	0.7448 (0.6724-0.8251)	**1,66E-08**
IE1A	All subjects	6	**RS3129702**	30 232 785	A	0.7448 (0.6724-0.8251)	**1,66E-08**
IE1A	All subjects	6	**RS3094629**	30 232 953	A	0.7448 (0.6723-0.8251)	**1,70E-08**
IE1A	All subjects	6	**RS3129703**	30 233 558	A	0.7443 (0.6719-0.8245)	**1,54E-08**
IE1A	All subjects	6	**RS3130405**	30 234 152	A	0.7447 (0.6723-0.825)	**1,64E-08**
IE1A	All subjects	6	**RS3129832**	30 234 657	G	0.739 (0.667-0.8189)	**7,64E-09**
IE1A	All subjects	6	**RS3129705**	30 234 721	A	0.7446 (0.6722-0.8248)	**1,60E-08**
IE1A	All subjects	6	RS3129830	30 248 711	A	0.7216 (0.6413-0.8119)	5,90E-08

IE1A	All subjects	6	**RS3130380**	30 279 130	A	0.7157 (0.6361-0.8053)	**2,71E-08**
IE1A	All subjects	6	**RS3094064**	30 296 253	A	0.7188 (0.6391-0.8084)	**3,64E-08**
IE1A	All subjects	6	RS3094067	30 299 245	C	0.7225 (0.6421-0.8129)	6,57E-08
IE1A	All subjects	6	**RS3129837**	30 306 306	G	0.7475 (0.6746-0.8282)	**2,71E-08**
IE1A	All subjects	6	**RS3129840**	30 307 342	G	0.7491 (0.676-0.8301)	**3,49E-08**
IE1A	All subjects	6	RS3132649	30 321 057	A	0.7343 (0.6549-0.8234)	1,24E-07
IE1A	All subjects	6	RS3094061	30 321 189	C	0.7343 (0.6549-0.8234)	1,23E-07
IE1A	All subjects	6	RS3130374	30 321 336	A	0.7343 (0.6549-0.8234)	1,25E-07
IE1A	All subjects	6	RS3130375	30 321 732	A	0.7359 (0.6562-0.8252)	1,55E-07
IE1A	All subjects	6	RS3130350	30 327 839	A	0.7242 (0.6441-0.8144)	7,02E-08
IE1A	All subjects	6	RS3094622	30 327 952	G	0.7333 (0.6538-0.8226)	1,21E-07
IE1A	All subjects	6	RS3130351	30 328 192	A	0.724 (0.6438-0.8141)	6,87E-08
IE1A	All subjects	6	**RS3130352**	30 328 357	A	0.7212 (0.6414-0.811)	**4,77E-08**
IE1A	All subjects	6	RS3094621	30 328 753	G	0.7347 (0.655-0.8242)	1,46E-07
IE1A	All subjects	6	RS3132647	30 330 737	G	0.7256 (0.6452-0.8161)	8,74E-08
IE1A	All subjects	6	RS3094054	30 333 505	A	0.7569 (0.675-0.8487)	1,86E-06
IE1A	All subjects	6	RS3129809	30 335 621	G	0.7413 (0.6612-0.8312)	2,96E-07
IE1A	All subjects	6	RS3129817	30 342 753	A	0.745 (0.6655-0.834)	3,19E-07
IE1A	All subjects	6	RS3129820	30 343 569	A	0.7412 (0.6611-0.831)	2,87E-07
IE1A	All subjects	6	RS3132631	30 344 645	A	0.7402 (0.6602-0.8299)	2,56E-07
IE1A	All subjects	6	RS3132630	30 345 118	A	0.7418 (0.6613-0.8322)	3,50E-07
IE1A	All subjects	6	RS3129822	30 346 208	A	0.7434 (0.6627-0.8339)	4,19E-07
IE1A	All subjects	6	RS3132625	30 347 720	G	0.7413 (0.6611-0.8311)	2,92E-07
IE1A	All subjects	6	RS3094032	30 351 547	A	0.7394 (0.6594-0.8291)	2,34E-07
IE1A	All subjects	6	RS3130126	30 353 739	A	0.7408 (0.6606-0.8306)	2,79E-07
IE1A	All subjects	6	RS3094050	30 358 591	G	0.7416 (0.6615-0.8314)	2,95E-07
IE1A	All subjects	6	RS3130116	30 365 448	G	0.7521 (0.6718-0.8421)	7,72E-07
IE1A	All subjects	6	RS3130141	30 432 177	A	0.7573 (0.6761-0.8482)	1,53E-06
IE1A	All subjects	6	RS3094694	30 451 904	G	0.7924 (0.7222-0.8695)	8,94E-07
IE1A	All subjects	6	RS3130117	30 508 956	A	0.7585 (0.6776-0.8491)	1,57E-06
IE1A	All subjects	6	RS3130247	30 515 043	G	0.7571 (0.6764-0.8474)	1,31E-06
IE1A	All subjects	6	RS3132610	30 544 401	G	0.7614 (0.6811-0.8511)	1,62E-06
IE1A	All subjects	6	RS9262132	30 611 350	A	0.7521 (0.6721-0.8416)	6,88E-07
IE1A	All subjects	6	RS9262135	30 618 906	G	0.7499 (0.6701-0.8392)	5,36E-07
IE1A	All subjects	6	RS9262141	30 644 137	G	0.7569 (0.6763-0.8471)	1,24E-06
IE1A	All subjects	6	RS9262142	30 650 026	A	0.7621 (0.6807-0.8531)	2,36E-06
IE1A	All subjects	6	RS9262143	30 652 781	A	0.7577 (0.6772-0.8478)	1,28E-06
IE1A	All subjects	6	RS3132585	30 687 614	G	0.7507 (0.6711-0.8398)	5,40E-07
IE1A	All subjects	6	RS3132583	30 688 575	C	0.75 (0.6704-0.839)	4,95E-07
IE1A	All subjects	6	RS3132582	30 689 001	T	0.7679 (0.6881-0.857)	2,39E-06
IE1A	All subjects	6	RS1059612	30 708 955	A	0.7573 (0.6767-0.8475)	1,29E-06

IE1A	All subjects	6	RS3129973	30 721 143	A	0.7413 (0.6628-0.829)	1,54E-07
IE1A	All subjects	6	RS3095336	30 738 446	A	0.7628 (0.6823-0.8527)	1,92E-06
IE1A	All subjects	6	RS3130673	30 746 519	A	0.7654 (0.6847-0.8557)	2,59E-06
IE1A	All subjects	6	RS3131050	30 760 025	G	0.7687 (0.6877-0.8593)	3,73E-06
IE1A	All subjects	6	RS3131060	30 763 291	A	0.7674 (0.6864-0.8578)	3,22E-06
IE1A	All subjects	6	RS3129986	30 763 562	A	0.7844 (0.7062-0.8712)	5,83E-06
IE1A	All subjects	6	RS3130641	30 764 081	A	0.7659 (0.6851-0.8562)	2,73E-06
IE1A	All subjects	6	RS1264377	30 764 907	A	0.7824 (0.7049-0.8684)	4,02E-06
IE1A	All subjects	6	RS1264376	30 765 579	A	0.7818 (0.7027-0.8697)	6,01E-06
IE1A	All subjects	6	**RS1264341**	30 802 465	G	0.727 (0.6496-0.8136)	**2,83E-08**
IE1A	All subjects	6	**RS2535340**	30 838 497	G	0.7261 (0.649-0.8125)	**2,37E-08**
IE1A	All subjects	6	**RS1264322**	30 857 894	A	0.728 (0.6506-0.8146)	**3,10E-08**
IE1A	All subjects	6	**RS886422**	30 864 279	A	0.7281 (0.6507-0.8147)	**3,14E-08**
IE1A	All subjects	6	**RS1049633**	30 867 527	A	0.7268 (0.6494-0.8134)	**2,76E-08**
IE1A	All subjects	6	**RS1264312**	30 872 982	A	0.7238 (0.6464-0.8105)	**2,11E-08**
IE1A	All subjects	6	**RS1264310**	30 873 605	A	0.7285 (0.6508-0.8156)	**3,77E-08**
IE1A	All subjects	6	**RS1264308**	30 879 987	A	0.7277 (0.6503-0.8142)	**2,95E-08**
IE1A	All subjects	6	**RS1264304**	30 882 415	A	0.726 (0.6488-0.8123)	**2,32E-08**
IE1A	All subjects	6	RS3131921	30 907 335	G	0.7544 (0.6782-0.8393)	2,18E-07
IE1A	All subjects	6	RS3130782	30 914 843	A	0.7571 (0.6802-0.8428)	3,59E-07
IE1A	All subjects	6	**RS3094086**	30 919 391	A	0.7371 (0.6605-0.8225)	**4,98E-08**
IE1A	All subjects	6	**RS3131934**	30 931 844	G	0.7382 (0.6641-0.8207)	**1,94E-08**
IE1A	All subjects	6	**RS3131783**	30 932 068	A	0.7346 (0.6607-0.8169)	**1,22E-08**
IE1A	All subjects	6	RS3132579	30 940 989	G	0.8026 (0.7295-0.8829)	6,30E-06
IE1A	All subjects	6	RS1634714	30 951 561	G	0.8029 (0.7298-0.8833)	6,57E-06
IE1A	All subjects	6	**RS1634721**	30 977 680	A	0.7114 (0.6361-0.7956)	**2,42E-09**
IE1A	All subjects	6	**RS3130544**	31 058 340	A	0.6939 (0.6209-0.7756)	**1,23E-10**
IE1A	All subjects	6	RS2233974	31 080 016	C	0.7656 (0.6941-0.8446)	9,64E-08
IE1A	All subjects	6	RS2233956	31 081 205	G	0.7896 (0.7169-0.8696)	1,60E-06
IE1A	All subjects	6	**RS3130557**	31 094 703	A	0.6862 (0.6138-0.7672)	**3,68E-11**
IE1A	All subjects	6	**RS7750641**	31 129 310	A	0.6882 (0.6157-0.7692)	**4,71E-11**
IE1A	All subjects	6	**RS3132510**	31 172 151	G	0.6642 (0.5911-0.7463)	**6,01E-12**
IE1A	All subjects	6	RS9263911	31 175 667	G	1.181 (1.097-1.27)	9,14E-06
IE1A	All subjects	6	RS3132505	31 177 503	A	0.8189 (0.7497-0.8945)	9,16E-06
IE1A	All subjects	6	RS2394895	31 206 979	G	0.817 (0.748-0.8925)	7,31E-06
IE1A	All subjects	6	RS2394944	31 220 450	A	0.8062 (0.7369-0.882)	2,61E-06
IE1A	All subjects	6	RS1793891	31 221 698	A	0.8105 (0.7433-0.8838)	1,96E-06
IE1A	All subjects	6	RS1986997	31 228 410	A	0.847 (0.7868-0.9118)	9,97E-06
IE1A	All subjects	6	RS2245822	31 230 800	A	0.8087 (0.7407-0.8831)	2,23E-06
IE1A	All subjects	6	RS1049281	31 236 567	A	0.8232 (0.7636-0.8874)	3,88E-07
IE1A	All subjects	6	**RS2844613**	31 243 846	A	0.7359 (0.6645-0.815)	**3,93E-09**

IE1A	All subjects	6	RS3130696	31 243 884	A	0.8002 (0.7319-0.8748)	9,67E-07
IE1A	All subjects	6	RS2524074	31 244 021	G	0.8119 (0.7521-0.8763)	9,03E-08
IE1A	All subjects	6	RS2844603	31 250 854	A	0.8402 (0.7804-0.9047)	3,95E-06
IE1A	All subjects	6	RS2853933	31 254 088	A	0.8443 (0.7842-0.909)	7,01E-06
IE1A	All subjects	6	RS2524040	31 257 625	A	0.8391 (0.7793-0.9035)	3,32E-06
IE1A	All subjects	6	RS2524163	31 259 579	G	0.8438 (0.7837-0.9085)	6,58E-06
IE1A	All subjects	6	RS2524156	31 260 397	A	0.8441 (0.7839-0.9088)	6,98E-06
IE1A	All subjects	6	RS2243868	31 261 276	A	0.8431 (0.7831-0.9078)	5,99E-06
IE1A	All subjects	6	**RS2247056**	31 265 490	A	0.7866 (0.7273-0.8508)	**1,95E-09**
IE1A	All subjects	6	RS2853922	31 266 190	A	0.8407 (0.7807-0.9052)	4,28E-06
IE1A	All subjects	6	RS2524089	31 266 522	C	0.8395 (0.7796-0.9039)	3,52E-06
IE1A	All subjects	6	RS2524066	31 269 154	A	0.8398 (0.78-0.9042)	3,62E-06
IE1A	All subjects	6	**RS2596487**	31 325 056	A	0.7058 (0.6385-0.7801)	**9,07E-12**
IE1A	All subjects	6	**RS2523573**	31 328 988	C	0.6463 (0.5772-0.7237)	**3,86E-14**
IE1A	All subjects	6	RS2844575	31 334 945	G	1.191 (1.107-1.281)	2,66E-06
IE1A	All subjects	6	RS3094600	31 347 144	A	0.8221 (0.7555-0.8946)	5,50E-06
IE1A	All subjects	6	**RS9266669**	31 348 077	A	0.6732 (0.6056-0.7484)	**2,37E-13**
IE1A	All subjects	6	RS3094604	31 434 111	G	0.8006 (0.7267-0.882)	6,74E-06
IE1A	All subjects	6	**RS3094013**	31 434 366	A	0.6485 (0.5793-0.726)	**5,65E-14**
IE1A	All subjects	6	**RS3131618**	31 434 621	G	0.6468 (0.5773-0.7246)	**5,68E-14**
IE1A	All subjects	6	RS2518028	31 436 047	A	0.8337 (0.7733-0.8987)	2,08E-06
IE1A	All subjects	6	RS1055569	31 440 082	A	1.205 (1.11-1.308)	7,86E-06
IE1A	All subjects	6	RS3828886	31 440 552	C	1.235 (1.134-1.346)	1,38E-06
IE1A	All subjects	6	**RS3131643**	31 442 782	A	0.6932 (0.6259-0.7677)	**2,02E-12**
IE1A	All subjects	6	**RS3099844**	31 448 976	A	0.636 (0.5683-0.7118)	**3,38E-15**
IE1A	All subjects	6	RS2523706	31 451 567	G	0.8361 (0.7737-0.9037)	6,29E-06
IE1A	All subjects	6	**RS3094011**	31 451 836	G	0.6345 (0.5667-0.7104)	**3,02E-15**
IE1A	All subjects	6	RS2516504	31 452 475	A	0.839 (0.7763-0.9067)	9,37E-06
IE1A	All subjects	6	RS2516408	31 463 491	A	0.8349 (0.7729-0.902)	4,68E-06
IE1A	All subjects	6	**RS2855812**	31 472 720	A	0.7856 (0.7236-0.8531)	**9,28E-09**
IE1A	All subjects	6	RS2516400	31 481 105	A	0.8346 (0.7725-0.9016)	4,44E-06
IE1A	All subjects	6	**RS9267445**	31 483 481	C	0.6446 (0.5749-0.7228)	**5,43E-14**
IE1A	All subjects	6	**RS3093988**	31 492 453	A	0.7487 (0.6781-0.8267)	**1,03E-08**
IE1A	All subjects	6	RS2734573	31 494 738	A	0.8419 (0.7817-0.9067)	5,48E-06
IE1A	All subjects	6	RS2516482	31 496 569	G	0.7624 (0.6907-0.8414)	7,12E-08
IE1A	All subjects	6	**RS2734583**	31 505 480	G	0.6634 (0.5927-0.7427)	**1,02E-12**
IE1A	All subjects	6	RS3093974	31 506 210	A	0.8236 (0.7626-0.8895)	7,80E-07
IE1A	All subjects	6	RS2071592	31 515 340	A	0.8231 (0.7608-0.8906)	1,28E-06
IE1A	All subjects	6	RS909253	31 540 313	G	0.8398 (0.7777-0.907)	8,63E-06
IE1A	All subjects	6	RS1041981	31 540 784	A	0.8366 (0.7746-0.9035)	5,55E-06
IE1A	All subjects	6	RS1800629	31 543 031	A	0.7667 (0.6948-0.846)	1,22E-07

IE1A	All subjects	6	RS2857595	31 568 469	A	0.8081 (0.7377-0.8852)	4,58E-06
IE1A	All subjects	6	RS3132451	31 582 025	C	0.7966 (0.7257-0.8745)	1,76E-06
IE1A	All subjects	6	RS3130070	31 591 808	G	0.7811 (0.711-0.8581)	2,63E-07
IE1A	All subjects	6	RS3130622	31 592 524	C	0.7862 (0.7155-0.864)	5,75E-07
IE1A	All subjects	6	RS3130623	31 597 700	A	0.7987 (0.7285-0.8757)	1,71E-06
IE1A	All subjects	6	RS3130626	31 598 489	G	0.7818 (0.7115-0.859)	3,02E-07
IE1A	All subjects	6	RS2736157	31 600 820	G	0.7841 (0.7137-0.8614)	4,02E-07
IE1A	All subjects	6	RS3115663	31 601 843	G	0.7871 (0.7153-0.866)	9,17E-07
IE1A	All subjects	6	RS9267522	31 603 770	G	0.7805 (0.7104-0.8575)	2,45E-07
IE1A	All subjects	6	RS10885	31 604 591	A	0.7828 (0.7124-0.8602)	3,54E-07
IE1A	All subjects	6	RS3130628	31 609 272	G	0.7821 (0.7118-0.8593)	3,13E-07
IE1A	All subjects	6	RS3130048	31 613 739	G	0.8186 (0.752-0.8911)	3,82E-06
IE1A	All subjects	6	RS3117583	31 619 576	G	0.7798 (0.7098-0.8568)	2,25E-07
IE1A	All subjects	6	**RS3117582**	31 620 520	C	0.6623 (0.5902-0.7431)	**2,35E-12**
IE1A	All subjects	6	**RS805262**	31 628 733	A	0.7764 (0.7213-0.8358)	**1,65E-11**
IE1A	All subjects	6	RS3130618	31 632 134	A	0.7807 (0.7105-0.8579)	2,68E-07
IE1A	All subjects	6	**RS9267531**	31 636 742	G	0.6629 (0.5907-0.7439)	**2,76E-12**
IE1A	All subjects	6	**RS3101018**	31 705 864	A	0.6599 (0.5875-0.7411)	**2,27E-12**
IE1A	All subjects	6	**RS3132445**	31 712 196	A	0.6645 (0.5922-0.7457)	**3,65E-12**
IE1A	All subjects	6	**RS3130484**	31 715 882	G	0.6632 (0.591-0.7442)	**2,83E-12**
IE1A	All subjects	6	**RS3131379**	31 721 033	A	0.6661 (0.5935-0.7476)	**5,21E-12**
IE1A	All subjects	6	**RS3117574**	31 725 230	A	0.6648 (0.5924-0.746)	**3,83E-12**
IE1A	All subjects	6	**RS3131378**	31 725 285	G	0.6623 (0.5902-0.7432)	**2,42E-12**
IE1A	All subjects	6	**RS3117577**	31 727 474	G	0.6625 (0.5904-0.7435)	**2,54E-12**
IE1A	All subjects	6	**RS3101017**	31 733 466	G	0.6646 (0.5923-0.7457)	**3,55E-12**
IE1A	All subjects	6	RS2075798	31 846 741	A	1.378 (1.206-1.575)	2,52E-06
IE1A	All subjects	6	RS555007	31 850 332	G	1.357 (1.189-1.55)	6,56E-06
IE1A	All subjects	6	**RS652888**	31 851 234	G	0.7644 (0.6975-0.8376)	**8,69E-09**
IE1A	All subjects	6	**RS558702**	31 870 326	A	0.6602 (0.5882-0.7409)	**1,79E-12**
IE1A	All subjects	6	**RS519417**	31 878 433	A	0.6561 (0.5844-0.7365)	**9,34E-13**
IE1A	All subjects	6	RS511294	31 888 869	C	1.541 (1.302-1.823)	4,90E-07
IE1A	All subjects	6	**RS497309**	31 892 484	C	0.6572 (0.5857-0.7375)	**9,47E-13**
IE1A	All subjects	6	RS498240	31 892 592	A	1.54 (1.301-1.823)	5,29E-07
IE1A	All subjects	6	RS2257331	31 898 285	G	1.539 (1.3-1.821)	5,25E-07
IE1A	All subjects	6	RS9332735	31 901 773	A	1.764 (1.412-2.203)	5,72E-07
IE1A	All subjects	6	RS635348	31 902 289	C	1.525 (1.289-1.805)	9,11E-07
IE1A	All subjects	6	RS621701	31 903 121	A	1.527 (1.29-1.807)	8,26E-07
IE1A	All subjects	6	**RS1042663**	31 905 130	A	1.471 (1.283-1.686)	**2,96E-08**
IE1A	All subjects	6	**RS2507953**	31 905 328	A	1.472 (1.284-1.687)	**2,81E-08**
IE1A	All subjects	6	**RS550605**	31 907 147	G	1.466 (1.279-1.681)	**3,89E-08**
IE1A	All subjects	6	**RS653414**	31 907 168	A	1.471 (1.283-1.686)	**2,95E-08**

IE1A	All subjects	6	**RS497239**	31 908 761	G	1.468 (1.281-1.682)	**3,39E-08**
IE1A	All subjects	6	**RS609061**	31 910 162	A	1.469 (1.281-1.683)	**3,29E-08**
IE1A	All subjects	6	**RS2242572**	31 910 929	A	1.47 (1.283-1.685)	**3,06E-08**
IE1A	All subjects	6	**RS547154**	31 910 938	A	1.469 (1.282-1.684)	**3,17E-08**
IE1A	All subjects	6	**RS541862**	31 916 951	G	1.466 (1.279-1.68)	**3,96E-08**
IE1A	All subjects	6	**RS1270942**	31 918 860	G	0.6563 (0.5849-0.7366)	**8,23E-13**
IE1A	All subjects	6	RS522162	31 919 917	G	1.455 (1.269-1.667)	6,95E-08
IE1A	All subjects	6	**RS760070**	31 919 956	G	1.465 (1.278-1.679)	**4,23E-08**
IE1A	All subjects	6	**RS550513**	31 920 687	A	1.466 (1.279-1.68)	**3,92E-08**
IE1A	All subjects	6	**RS403569**	31 924 880	A	1.465 (1.278-1.679)	**4,17E-08**
IE1A	All subjects	6	**RS438999**	31 928 306	G	1.459 (1.274-1.672)	**5,00E-08**
IE1A	All subjects	6	RS429608	31 930 462	A	1.297 (1.164-1.446)	2,49E-06
IE1A	All subjects	6	RS444921	31 932 177	A	1.314 (1.167-1.479)	6,33E-06
IE1A	All subjects	6	RS406936	31 933 161	A	1.313 (1.167-1.479)	6,58E-06
IE1A	All subjects	6	RS492899	31 933 518	G	1.41 (1.218-1.632)	4,26E-06
IE1A	All subjects	6	RS454212	31 934 372	A	1.315 (1.168-1.48)	6,02E-06
IE1A	All subjects	6	RS453821	31 935 311	A	1.312 (1.165-1.477)	7,08E-06
IE1A	All subjects	6	RS449643	31 936 679	A	1.315 (1.168-1.48)	6,08E-06
IE1A	All subjects	6	RS387608	31 941 557	A	1.312 (1.165-1.477)	7,05E-06
IE1A	All subjects	6	RS6455	32 006 896	G	1.699 (1.356-2.127)	3,94E-06
IE1A	All subjects	6	RS12333245	32 019 769	A	1.346 (1.185-1.53)	5,06E-06
IE1A	All subjects	6	RS11969759	32 021 130	A	1.447 (1.234-1.698)	5,81E-06
IE1A	All subjects	6	RS2269429	32 029 183	A	1.34 (1.179-1.523)	7,74E-06
IE1A	All subjects	6	**RS1150755**	32 038 550	A	0.7034 (0.6358-0.7782)	**8,87E-12**
IE1A	All subjects	6	RS7774197	32 046 275	C	1.449 (1.235-1.699)	5,31E-06
IE1A	All subjects	6	**RS1150754**	32 050 758	A	0.7022 (0.6347-0.7768)	**7,02E-12**
IE1A	All subjects	6	RS169496	32 052 983	A	1.341 (1.181-1.524)	6,45E-06
IE1A	All subjects	6	RS204900	32 056 580	C	1.343 (1.182-1.526)	5,94E-06
IE1A	All subjects	6	RS204899	32 057 627	A	1.348 (1.185-1.533)	5,44E-06
IE1A	All subjects	6	**RS1150753**	32 059 867	G	0.6601 (0.5882-0.7408)	**1,66E-12**
IE1A	All subjects	6	**RS1150752**	32 064 726	G	0.6521 (0.5806-0.7323)	**5,19E-13**
IE1A	All subjects	6	RS439844	32 072 940	A	1.342 (1.181-1.525)	6,38E-06
IE1A	All subjects	6	RS411337	32 077 380	A	1.416 (1.238-1.62)	3,75E-07
IE1A	All subjects	6	**RS1269852**	32 080 191	C	0.6684 (0.5957-0.75)	**7,18E-12**
IE1A	All subjects	6	RS9469084	32 080 383	A	1.407 (1.23-1.609)	6,35E-07
IE1A	All subjects	6	RS204888	32 089 142	A	1.407 (1.231-1.61)	6,01E-07
IE1A	All subjects	6	**RS3130288**	32 096 001	A	0.6665 (0.594-0.7478)	**4,96E-12**
IE1A	All subjects	6	RS169494	32 097 876	A	1.427 (1.247-1.632)	2,27E-07
IE1A	All subjects	6	**RS3134608**	32 117 971	C	0.7463 (0.6787-0.8207)	**1,55E-09**
IE1A	All subjects	6	RS1053924	32 120 715	A	1.225 (1.134-1.323)	2,27E-07
IE1A	All subjects	6	RS3134950	32 127 477	C	1.223 (1.135-1.318)	1,33E-07

IE1A	All subjects	6	**RS3096697**	32 134 510	A	0.7444 (0.6769-0.8186)	**1,17E-09**
IE1A	All subjects	6	**RS3130347**	32 134 656	G	0.7465 (0.6788-0.8208)	**1,60E-09**
IE1A	All subjects	6	RS1061808	32 136 547	A	1.222 (1.134-1.317)	1,43E-07
IE1A	All subjects	6	**RS3130284**	32 140 487	G	0.7456 (0.678-0.8199)	**1,41E-09**
IE1A	All subjects	6	RS408359	32 141 883	A	1.436 (1.231-1.675)	4,15E-06
IE1A	All subjects	6	**RS3134947**	32 145 205	A	0.7465 (0.6788-0.8209)	**1,63E-09**
IE1A	All subjects	6	RS2269423	32 145 707	A	1.216 (1.129-1.31)	2,65E-07
IE1A	All subjects	6	**RS3134945**	32 146 492	A	0.7465 (0.6788-0.8209)	**1,66E-09**
IE1A	All subjects	6	**RS3130349**	32 147 696	A	0.7183 (0.6505-0.7931)	**5,97E-11**
IE1A	All subjects	6	**RS1800625**	32 152 442	G	0.7189 (0.6511-0.7937)	**6,51E-11**
IE1A	All subjects	6	RS204995	32 154 285	G	0.7736 (0.7038-0.8503)	1,03E-07
IE1A	All subjects	6	**RS204993**	32 155 581	G	0.7503 (0.6862-0.8203)	**2,85E-10**
IE1A	All subjects	6	**RS204992**	32 156 908	A	0.7637 (0.6944-0.8399)	**2,79E-08**
IE1A	All subjects	6	**RS176095**	32 158 319	G	0.7641 (0.6946-0.8407)	**3,30E-08**
IE1A	All subjects	6	RS204991	32 161 366	G	0.7577 (0.6854-0.8376)	5,77E-08
IE1A	All subjects	6	**RS204990**	32 161 430	A	0.7506 (0.6785-0.8304)	**2,62E-08**
IE1A	All subjects	6	**RS2071278**	32 165 444	G	0.709 (0.6375-0.7885)	**2,27E-10**
IE1A	All subjects	6	**RS3134942**	32 168 771	A	0.6734 (0.6029-0.752)	**2,28E-12**
IE1A	All subjects	6	**RS2071287**	32 170 433	A	0.7813 (0.7254-0.8416)	**7,37E-11**
IE1A	All subjects	6	**RS3132935**	32 171 075	G	0.758 (0.6885-0.8346)	**1,66E-08**
IE1A	All subjects	6	**RS2071277**	32 171 683	G	0.7775 (0.722-0.8372)	**2,58E-11**
IE1A	All subjects	6	**RS3131296**	32 172 993	A	0.6716 (0.601-0.7504)	**2,08E-12**
IE1A	All subjects	6	**RS206017**	32 176 211	G	1.286 (1.194-1.385)	**3,03E-11**
IE1A	All subjects	6	**RS206018**	32 177 880	C	1.323 (1.203-1.456)	**8,98E-09**
IE1A	All subjects	6	**RS3132956**	32 179 438	A	0.6741 (0.6036-0.7528)	**2,59E-12**
IE1A	All subjects	6	**RS3131290**	32 183 175	A	0.742 (0.6871-0.8013)	**2,89E-14**
IE1A	All subjects	6	**RS3134799**	32 184 221	A	0.7529 (0.6984-0.8116)	**1,30E-13**
IE1A	All subjects	6	**RS436388**	32 186 264	A	1.296 (1.203-1.396)	**7,20E-12**
IE1A	All subjects	6	**RS379464**	32 186 348	A	1.677 (1.404-2.005)	**1,26E-08**
IE1A	All subjects	6	**RS394657**	32 187 023	G	0.757 (0.7023-0.8159)	**3,49E-13**
IE1A	All subjects	6	**RS1109771**	32 187 605	G	0.7815 (0.7257-0.8416)	**6,99E-11**
IE1A	All subjects	6	**RS8192584**	32 189 192	A	1.87 (1.513-2.312)	**6,86E-09**
IE1A	All subjects	6	RS715299	32 189 841	C	0.8144 (0.7481-0.8866)	2,15E-06
IE1A	All subjects	6	RS915895	32 190 217	G	0.8177 (0.7514-0.8898)	3,07E-06
IE1A	All subjects	6	**RS915894**	32 190 390	C	0.7322 (0.6765-0.7924)	**1,13E-14**
IE1A	All subjects	6	RS443198	32 190 406	G	0.8129 (0.7488-0.8826)	7,79E-07
IE1A	All subjects	6	**RS3134930**	32 191 620	A	0.7583 (0.6917-0.8313)	**3,64E-09**
IE1A	All subjects	6	RS377763	32 199 144	A	0.7955 (0.7253-0.8726)	1,23E-06
IE1A	All subjects	6	**RS6901158**	32 205 942	A	0.675 (0.6069-0.7508)	**4,45E-13**
IE1A	All subjects	6	**RS424232**	32 208 324	A	0.77 (0.706-0.8398)	**3,52E-09**
IE1A	All subjects	6	**RS3132971**	32 230 256	C	0.6381 (0.5682-0.7165)	**3,08E-14**

IE1A	All subjects	6	**RS7775397**	32 261 252	C	0.6425 (0.5723-0.7213)	**6,56E-14**
IE1A	All subjects	6	**RS9268219**	32 284 108	C	0.6632 (0.5892-0.7463)	**9,56E-12**
IE1A	All subjects	6	**RS9268235**	32 290 208	A	0.6438 (0.5734-0.7228)	**9,15E-14**
IE1A	All subjects	6	RS1265762	32 321 115	C	1.188 (1.103-1.28)	5,63E-06
IE1A	All subjects	6	RS1265760	32 321 872	A	1.19 (1.105-1.282)	4,37E-06
IE1A	All subjects	6	RS1265759	32 322 393	G	1.187 (1.102-1.279)	6,78E-06
IE1A	All subjects	6	RS6904636	32 327 781	G	1.183 (1.098-1.275)	9,44E-06
IE1A	All subjects	6	RS3129911	32 328 403	G	1.187 (1.102-1.279)	6,38E-06
IE1A	All subjects	6	RS3129922	32 333 098	G	1.192 (1.106-1.284)	3,87E-06
IE1A	All subjects	6	**RS2273017**	32 337 630	G	1.234 (1.145-1.331)	**4,36E-08**
IE1A	All subjects	6	**RS2073045**	32 339 548	A	0.7661 (0.7083-0.8286)	**2,80E-11**
IE1A	All subjects	6	**RS7758128**	32 345 283	A	1.813 (1.476-2.226)	**1,35E-08**
IE1A	All subjects	6	**RS2894254**	32 345 689	C	0.6436 (0.5728-0.7231)	**1,21E-13**
IE1A	All subjects	6	RS17423649	32 357 133	A	1.299 (1.17-1.442)	9,23E-07
IE1A	All subjects	6	RS17202259	32 357 489	C	1.298 (1.169-1.442)	9,88E-07
IE1A	All subjects	6	RS3117099	32 358 270	A	0.8188 (0.7495-0.8944)	9,28E-06
IE1A	All subjects	6	RS16870123	32 359 460	A	1.293 (1.165-1.435)	1,35E-06
IE1A	All subjects	6	RS3817969	32 361 388	A	1.281 (1.155-1.421)	2,86E-06
IE1A	All subjects	6	RS1980493	32 363 215	G	0.794 (0.7194-0.8763)	4,59E-06
IE1A	All subjects	6	RS2227138	32 384 500	A	0.7787 (0.7042-0.8612)	1,11E-06
IE1A	All subjects	6	RS3135353	32 392 877	A	0.7766 (0.7022-0.8589)	8,58E-07
IE1A	All subjects	6	**RS3129843**	32 395 726	G	0.6484 (0.5772-0.7285)	**3,01E-13**
IE1A	All subjects	6	**RS3763326**	32 413 557	G	1.814 (1.483-2.218)	**6,62E-09**
IE1A	All subjects	6	**RS9268877**	32 431 147	A	1.234 (1.145-1.331)	**4,48E-08**
IE1A	All subjects	6	RS9268979	32 435 044	A	1.23 (1.141-1.328)	8,55E-08
IE1A	All subjects	6	**RS9270665**	32 566 232	A	0.734 (0.6679-0.8067)	**1,34E-10**
IE1A	All subjects	6	**RS642093**	32 582 075	A	0.7452 (0.6798-0.817)	**3,71E-10**
IE1A	All subjects	6	**RS3129763**	32 590 925	A	0.7349 (0.6689-0.8075)	**1,49E-10**
IE1A	All subjects	6	**RS9272219**	32 602 269	A	0.7481 (0.6871-0.8146)	**2,39E-11**
IE1A	All subjects	6	RS3104369	32 602 482	A	1.205 (1.116-1.301)	2,06E-06
IE1A	All subjects	6	**RS2187668**	32 605 884	A	0.7017 (0.6285-0.7836)	**3,08E-10**
IE1A	All subjects	6	**RS9273012**	32 611 641	G	0.7461 (0.6853-0.8123)	**1,48E-11**
IE1A	All subjects	6	**RS17843604**	32 620 283	A	0.7302 (0.6761-0.7885)	**1,05E-15**
IE1A	All subjects	6	**RS9273327**	32 623 223	C	0.7015 (0.6278-0.7839)	**3,94E-10**
IE1A	All subjects	6	**RS9273349**	32 625 869	G	0.7042 (0.652-0.7606)	**4,70E-19**
IE1A	All subjects	6	RS6928482	32 626 249	G	0.833 (0.7697-0.9016)	6,04E-06
IE1A	All subjects	6	**RS9273363**	32 626 272	A	0.7238 (0.667-0.7855)	**9,43E-15**
IE1A	All subjects	6	**RS6906021**	32 626 311	G	0.7653 (0.7074-0.828)	**2,75E-11**
IE1A	All subjects	6	**RS1063355**	32 627 714	C	0.7038 (0.6516-0.7602)	**4,40E-19**
IE1A	All subjects	6	**RS9273448**	32 627 747	A	1.327 (1.227-1.435)	**1,66E-12**
IE1A	All subjects	6	**RS2854275**	32 628 428	A	0.6983 (0.6255-0.7797)	**1,69E-10**

IE1A	All subjects	6	RS2647025	32 635 949	A	0.8251 (0.758-0.8981)	8,93E-06
IE1A	All subjects	6	RS9275141	32 651 117	C	0.8158 (0.7544-0.8823)	3,46E-07
IE1A	All subjects	6	RS2856695	32 651 894	G	0.8165 (0.755-0.883)	3,84E-07
IE1A	All subjects	6	RS3021058	32 652 359	C	0.8145 (0.7532-0.8808)	2,78E-07
IE1A	All subjects	6	RS4947342	32 653 070	A	0.8075 (0.7389-0.8825)	2,36E-06
IE1A	All subjects	6	RS2856692	32 653 385	C	0.8044 (0.736-0.8792)	1,60E-06
IE1A	All subjects	6	RS4642516	32 657 543	A	0.8128 (0.7516-0.879)	2,13E-07
IE1A	All subjects	6	RS5000634	32 663 564	G	0.8188 (0.7557-0.8872)	1,03E-06
IE1A	All subjects	6	RS6457622	32 664 163	C	0.8163 (0.7535-0.8844)	6,83E-07
IE1A	All subjects	6	RS9275312	32 665 728	G	0.8021 (0.7282-0.8835)	7,78E-06
IE1A	All subjects	6	**RS1794282**	32 666 526	A	0.6492 (0.5777-0.7295)	**3,85E-13**
IE1A	All subjects	6	RS9275328	32 666 822	A	0.8035 (0.7294-0.8851)	9,21E-06
IE1A	All subjects	6	RS9275330	32 666 875	G	0.8037 (0.7296-0.8853)	9,51E-06
IE1A	All subjects	6	RS9275333	32 666 968	G	0.8018 (0.7279-0.8831)	7,46E-06
IE1A	All subjects	6	**RS3135006**	32 667 119	A	1.33 (1.229-1.439)	**1,40E-12**
IE1A	All subjects	6	**RS4947344**	32 677 846	A	1.283 (1.189-1.384)	**1,23E-10**
IE1A	All subjects	6	RS9275601	32 682 664	A	0.8145 (0.755-0.8786)	1,13E-07
IE1A	All subjects	6	RS3873444	32 682 724	A	1.367 (1.202-1.555)	2,04E-06
IE1A	All subjects	6	RS2857193	32 747 528	G	1.188 (1.102-1.281)	6,69E-06
IE1A	All subjects	6	RS28986337	32 748 398	A	1.381 (1.206-1.582)	3,02E-06
IE1A	All subjects	6	RS28986338	32 748 810	A	1.386 (1.211-1.587)	2,33E-06
IE1A	All subjects	6	RS28986359	32 751 349	C	1.379 (1.204-1.58)	3,64E-06
IE1A	All subjects	6	RS28986364	32 751 691	C	1.383 (1.207-1.585)	2,97E-06
IE1A	All subjects	6	**RS9276689**	32 751 962	A	0.6444 (0.57-0.7285)	**2,19E-12**
IE1A	All subjects	6	RS28986373	32 752 150	A	1.383 (1.208-1.584)	2,71E-06
IE1A	All subjects	6	RS13204139	32 752 685	A	1.374 (1.2-1.573)	4,33E-06
IE1A	All subjects	6	RS2857177	32 752 840	A	1.187 (1.102-1.28)	7,31E-06
IE1A	All subjects	6	RS28986396	32 754 430	A	1.379 (1.205-1.58)	3,25E-06
IE1A	All subjects	6	**RS7762279**	32 755 290	G	0.6429 (0.5689-0.7265)	**1,41E-12**
IE1A	All subjects	6	RS2857165	32 757 737	G	1.19 (1.104-1.283)	5,46E-06
IE1A	All subjects	6	RS2857164	32 758 219	C	1.191 (1.105-1.284)	4,64E-06
IE1A	All subjects	6	RS1158785	32 758 571	A	1.193 (1.107-1.286)	4,09E-06
IE1A	All subjects	6	RS2621384	32 759 273	G	1.192 (1.106-1.284)	4,61E-06
IE1A	All subjects	6	RS2857161	32 759 297	G	1.194 (1.108-1.287)	3,53E-06
IE1A	All subjects	6	RS9276722	32 762 391	G	1.381 (1.207-1.581)	2,86E-06
IE1A	All subjects	6	RS9276726	32 763 888	G	0.8219 (0.7602-0.8887)	8,53E-07
IE1A	All subjects	6	RS1383261	32 765 451	A	0.8353 (0.7728-0.9029)	5,78E-06
IE1A	All subjects	6	RS6932969	32 765 671	G	0.833 (0.7708-0.9003)	3,99E-06
IE1A	All subjects	6	RS7383606	32 766 593	A	0.8341 (0.7718-0.9014)	4,61E-06
IE1A	All subjects	6	RS9276734	32 766 854	A	0.8336 (0.7714-0.9009)	4,36E-06
IE1A	All subjects	6	RS7382714	32 767 496	G	0.8339 (0.7716-0.9012)	4,51E-06

IE1A	All subjects	6	**RS4947350**	32 767 620	G	0.7622 (0.6978-0.8325)	**1,61E-09**
IE1A	All subjects	6	RS7381376	32 767 673	G	0.8346 (0.7721-0.9021)	5,26E-06
IE1A	All subjects	6	RS6899857	32 770 482	G	0.8345 (0.7723-0.9018)	4,82E-06
IE1A	All subjects	6	RS6912414	32 774 465	A	0.8218 (0.7602-0.8885)	8,14E-07
IE1A	All subjects	6	RS7382619	32 775 809	A	0.8348 (0.7725-0.9022)	5,16E-06
IE1A	All subjects	6	RS7382649	32 776 087	A	0.8343 (0.772-0.9016)	4,76E-06
IE1A	All subjects	6	**RS11244**	32 780 724	A	0.782 (0.7192-0.8503)	**8,77E-09**
IE1A	All subjects	6	RS13209654	32 792 659	G	1.389 (1.213-1.591)	2,03E-06
IE1A	All subjects	6	RS1015166	32 798 731	A	0.8312 (0.7696-0.8977)	2,52E-06
IE1A	All subjects	6	RS3819714	32 804 217	A	1.238 (1.144-1.34)	1,20E-07
IE1A	All subjects	6	RS3819715	32 804 219	A	1.235 (1.141-1.336)	1,62E-07
IE1A	All subjects	6	RS241427	32 804 414	A	1.228 (1.14-1.323)	7,26E-08
IE1A	All subjects	6	RS241426	32 804 553	A	1.226 (1.139-1.321)	7,09E-08
IE1A	All subjects	6	**RS3819720**	32 804 570	A	0.748 (0.6912-0.8096)	**6,23E-13**
IE1A	All subjects	6	**RS241425**	32 804 909	A	1.273 (1.183-1.371)	**1,43E-10**
IE1A	All subjects	6	**RS241424**	32 804 934	A	0.8129 (0.7549-0.8753)	**4,06E-08**
IE1A	All subjects	6	**RS2239701**	32 805 049	G	0.7802 (0.7239-0.8409)	**8,37E-11**
IE1A	All subjects	6	RS2071465	32 805 470	C	0.8217 (0.7617-0.8864)	3,88E-07
IE1A	All subjects	6	RS4148870	32 806 391	A	0.839 (0.7785-0.9043)	4,40E-06
IE1A	All subjects	6	RS2071552	32 806 461	G	0.8352 (0.775-0.9001)	2,42E-06
IE1A	All subjects	6	RS4148868	32 806 584	A	0.8345 (0.7742-0.8995)	2,28E-06
IE1A	All subjects	6	RS4713598	32 806 786	C	0.8351 (0.7744-0.9006)	2,86E-06
IE1A	All subjects	6	RS3763366	32 807 446	C	0.8316 (0.7718-0.896)	1,26E-06
IE1A	All subjects	6	RS3763349	32 808 232	G	0.8335 (0.7736-0.898)	1,70E-06
IE1A	All subjects	6	RS9276810	32 810 443	A	0.83 (0.7702-0.8944)	1,02E-06
IE1A	All subjects	6	RS6924102	32 811 383	G	0.8378 (0.777-0.9034)	4,15E-06
IE1A	All subjects	6	**RS1480380**	32 913 246	A	0.6676 (0.5841-0.763)	**3,02E-09**
IE1A	All subjects	6	**RS9276933**	32 930 795	G	0.6931 (0.6095-0.7882)	**2,31E-08**
IE1A	All subjects	6	RS423196	33 003 562	G	0.7726 (0.6948-0.8592)	1,95E-06
IE1A	All subjects	6	RS406477	33 005 644	G	0.7699 (0.6928-0.8556)	1,21E-06
IE1A	All subjects	6	RS987870	33 042 880	G	0.7473 (0.6606-0.8453)	3,61E-06
IE1A	All subjects	6	RS2071354	33 044 388	G	0.7475 (0.6608-0.8455)	3,66E-06
IE1A	All subjects	6	**RS6914616**	33 063 592	A	0.582 (0.4919-0.6886)	**2,82E-10**
IE1A	All subjects	6	**RS2179915**	33 065 734	A	0.5821 (0.492-0.6887)	**2,86E-10**
IE1A	All subjects	6	RS3128930	33 075 666	A	0.8228 (0.7559-0.8956)	6,58E-06
IE1A	All subjects	6	**RS6924545**	33 077 607	A	0.5928 (0.5011-0.7013)	**1,07E-09**
IE1A	All subjects	6	RS12529876	167 461 501	A	1.185 (1.099-1.277)	8,94E-06
IE1A	All subjects	6	RS4710181	167 513 998	G	1.183 (1.099-1.273)	7,74E-06
IE1A	All subjects	6	RS6907666	167 523 395	A	1.192 (1.106-1.285)	4,72E-06
IE1A	All subjects	10	RS4132050	12 797 758	A	0.8261 (0.7647-0.8924)	1,22E-06
IE1A	All subjects	10	RS2147268	105 793 459	G	0.8196 (0.7504-0.8952)	9,99E-06

IE1A	All subjects	13	RS2991478	79 754 928	A	0.7325 (0.6439-0.8333)	2,23E-06
IE1A	All subjects	13	RS2988030	79 762 645	A	0.7292 (0.6411-0.8293)	1,52E-06
IE1A	All subjects	14	RS7157970	105 463 936	A	1.196 (1.105-1.295)	9,85E-06
IE1A	All subjects	18	RS11872992	58 040 587	A	0.7858 (0.7094-0.8704)	3,82E-06
IE1A	All subjects	22	RS5753103	30 768 777	A	0.8411 (0.7812-0.9055)	4,28E-06
IE1A	All subjects	22	RS4820845	30 800 338	C	0.8291 (0.7699-0.8929)	7,29E-07
IE1A	All subjects	22	RS5997641	30 809 342	A	0.8251 (0.7602-0.8956)	4,33E-06
IE1A	All subjects	22	RS1061660	30 819 628	G	0.827 (0.7623-0.8973)	5,05E-06
IE1B	MS cases	3	RS7628773	24723928	A	1.285 (1.15-1.436)	9,12E-06
IE1B	MS cases	3	RS7629359	24727377	G	1.293 (1.157-1.445)	5,84E-06
IE1B	MS cases	10	RS1466196	130749975	A	1.267 (1.142-1.405)	7,50E-06
IE1B	Controls	1	RS6424074	3 317 780	G	0.7713 (0.6887-0.8639)	7,08E-06
IE1B	Controls	14	RS10149279	80 579 214	C	1.332 (1.179-1.504)	3,77E-06
IE1B	Controls	15	RS17114996	25 245 502	A	2.219 (1.559-3.158)	9,52E-06
IE1B	Controls	15	RS8038349	25 270 440	A	2.226 (1.561-3.173)	9,81E-06
IE1B	Controls	15	RS17115045	25 272 862	G	2.221 (1.561-3.161)	9,28E-06
IE1B	Controls	15	RS8035400	25 273 478	A	2.221 (1.56-3.16)	9,34E-06
IE1B	Controls	15	RS17115126	25 303 191	G	2.221 (1.561-3.161)	9,28E-06
IE1B	Controls	15	RS7171937	25 329 547	A	2.218 (1.559-3.157)	9,58E-06
IE1B	Controls	19	RS4926207	14 473 036	G	0.7728 (0.6905-0.865)	7,44E-06
IE1B	Controls	20	RS6089729	60 890 564	A	0.7377 (0.6447-0.8443)	9,85E-06
IE1B	All subjects	6	RS2516454	31420863	T	1.537 (1.271-1.86)	9,74E-06
IE1B	All subjects	6	RS2534678	31463963	A	1.399 (1.227-1.594)	5,00E-07
IE1B	All subjects	6	RS2534667	31468099	A	1.427 (1.239-1.643)	8,16E-07
IE1B	All subjects	6	RS2534657	31472459	A	1.401 (1.228-1.598)	5,12E-07
IE1B	All subjects	6	RS2844496	31480398	A	1.466 (1.259-1.707)	8,07E-07
IE1B	All subjects	6	RS2246986	31482203	G	1.47 (1.263-1.711)	6,47E-07
IE1B	All subjects	6	RS2844509	31510924	G	1.297 (1.168-1.439)	9,79E-07
IE1B	All subjects	6	RS2239705	31513402	A	1.313 (1.168-1.476)	5,17E-06
IE1B	All subjects	6	RS511027	32206687	A	1.432 (1.244-1.649)	6,01E-07
IE1B	All subjects	6	RS9268557	32389305	G	0.84 (0.778-0.9069)	8,24E-06
IE1B	All subjects	15	RS2397382	95523004	A	0.8132 (0.746-0.8864)	2,60E-06
IE1B	All subjects	22	RS442656	21416442	A	0.7812 (0.7016-0.8697)	6,60E-06
101K	MS cases	5	RS1347135	167484417	G	1.302 (1.16-1.461)	7,47E-06
101K	Controls	5	RS966297	23878207	A	0.7551 (0.6671-0.8547)	8,87E-06
101K	Controls	6	RS2844571	31335647	G	0.7567 (0.6739-0.8496)	2,39E-06
101K	Controls	6	RS2253908	31336891	A	0.7571 (0.674-0.8503)	2,65E-06

101K	Controls	6	RS2844545	31345229	A	0.7659 (0.6832-0.8586)	4,80E-06
101K	Controls	6	RS2269475	31583931	A	1.429 (1.229-1.662)	3,66E-06
101K	Controls	6	RS3763295	31587938	G	1.456 (1.249-1.696)	1,46E-06
101K	Controls	6	RS2242657	31602489	G	1.409 (1.212-1.638)	8,17E-06
101K	Controls	6	RS2295665	31632686	A	1.427 (1.227-1.66)	3,95E-06
101K	Controls	6	RS2295664	31633165	A	1.417 (1.219-1.647)	5,84E-06
101K	Controls	6	RS14365	31635710	G	1.424 (1.235-1.643)	1,25E-06
101K	Controls	6	RS2280800	31646398	A	1.41 (1.213-1.64)	8,00E-06
101K	Controls	6	RS2242653	31675765	A	1.407 (1.215-1.631)	5,36E-06
101K	Controls	6	RS2293861	31711124	A	1.441 (1.235-1.681)	3,51E-06
101K	Controls	6	RS2075788	31712181	C	1.439 (1.233-1.679)	3,81E-06
101K	Controls	6	RS3828922	31713454	A	1.44 (1.234-1.68)	3,72E-06
101K	Controls	6	RS6905572	31731881	A	1.436 (1.224-1.684)	8,83E-06
101K	Controls	6	RS2294878	32367795	A	1.316 (1.178-1.471)	1,25E-06
101K	Controls	6	RS7774434	32657578	G	1.299 (1.159-1.456)	7,09E-06
101K	Controls	6	RS9275428	32670978	G	1.317 (1.166-1.487)	9,19E-06
101K	Controls	15	RS11637194	80494256	G	0.7656 (0.6822-0.8592)	5,62E-06
101K	Controls	15	RS2028119	80514164	C	0.7787 (0.6973-0.8696)	9,05E-06
101K	Controls	20	RS775121	16046227	A	0.7348 (0.6412-0.842)	9,26E-06
101K	Controls	20	RS1467704	55441072	G	0.742 (0.6508-0.8459)	8,17E-06

101K	All subjects	6	RS3116798	29671991	G	1.227 (1.122-1.343)	7,36E-06
101K	All subjects	6	RS1611364	29679602	A	1.241 (1.135-1.357)	2,19E-06
101K	All subjects	6	RS1362126	29691019	A	0.8418 (0.7804-0.908)	8,38E-06
101K	All subjects	6	RS2523402	29699156	G	0.8395 (0.7784-0.9054)	5,71E-06
101K	All subjects	6	RS2394159	29702679	G	0.8374 (0.7762-0.9034)	4,61E-06
101K	All subjects	6	RS2735051	29705688	A	0.8394 (0.7782-0.9053)	5,66E-06
101K	All subjects	6	RS2735048	29733701	A	0.8357 (0.7724-0.9043)	8,14E-06
101K	All subjects	6	RS2735046	29734098	A	0.8379 (0.7752-0.9055)	8,04E-06
101K	All subjects	6	RS2523409	29775662	G	0.8316 (0.769-0.8993)	3,87E-06
101K	All subjects	6	RS3128912	29815637	A	1.254 (1.137-1.383)	5,88E-06
101K	All subjects	6	RS2523822	29828660	G	0.8293 (0.7648-0.8993)	6,04E-06
101K	All subjects	6	RS3094170	29829265	A	1.252 (1.136-1.381)	6,64E-06
101K	All subjects	6	RS3094165	29833541	A	1.214 (1.12-1.315)	2,34E-06
101K	All subjects	6	RS2860580	29906691	A	1.185 (1.1-1.276)	8,09E-06
101K	All subjects	6	RS1616549	29914270	A	1.255 (1.138-1.384)	5,19E-06
101K	All subjects	6	RS3823355	29942083	A	0.8322 (0.7676-0.9023)	8,38E-06
101K	All subjects	6	RS3823358	29942205	A	0.829 (0.7643-0.8991)	6,01E-06
101K	All subjects	6	RS6904029	29943067	A	0.8314 (0.7668-0.9013)	7,49E-06
101K	All subjects	6	RS4713281	29978352	A	0.8301 (0.7653-0.9004)	7,16E-06
101K	All subjects	6	RS9357092	29984252	A	0.8264 (0.7619-0.8963)	4,21E-06

101K	All subjects	6	RS3910312	30008746	C	0.8299 (0.7651-0.9001)	6,87E-06
101K	All subjects	6	RS9380150	30010492	G	0.8317 (0.7668-0.902)	8,64E-06
101K	All subjects	6	RS9295829	30028800	G	0.8308 (0.766-0.9011)	7,78E-06
101K	All subjects	6	RS3807031	30033884	A	0.8121 (0.7434-0.887)	3,82E-06
101K	All subjects	6	RS9393989	30040084	A	0.83 (0.7652-0.9003)	7,09E-06
101K	All subjects	6	RS4959041	30077967	G	0.8227 (0.7586-0.8923)	2,43E-06
101K	All subjects	6	RS9261438	30089280	A	0.8258 (0.7617-0.8952)	3,39E-06
101K	All subjects	6	RS9261639	30237859	A	0.8275 (0.761-0.8997)	9,34E-06
101K	All subjects	6	RS2844571	31335647	G	0.8282 (0.7648-0.8969)	3,53E-06
101K	All subjects	6	RS2253908	31336891	A	0.8289 (0.7653-0.8978)	4,05E-06
101K	All subjects	6	RS9266629	31346822	G	0.7942 (0.7246-0.8705)	8,63E-07
101K	All subjects	6	RS7741091	31352631	G	1.212 (1.115-1.318)	6,07E-06
101K	All subjects	6	RS2516448	31390410	G	1.181 (1.097-1.272)	9,34E-06
101K	All subjects	6	RS3763295	31587938	G	1.266 (1.141-1.405)	8,36E-06
101K	All subjects	6	RS2295665	31632686	A	1.263 (1.14-1.401)	8,69E-06
101K	All subjects	6	RS2295664	31633165	A	1.261 (1.138-1.398)	9,98E-06
101K	All subjects	6	RS2293861	31711124	A	1.277 (1.15-1.419)	5,05E-06
101K	All subjects	6	RS2075788	31712181	C	1.275 (1.148-1.416)	5,76E-06
101K	All subjects	6	RS3828922	31713454	A	1.276 (1.149-1.418)	5,29E-06
101K	All subjects	6	RS481825	31780594	A	0.6587 (0.5533-0.7842)	2,71E-06
101K	All subjects	6	RS35294112	31794443	G	0.5835 (0.471-0.7229)	8,25E-07
101K	All subjects	6	RS17201248	31803130	A	0.5826 (0.4706-0.7213)	7,06E-07
101K	All subjects	6	RS391755	32192436	G	0.6883 (0.588-0.8057)	3,32E-06
101K	All subjects	6	RS397081	32192617	G	0.6277 (0.5258-0.7493)	2,56E-07
101K	All subjects	6	RS2294878	32367795	A	1.203 (1.115-1.299)	2,06E-06
101K	All subjects	6	RS660895	32577380	G	1.245 (1.136-1.364)	2,98E-06
101K	All subjects	6	RS7774434	32657578	G	1.207 (1.116-1.306)	2,90E-06
101K	All subjects	6	RS9275245	32660943	G	1.192 (1.104-1.287)	6,40E-06
101K	All subjects	6	RS6457617	32663851	A	1.207 (1.119-1.302)	1,15E-06
101K	All subjects	6	RS9275312	32665728	G	1.264 (1.145-1.394)	3,15E-06
101K	All subjects	6	RS9275328	32666822	A	1.267 (1.148-1.398)	2,51E-06
101K	All subjects	6	RS9275330	32666875	G	1.266 (1.147-1.396)	2,77E-06
101K	All subjects	6	RS9275333	32666968	G	1.262 (1.143-1.392)	3,73E-06
101K	All subjects	6	RS9275371	32668296	G	1.254 (1.153-1.364)	1,26E-07
101K	All subjects	6	RS9275374	32668526	A	1.256 (1.155-1.366)	9,54E-08
101K	All subjects	6	RS9275388	32669084	G	1.255 (1.154-1.365)	1,06E-07
101K	All subjects	6	RS9275390	32669156	G	1.258 (1.157-1.369)	8,23E-08
101K	All subjects	6	RS9275393	32669439	A	1.255 (1.153-1.365)	1,28E-07
101K	All subjects	6	RS9275406	32669955	A	1.255 (1.154-1.364)	1,18E-07
101K	All subjects	6	RS9275407	32670037	A	1.255 (1.154-1.365)	1,08E-07
101K	All subjects	6	RS9275418	32670244	G	1.255 (1.154-1.365)	1,07E-07

101K	All subjects	6	RS9275424	32670576	G	1.256 (1.155-1.366)	1,01E-07
101K	All subjects	6	RS9275428	32670978	G	1.256 (1.155-1.366)	1,08E-07
101K	All subjects	6	RS9275439	32671521	G	1.255 (1.154-1.366)	1,16E-07
101K	All subjects	8	RS900896	68528197	G	1.21 (1.112-1.317)	9,59E-06
101K	All subjects	12	RS11170018	52550467	A	1.236 (1.126-1.356)	8,05E-06
101K	All subjects	20	RS6097106	36589678	A	0.6993 (0.6004-0.8143)	4,18E-06
